# Carbamate Prodrugs Restrict *In Vivo* Metabolism and Improve the Pharmacokinetics of Isoniazid

**DOI:** 10.1021/acscentsci.5c00576

**Published:** 2025-07-09

**Authors:** Jishnu Sankar, Manish Kumar Bajpai, Anjali Chauhan, Ravi Maddheshiya, Nidhi Sharma, Aditya Sharma, Yashwant Kumar, Dinesh Mahajan

**Affiliations:** † Centre for Drug Discovery, 145787 BRIC-Translational Health Science and Technology Institute, Faridabad, Haryana 121001, India; ‡ Manipal Academy of Higher Education, Manipal, Karnataka 576104, India

## Abstract

Isoniazid (INH), an important first-line drug in tuberculosis (TB) treatment, faces significant challenges primarily due to hepatotoxicity and peripheral neuropathy as major side effects. These adverse effects often lead to poor patient compliance and treatment dropouts. The INH’s *in vivo* metabolism is responsible for these adverse effects. INH’s reactive terminal −NH_2_ group is involved in its undesired *in vivo* metabolic transformations. To address this, we designed and synthesized carbamate-based prodrugs of INH by masking the −NH_2_ group to reduce its metabolic activity. Herein, we report our efforts to develop such prodrugs and their impact on *in vivo* metabolism and the pharmacokinetic profile of free INH. The *ex vivo* stability, bioconversion, and *in vivo* pharmacokinetic profile with detailed metabolite analysis of these prodrugs were determined in mice. The lead prodrug **1d** demonstrated enhanced systemic exposure of free INH (1.5-fold, AUC ≈ 3948 ng·h/mL), reduced formation of undesired metabolites, and prolonged half-life (1.3-fold, *t*_1/2_ ≈ 0.88 h) compared to naive INH. This prodrug approach represents a promising strategy for safer and more effective TB therapy, with the potential for less frequent dosing and improved patient compliance.

## Graphical abstract

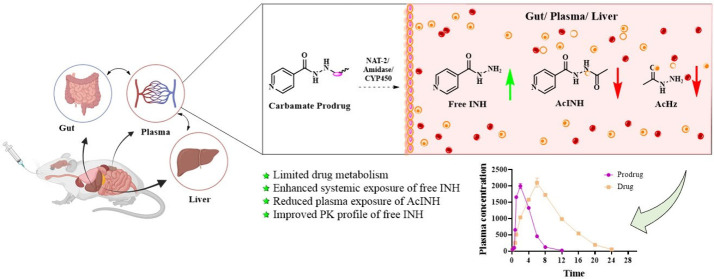

## Introduction

Isoniazid (INH) is a key component of frontline treatment for drug-susceptible tuberculosis (TB), frequently used in combination with pyrazinamide (PZA), rifampicin (RIF), and ethambutol (ETA).^1^ INH is also commonly prescribed for prophylactic treatment for latent TB infection.^2^ It is orally bioavailable and undergoes rapid metabolism in the gut, liver, and plasma. Despite its potent pharmacological activity, INH treatment is often accompanied by significant adverse effects such as drug-metabolism-associated hepatotoxicity and peripheral neuropathy, which hinder its consistent use in clinical settings. We recently reviewed these facts and highlighted that INH’s highly nucleophilic and chemically reactive terminal −NH_2_ group is a key contributor to its metabolism.^3^ This reactive terminal −NH_2_ also interacts with various endogenous biomolecules *in vivo* to form undesired covalent adducts, leading to the depletion of the drug and these important endogenous biomolecules. Metabolic degradation of INH in humans proceeds through two main pathways: acetylation of the −NH_2_ group by *N*-acetyltransferase (NAT) enzymes and hydrolysis of the amide linkage by amidases. This process generates metabolites, such as acetyl isoniazid (AcINH), isonicotinic acid (INA), diacetyl hydrazine (DiAcHz), acetyl hydrazine (AcHz), and hydrazine (Hz). Notably, AcINH, AcHz, and Hz undergo further oxidation by CYP450 enzymes, producing toxic reactive intermediates that contribute to liver cell necrosis and hepatotoxicity.^4–7^ Additionally, INH reacts nonenzymatically with endogenous biomolecules such as vitamin B_6_ via its −NH_2_ group, forming hydrazones that deplete free vitamin B_6_, leading to peripheral neuropathy. The incidence of INH-related hepatotoxicity is estimated to be between 0.05% and 1%, while peripheral neuropathy occurs in approximately 2% to 6.5% of the global TB population.^4^ Although these rates appear low, they represent a substantial number of affected individuals given the high worldwide prevalence of TB infection. We proposed that plasma-labile derivatives or prodrugs of INH, formed by chemical modification of the terminal −NH_2_ group, may reduce host-mediated, undesired metabolic transformations.^3^ The present study outlined our efforts to develop plasma-labile INH prodrugs to mitigate undesired INH metabolism. Two central questions guide our investigation. First, can low molecular weight carbamate derivatives of INH effectively hydrolyze and release free INH into the systemic circulation following the action of hydrolytic enzymes in the gastrointestinal (GI) tract, liver, or plasma? Second, can these derivatives achieve favorable systemic exposure of INH while limiting the formation of undesired metabolites, thereby altering the pharmacokinetic profile of INH in mice due to their distinct labile chemical structures and masked terminal −NH_2_ group? Accordingly, the study was conceptualized with three main objectives. The first objective was to synthesize chemically stable but biologically labile derivatives of INH. The second objective was to develop a suitable bioanalytical method for the simultaneous quantification of the new derivatives, INH, and its known undesired metabolites (AcINH, INA, and AcHz). The third objective was to evaluate these biologically labile derivatives in healthy mice through pharmacokinetic analysis to determine the effect on the plasma concentration of released INH and its various metabolites.

We synthesized 10 prodrugs of INH by masking its terminal −NH_2_ group with low molecular weight substitutions via a carbamate linkage. The structural integrity of these new compounds was confirmed using NMR spectroscopy and mass spectrometry, and their chemical purity was ensured through HPLC analysis. Additionally, the physicochemical properties of each compound were determined experimentally and compared to those of INH. A suitable LC-MS method was developed to facilitate biochemical analysis for the simultaneous quantification of INH, AcINH, INA, and AcHz. To evaluate bioreversibility, each compound was incubated with mouse and human plasma under *ex vivo* conditions, followed by oral pharmacokinetic studies in mice to measure plasma concentrations of released INH and its metabolites in single and repeat dosing studies, using naïve INH as a control. Plasma exposure levels of INH released *in vivo* from one lead prodrug were assessed at three incremental doses. Subsequently, complete pharmacokinetic profiling of a few selected leads and INH was conducted. These results are presented in detail in the following sections.

## Results

### Chemistry

The prodrug approach has been widely used in the development of numerous drug molecules. Carbamate linker-based prodrugs have achieved considerable success, with several FDA-approved medicines utilizing this strategy.^8,9^ We designed and synthesized a series of 10 carbamate-based derivatives of INH. Through optimization efforts, a reaction of INH with various alkyl chloroformates in a THF–water solvent system yielded the corresponding nonpolar derivatives of INH, **1a**–**1j** (Figure 1). The lipophilicity of all the new compounds was determined by the shake flask method.^10^ The logD value of new compounds **1a**–**1j** varied from −0.2 to 1.1 (Table 1), whereas INH was found to have a logD value of −0.8.^11^ The predicted lipophilicity values varied from −0.4 to 1.4 for the new compounds, which matched closely with their respective experimental values. All compounds **1a**–**1j** were assessed for their aqueous solubility in Tris HCl buffer (pH 7.4) using the shake flask method. The chemical stability of compounds **1a**–**1j** was evaluated in neat DMSO using a UV spectrophotometer. The aqueous stability of **1a**–**1j** was determined in acidic buffer (pH ≈ 3.2) and basic buffer (pH ≈ 7.6) using HPLC. Stability was determined based on the concentration of remaining intact prodrugs, after 24 h incubation. All newly synthesized prodrugs were found to be stable in DMSO and basic buffer except **1j**. Only three prodrugs, i.e., **1b**, **1c**, and **1d**, exhibited limited hydrolysis under acidic conditions (low pH), as summarized in Table 1.

**Figure 1:**
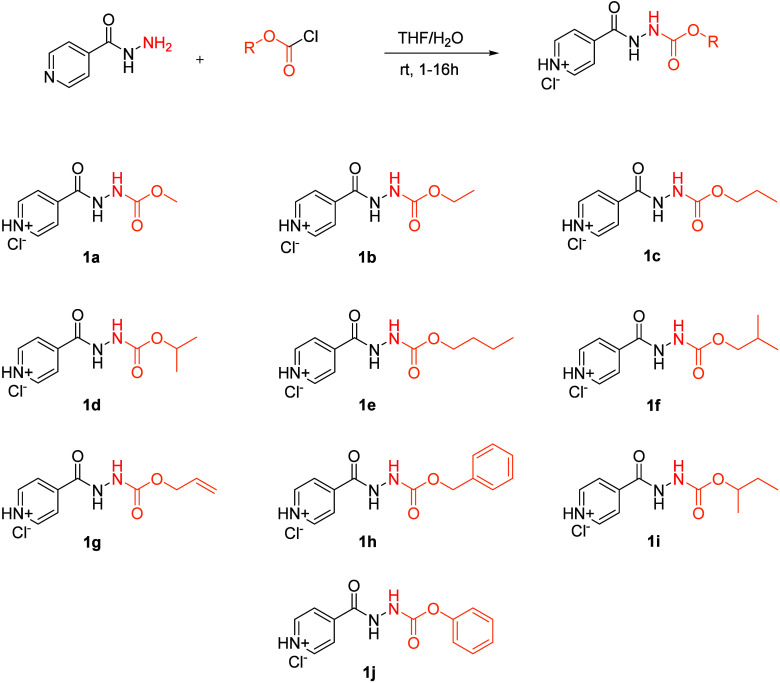
Synthetic scheme and structures of prodrugs **1a**–**1j** as their corresponding HCl salts.

**Table 1:** Physicochemical Parameters of INH and Prodrugs of INH

					chemical stability^d^		
compound (MW)^a^	p*K*_a_ ^c^	logD^b^	logP^c^	solubility (mg/mL)^b^	DMSO	pH (3.0)	pH (7.6)
INH (137.1)	11.2	–0.8	–0.1	609.9	100.0	100.0	100.0
**1a** (231.6)	8.2	–0.2	–0.4	213.2	100.0	100.0	100.0
**1b** (245.7)	8.3	–0.01	–0.01	326.2	100.0	89.7	100.0
**1c** (259.7)	8.3	0.2	0.5	86.0	100.0	91.9	100.0
**1d** (259.7)	8.3	0.2	0.3	18.6	100.0	93.6	100.0
**1e** (273.7)	8.3	0.8	0.9	49.4	100.0	100.0	100.0
**1f** (273.7)	8.2	0.9	0.9	29.3	100.0	98.17	100.0
**1g** (257.7)	8.2	0.2	0.3	402.2	100.0	96.9	100.0
**1h** (307.7)	8.2	1.1	1.4	0.4	100.0	100.0	100.0
**1i** (273.7)	8.3	0.8	0.8	110.6	100.0	100.0	98.3
**1j** (293.7)	7.6	0.9	1.32	12.4	100.0	94.6	58.2

a MW, molar weight.

b Experimental value.

c Predicted value.

d Percentage of parent remaining post 24 h of incubation.

### LCMS-Based Bioanalytical Method Development

The primary objective of this study was to investigate the effects of various carbamate-based prodrugs on the *ex vivo* and *in vivo* release of INH and its host-mediated metabolic biotransformation. To accomplish this, a robust and reliable bioanalytical method is essential for the quantification of INH and its metabolites. Although several studies in the literature describe methods for the simultaneous quantification of INH and its metabolites using LC-MS, these methods typically require the derivatization of either all metabolites or at least one metabolite.^12–14^ The derivatization step introduces additional complexity to the analytical workflow, making it resource-intensive and impractical, especially for experiments involving large sample sizes or multiple time points. Notably, no derivatization-free LC-MS-based methods were identified in the literature to simultaneously quantify INH, AcINH, INA, and AcHz from biological samples. To address this gap, a novel LC-MS method was developed and validated, capable of simultaneously quantifying INH, AcINH, INA, and AcHz, along with the parent derivatives **1a**–**1j** (Table 2 and Tables [Table tbl3A] and [Table tbl3B]). To the best of our knowledge, this is the first derivatization-free LC-MS method that enables simultaneous quantification of these compounds from a biological matrix. The reliability, reproducibility, and quality of data generated from any bioanalytical method depend on rigorous validation. Consequently, this new method was validated following the US FDA bioanalytical method validation guidelines,^15^ using mouse plasma as the biological matrix. Validation parameters included selectivity, stability, recovery, matrix effects, linearity, accuracy, and precision (Tables [Table tbl3A] and [Table tbl3B]). Detailed results of the method validation are available in the Supporting Information. This method offers a streamlined and efficient approach to analyze INH and its metabolites, supporting further studies of prodrugs and their biotransformation.

**Table 2:** LCMS Parameters Used for the Simultaneous Quantification of INH, AcINH, INA, and AcHz without Any Derivatization

					quality controls				
analyte	MRM^a^ (*m*/*z*)	DP^a^	CE^a^	calibration range (ng/mL)	LLOQ^a^	LQC^a^	MQC^a^	HQC^a^	HLOQ^a^
INH	138.2→121.2	80	20	1.563–100	1.563	5	40	75	100
AcINH	180.1→138.1	60	20	0.195–100	0.195	0.781	40	75	100
INA	124.2→96.1	115	35	5–100	5	15	40	75	100
AcHz	75.2→58.1	47	35	50–500	50	150	200	400	500
IS^b^	123.2→80.1	105	30	NA	NA	NA	NA	NA	NA

a MRM, Multiple Reaction Monitoring; DP, Deceleration Potential; CE, Collision Energy; LLOQ, Lower Limit of Quantification; LQC, Lower-Quality Control; MQC, Medium-Quality Control; HQC, Higher-Quality Control; HLOQ, Highest Level Of Quantification.

b Nicotinamide was used as IS;^16^ NA, not applicable.

**3A tbl3A:** Method Validation Results for the Simultaneous Quantification of INH, AcINH, INA, and AcHz[Table-fn tbl3Afn1]

				stability (% of accuracy; *n* = 5)				
analyte	QC level	linearity (R^2^)	selectivity (%) (*n* = 6)	autosampler (5 °C)	benchtop	carryover (%) (*n* = 5)	matrix effect (%CV of MF value; *n* = 6)	recovery (%) (*n* = 4)
INH	LLOQ	0.9965	3.5 ± 1.1	NA	NA	3.7 ± 0.5	NA	NA
	LQC		NA	–1.5	6.0	NA	9.7	86.2 ± 5.4
	MQC		NA	–2.7	–0.9	NA	NA	97.0 ± 7.0
	HQC		NA	7.8	7.1	NA	6.0	101.9 ± 8.5
AcINH	LLOQ	0.9971	4.3 ± 3.7	NA	NA	5.6 ± 1.5	NA	NA
	LQC		NA	–1.1	5.6	NA	11.8	81.2 ± 0.6
	MQC		NA	11.1	11.7	NA	NA	94.5 ± 8.2
	HQC		NA	9.2	16.7	NA	6.4	111.0 ± 6.0
INA	LLOQ	0.9975	5.2 ± 1.4	NA	NA	5.5 ± 1.9	NA	NA
	LQC		NA	–1.8	–8.6	NA	10.3	85.3 ± 4.4
	MQC		NA	5.2	2.2	NA	NA	85.9 ± 3.9
	HQC		NA	1.5	9.0	NA	6.5	90.4 ± 5.2
AcHz	LLOQ	0.9873	9.1 ± 4.9	NA	NA	8.9 ± 4.1	NA	NA
	LQC		NA	–8.5	8.2	NA	13.5	88.8 ± 6.0
	MQC		NA	–12.4	2.8	NA	NA	100.2 ± 10.0
	HQC		NA	0.4	17.5	NA	6.3	90.5 ± 9.0

a NA, not applicable.

**3B tbl3B:** Method Validation Results for the Simultaneous Quantification of INH, AcINH, INA, and AcHz

		precision (RSD%; *n* = 5)[Table-fn tbl3Bfn1]					accuracy (RE%; *n* = 5)[Table-fn tbl3Bfn1]				
analyte	concentration level	intra day	intra day	inter day	inter day	inter day	intra day	intra day	inter day	inter day	inter day
INH	LLOQ	19.4	18.6	20.9	10.9	12.1	–13.8	–11.2	–15.5	–4.7	–4.9
	LQC	7.8	11.2	4.3	8.7	1.2	1.3	7.0	2.0	10.3	–6.0
	MQC	4.0	8.2	14.3	11.7	10.8	–8.0	–9.5	–9.0	–2.7	5.8
	HQC	10.2	8.1	7.8	4.8	11.9	–0.5	–2.8	–1.2	12.1	3.2
AcINH	LLOQ	17.3	20.2	8.4	14.4	9.6	–6.2	33.3	17.7	16.7	–9.8
	LQC	11.0	15.3	3.4	9.0	4.7	0.3	1.6	3.6	0.2	–14.3
	MQC	3.7	5.4	12.9	15.6	15.4	–8.0	–8.6	–11.2	–1.0	12.3
	HQC	6.9	6.8	7.4	4.9	13.7	0.1	–0.5	–3.1	5.9	9.5
INA	LLOQ	14.3	15.5	14.0	12.6	19.0	8.8	6.4	11.0	9.6	0.8
	LQC	7.2	10.2	7.0	8.4	3.3	–39.2	–14.6	–12.5	–27.7	–14.2
	MQC	6.2	6.4	8.5	12.0	11.6	–12.5	–11.7	–14.6	–8.7	9.8
	HQC	8.2	7.5	9.2	7.0	13.0	–4.9	–6.6	–11.3	2.5	–2.7
AcHz	LLOQ	12.6	13.9	20.2	12.4	10.2	–13.6	–2.8	–7.5	–5.4	19.1
	LQC	11.7	14.7	5.1	6.9	4.4	4.3	4.1	–7.9	–0.3	11.2
	MQC	8.9	10.0	12.8	14.5	14.2	4.5	7.8	2.6	10.4	5.9
	HQC	9.4	8.4	13.5	2.0	12.3	3.2	2.2	0.6	12.8	–2.5

a RSD, relative standard deviation; RE, relative error.

### LCMS-Based Bioanalytical Method Validation

As part of the method validation process, all parameters were evaluated in four to six replicates. Plasma samples from different individual mouse plasma matrix sources were used for each analysis. The method’s selectivity was assessed by comparing plasma samples from six different individual sources, spiked with four analytes, i.e., INH, AcINH, INA, and AcHz, at their LLOQ (Lower Limit of Quantification) levels to blank plasma samples. No significant interference was observed at the analytes’ retention times, with responses in blank and spiked samples (Table [Table tbl3A]). Carryover analysis at the LLOQ level indicated values ranging from approximately 3% to 14.2%, which were within acceptable thresholds. Linearity was assessed through calibration curve analysis, producing R^2^ values of 0.9965, 0.9971, 0.9975, and 0.9873 for INH, AcINH, INA, and AcHz, respectively. Intraday and interday precision and accuracy for all analytes were evaluated at four concentration levels: LLOQ, LQC (Low-Quality Control), MQC (Medium-Quality Control), and HQC (High-Quality Control). Results for both precision and accuracy met the established acceptance criteria (Table [Table tbl3B]). The matrix effect was assessed by calculating the coefficient of variation (CV) of matrix factor (MF) values at the LQC and HQC levels. The findings demonstrated limited ion suppression, confirming matrix effects within an acceptable range. Recovery values for all four analytes ranged between 80% and 120%. Recovery was determined by comparing the peak areas of analytes in extracted samples with those in postextraction spiked samples at LQC, MQC, and HQC concentration levels. Stability testing confirmed that all analytes remained stable under multiple conditions, including storage at 5 °C as in autosampler settings and at room temperature (benchtop conditions) across LQC, MQC, and HQC levels. These results validate the method as robust, reliable, and suitable for quantification of the four analytes under the tested conditions.

### Bioconversion of Prodrugs in Plasma

Plasma is a rich source of hydrolytic enzymes, including amidases and esterases, which play a critical role in influencing the stability and activation of prodrugs.^17,18^ *Ex vivo* experiments were conducted to evaluate the bioreversibility of the prodrugs by incubating them with heparinized plasma of mice and humans at 37 °C for 20 h. All prodrugs were labile under these conditions, as evidenced by the percentage of parent molecules remaining after 20 h of incubation (Table 4). However, the rates of conversion varied among the compounds. In mouse plasma, compounds **1c**, **1e**, **1g**, and **1h** were highly labile, with minimal or below quantifiable levels of the parent molecule detected. Similarly, compounds **1c**, **1e**, **1f**, **1g**, and **1h** exhibited significant lability in human plasma. Compounds **1a** and **1j** showed moderate stability in mouse plasma, retaining 70.5% and 85.5% of their original forms, respectively. However, their stability was substantially lower in human plasma. Only 40.1% of **1a** remained intact, while **1j** degraded to levels below the quantification limit. The concentration of released INH over the 20 h incubation period was evaluated based on their respective estimated exposures (eAUC). In mouse plasma, prodrugs **1i** and **1j** demonstrated the highest INH release, with eAUC values of 1744 and 1730 μg·h/mL, respectively. However, their performance in human plasma was significantly poor, with eAUC values of 102 and 4.0 μg·h/mL, respectively. This marked decrease in INH release highlights the species-specific differences in the metabolism of these two molecules.

**Table 4:** Plasma Lability and Estimated Plasma Exposure (eAUC_20m–20h_)­^a^ of **1a**–**1j** and released INH after 20 h *Ex Vivo* Incubation of the Respective Prodrug (200 μg/mL) in Mice and Human Plasma^b^ ^,^ ^c^

		% of prodrug remaining at 20 h		eAUC_20m–20h_ in mice plasma		eAUC_20m–20h_ in human plasma	
compound	mol. eq of INH	mice plasma	human plasma	prodrug	INH	prodrug	INH
INH	1	NA	NA	NA	1563 ± 1506	NA	2,107 ± 1650
**1a**	0.59	70.5	40.1	1650 ± 930	38 ± 9	1418 ± 824	49 ± 18
**1b**	0.56	13.8	47.5	1214 ± 758	65 ± 46	1402 ± 957	67 ± 60
**1c**	0.53	1.4	0.8	551 ± 81	43 ± 25	860 ± 131	66 ± 41
**1d**	0.53	23.8	40.1	987 ± 154	257 ± 386	1196 ± 308	572 ± 306
**1e**	0.50	1.2	0.8	180 ± 36	38 ± 9	640 ± 116	63 ± 37
**1f**	0.50	13.2	0.4	382 ± 302	53 ± 28	854 ± 615	84 ± 82
**1g**	0.53	BLQ	BLQ	168 ± 91	28 ± 3	530 ± 316	26 ± 2
**1h**	0.44	2.9	1.7	87 ± 85	117 ± 67	465 ± 426	131 ± 72
**1i**	0.58	63.8	89.1	1486 ± 35	1744 ± 429	2788 ± 217	102 ± 14
**1j**	0.53	85.5	BLQ	7347 ± 655	1730 ± 274	752 ± 355	4 ± 7

a In ng·h/mL.

b Aliquots were withdrawn at *t* = 20 m, 60 m, 120 m, and 20 h.

c Concentration levels of AcINH were BLQ; NA, not applicable.

Among all the tested prodrugs, compound **1d** demonstrated notable INH release in both mouse and human plasma, achieving the highest exposure levels in human plasma (eAUC: 572 μg·h/mL). Based on this bioconversion data, compound **1d** emerged as the preferred candidate, exhibiting consistent and reliable performance across two species. In contrast, **1i** and **1j** showed limited INH exposure in human plasma, making them less suitable for further development. Consequently, compounds **1i** and **1j** were avoided from further investigations (except oral plasma exposure study in mice; Figure 2 and Table 5, *vide infra*) due to their differential bioconversion in two species.

**Figure 2:**
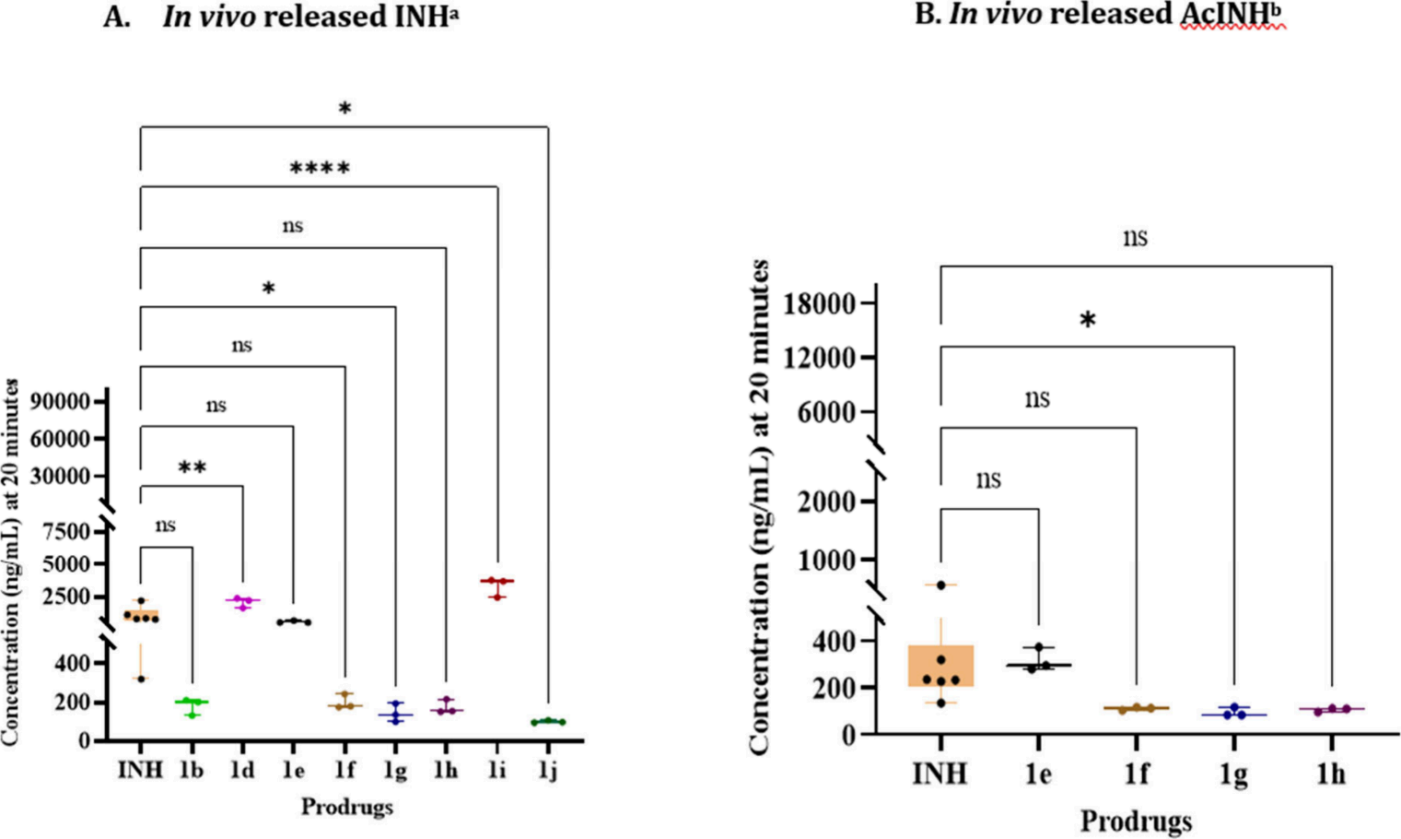
Plasma concentration of *in vivo* released free INH (A) and AcINH (B) at 20 min post oral administration of INH and prodrugs **1a**–**1j** in a single oral dose (10 mg/kg) administration to Balb/C mice (*n* = 3). Data are expressed as the mean ± SD, with statistics quantified using one-way ANOVA with Dunnett’s multiple comparison test. *p ≤ 0.05; **p ≤ 0.01; ****p ≤ 0.0001; ns, *p* is nonsignificant. ^a^ *In vivo* released INH from prodrugs **1a**, **1c** reported as BLQ. ^b^ *In vivo* released AcINH from prodrugs **1a**, **1b**, **1c**, **1d**, **1i**, and **1j** were reported as BLQ.

**Table 5:** Plasma eAUC_20m–120m_ ^a^ of Free INH and AcINH Formed *In Vivo* Post Oral Administration of a Single Dose of INH and Prodrugs (**1a**–**1j**) in Mice^b^ ^,^ ^c^

compound (molar weight)	actual dose (mg/kg)	mole eq. of INH compared to 10 mg/kg of INH	eAUC (INH)	eAUC (AcINH)
INH (137.1)	10	1	1159 ± 679	437 ± 202
**1a** (231.6)	10	0.59	BLQ^d^	BLQ^d^
**1b** (245.7)	10	0.56	234 ± 39[Table-fn t5fn5]	BLQ^d^
**1c** (259.7)	10	0.53	BLQ^d^	BLQ^d^
**1d** (259.7)	10	0.53	2224 ± 354[Table-fn t5fn5]	BLQ^d^
**1e** (273.7)	10	0.5	677 ± 53[Table-fn t5fn7]	470 ± 42[Table-fn t5fn7]
**1f** (273.7)	10	0.5	244 ± 36[Table-fn t5fn5]	176 ± 8[Table-fn t5fn7]
**1g** (257.7)	10	0.53	198 ± 37[Table-fn t5fn5]	163 ± 12[Table-fn t5fn5]
**1h** (307.7)	10	0.44	217 ± 34[Table-fn t5fn5]	166 ± 11[Table-fn t5fn5]
**1i** (273.7)	10	0.57	3101 ± 690[Table-fn t5fn6]	BLQ^d^
**1j** (293.7)	10	0.53	157 ± 9.4[Table-fn t5fn5]	BLQ^d^

a Blood samples were withdrawn at *t* = 20 and 120 m.

b Balb/C mice (*n* = 3, age: 7–9 weeks) were administered with a dose of 10 mg/kg

c Data are expressed as the mean ± SD (*n* = 3), with statistics quantified using one-way ANOVA with Dunnett’s multiple comparison test, with the naïve INH group serving as the reference.

d Concentration was below limit of quantification at all the time points.

* *p* ≤ 0.05

**** *p* ≤ 0.0001.

ns *p* is nonsignificant.

### *In Vivo* Plasma Exposure Analysis after a Single Oral Dose Administration of INH and Prodrugs **1a**–**1j** to Mice

The *in vivo* plasma exposure of released INH and its metabolite AcINH following a single oral dose of compounds **1a**–**1j** was evaluated using the literature-reported RACE-PK (Rapid Assessment of Chemical Exposure–Pharmacokinetic) protocols.^19^ Briefly, each compound was administered orally at a dose of 10 mg/kg to mice (*n* = 3), and blood samples were collected at two time points: 20 and 120 min postdose. Quantification of released INH and AcINH was performed using the previously described LC-MS method. All prodrugs were found to be labile, releasing INH *in vivo*. Plasma concentrations of released INH and AcINH were determined at 20 min postadministration, and a comparative concentration analysis was performed (Figure 2A–B). Prodrugs **1d** and **1i** demonstrated significantly higher plasma concentrations of released INH (2074 and 3294 ng/mL, respectively) compared to the naïve INH dosing group (1004 ng/mL) at the 20 min time point (Figure 2A). As expected, the plasma levels of AcINH in the dosing groups of prodrugs **1a**–**1j** were significantly lower than those in the naïve INH group (Figure 2B). The released INH from prodrugs **1a** and **1c** and the released AcINH from prodrugs **1a**, **1b**, **1c**, **1d**, **1i**, and **1j** were found to be below the limit of quantification (BLQ). The eAUC values (representing total plasma exposure over the 20–120 min period) for released INH showed a 1.9-fold and 2.7-fold increase following the oral administration of prodrugs **1d** and **1i**, respectively, compared to the exposure observed with naïve INH. In contrast, the plasma exposure of AcINH formed *in vivo* from **1d** and **1i** remained at BLQ levels (Table 5). These findings suggest that the appropriate labile derivatization of INH can significantly influence its *in vivo* metabolic transformation. This provides proof-of-concept data supporting the proposed hypothesis.

### *In Vivo* Plasma Exposure Analysis after Repetitive Single Oral Dosing of INH and Prodrugs (**1a**–**1h**) for 10 Consecutive Days

Repeat-dose analysis over multiple days provides critical insights into the exposure, induced metabolism, and accumulation of a drug molecule over an extended period, which is essential for drug leads intended for chronic dosing regimens. Prodrugs **1a**–**1h** were evaluated for the plasma exposure levels of *in vivo* released INH, AcINH, INA, and AcHz following the first and 10th doses in a 10-day repeat-dose plasma exposure study. All animals received a single daily dose for ten consecutive days, with INH administered at 10 mg/kg and prodrugs **1a**–**1h** at a molar equivalent dose of 10 mg/kg of INH. All animals remained healthy throughout the study, as indicated by stable feed intake and body weight. (see Table S26 for **1d** and INH groups). All prodrugs underwent hydrolysis to release INH *in vivo*, confirming their bioreversible nature (Figure 3). Oral administration of prodrug **1d** resulted in a 2-fold and 1.5-fold increase in plasma concentration of INH (3333 and 4310 ng/mL) at 20 min postdosing on days 1 and 10, respectively, compared to corresponding levels of naïve INH (1675 and 2793 ng/mL). All compounds showed a statistically significant decrease in AcINH formation *in vivo*. Notably, compound **1d** led to a 7.9-fold decrease in plasma AcINH concentration at 20 min postdosing on day 1 (87.6 ng/mL for **1d** versus 691.3 ng/mL for INH) and a 15.7-fold decrease on day 10 (78.4 ng/mL for **1d** versus 1234 ng/mL for INH). Additionally, prodrugs **1a**, **1b**, and **1g** demonstrated 9.5-fold, 9-fold, and 9.8-fold decrease, respectively, in plasma AcINH concentrations at 20 min postdosing on day 10 (**1a**: 129.6 ng/mL; **1b**: 136.0 ng/mL; **1g**: 124.6 ng/mL; compared to naïve INH: 1234 ng/mL). Compound **1d** exhibited a significant increase in the total systemic exposure (eAUC) of released INH compared to naïve INH over the 6 h time interval, as observed on both day 1 and day 10 (Tables [Table tbl6A] and 6b). Plasma exposure levels of AcINH formed *in vivo* from all prodrugs remained consistently lower at both time points compared to naïve INH dosing (Tables [Table tbl6A] and 6b), aligning with the single-dose plasma exposure results (Table 5). The plasma exposure of two other undesired metabolites, INA (on days 1 and 10) and AcHz (on day 1), was comparable to that of naïve INH. Notably, AcHz levels on day 10 were comparable to BLQ levels. On day 10, prodrug **1d** demonstrated a 4.3-fold decrease in AcINH exposure. These results confirm that the labile chemical derivatization of INH, achieved by masking the free −NH_2_ group, effectively reduces *in vivo* metabolism and enhances the plasma exposure of released INH.

**6A tbl6A:** Plasma eAUC_20m–6h_ [Table-fn tbl6Afn1] of Free INH, AcINH, INA, and AcHz Formed *In Vivo* Post Oral Administration of 1st Dose in Multiple-Dose Plasma Exposure Analysis[Table-fn tbl6Afn2] ^,^ [Table-fn tbl6Afn3]

compound	dose (mg/kg)	eAUC (INH)	eAUC (AcINH)	eAUC (INA)	eAUC (AcHz)
INH	10.00	2692 ± 328	2044 ± 139	1171 ± 124	26,274 ± 9745
**1a**	16.89	1492 ± 6[Table-fn tbl6Afn8]	799 ± 132[Table-fn tbl6Afn8]	707 ± 47[Table-fn tbl6Afn9]	28,826 ± 7604[Table-fn tbl6Afn9]
**1b**	17.92	963 ± 196[Table-fn tbl6Afn8]	670 ± 78[Table-fn tbl6Afn8]	1130 ± 345[Table-fn tbl6Afn9]	25,425 ± 8400[Table-fn tbl6Afn9]
**1c**	18.94	1008 ± 280[Table-fn tbl6Afn8]	774 ± 35[Table-fn tbl6Afn8]	1237 ± 183[Table-fn tbl6Afn9]	22,495 ± 1593[Table-fn tbl6Afn9]
**1d**	18.94	4479 ± 42[Table-fn tbl6Afn8]	509 ± 28[Table-fn tbl6Afn8]	909 ± 23[Table-fn tbl6Afn9]	23,944 ± 6050[Table-fn tbl6Afn9]
**1e**	19.96	1179 ± 61[Table-fn tbl6Afn8]	935 ± 43[Table-fn tbl6Afn8]	1169 ± 118[Table-fn tbl6Afn9]	23,773 ± 1037[Table-fn tbl6Afn9]
**1f**	19.96	1126 ± 177[Table-fn tbl6Afn8]	1095 ± 68[Table-fn tbl6Afn8]	1934 ± 387[Table-fn tbl6Afn6]	22,318 ± 5156[Table-fn tbl6Afn9]
**1g**	18.79	851 ± 196[Table-fn tbl6Afn8]	769 ± 61[Table-fn tbl6Afn8]	2645 ± 308[Table-fn tbl6Afn8]	18,232 ± 4378[Table-fn tbl6Afn9]
**1h**	22.44	1552 ± 83[Table-fn tbl6Afn8]	1349 ± 119[Table-fn tbl6Afn8]	1038 ± 67[Table-fn tbl6Afn9]	21,486 ± 2000[Table-fn tbl6Afn9]

a Blood samples were withdrawn at *t* = 20 m, 120 m, and 6 h.

b Balb/C mice (*n* = 3, age: 12–14 weeks) were administered with INH at dose of 10 mg/kg and prodrugs at a molar equivalent dose of 10 mg/kg of INH.

c Data are expressed as the mean ± SD (*n* = 3), with statistics quantified using one-way ANOVA with Dunnett’s multiple comparison test, with the naïve INH group serving as the reference.

** *p* ≤ 0.01.

**** *p* ≤ 0.0001.

ns *p* is nonsignificant.

**6B tbl6b:** Plasma eAUC_20m–6h_ [Table-fn tbl6bfn1] of Free INH, AcINH, INA, and AcHz Formed *In Vivo* Post Oral Administration of 10th Dose in Multiple Dose Plasma Exposure Analysis[Table-fn tbl6bfn2] ^,^ [Table-fn tbl6bfn3]

compound	dose (mg/kg)	eAUC (INH)	eAUC (AcINH)	eAUC (INA)	eAUC (AcHz)
INH	10.00	4286 ± 595	2645 ± 150	750 ± 133	BLQ[Table-fn tbl6bfn4]
**1a**	16.89	633 ± 324[Table-fn tbl6bfn8]	554 ± 58[Table-fn tbl6bfn7]	831 ± 39[Table-fn tbl6bfn9]	BLQ[Table-fn tbl6bfn4]
**1b**	17.92	1196 ± 115[Table-fn tbl6bfn8]	661 ± 85[Table-fn tbl6bfn8]	1420 ± 97[Table-fn tbl6bfn9]	BLQ[Table-fn tbl6bfn4]
**1c**	18.94	1009 ± 27[Table-fn tbl6bfn8]	908 ± 47[Table-fn tbl6bfn8]	1809 ± 225[Table-fn t5fn5]	BLQ[Table-fn tbl6bfn4]
**1d**	18.94	6170 ± 2096[Table-fn t5fn5]	607 ± 64[Table-fn tbl6bfn8]	1219 ± 320[Table-fn tbl6bfn9]	BLQ[Table-fn tbl6bfn4]
**1e**	19.96	1461 ± 143[Table-fn tbl6bfn7]	1120 ± 53[Table-fn tbl6bfn8]	1809 ± 917[Table-fn t5fn5]	BLQ[Table-fn tbl6bfn4]
**1f**	19.96	920 ± 9[Table-fn tbl6bfn8]	883 ± 67[Table-fn tbl6bfn8]	1406 ± 201[Table-fn tbl6bfn9]	BLQ[Table-fn tbl6bfn4]
**1g**	18.79	670 ± 22[Table-fn tbl6bfn8]	657 ± 42[Table-fn tbl6bfn8]	1867 ± 109[Table-fn t5fn5]	BLQ[Table-fn tbl6bfn4]
**1h**	22.44	1625 ± 440[Table-fn tbl6bfn7]	1187 ± 205[Table-fn tbl6bfn8]	1024 ± 436[Table-fn tbl6bfn9]	BLQ[Table-fn tbl6bfn4]

a Blood samples were withdrawn at *t* = 20 m, 120 m, and 6 h.

b Balb/C mice (*n* = 3, age: 12–14 weeks) were administered with INH at dose of 10 mg/kg and prodrugs at a molar equivalent dose of 10 mg/kg of INH.

c Data are expressed as the mean ± SD (*n* = 3), with statistics quantified using one-way ANOVA with Dunnett’s multiple comparison test, with the naïve INH group serving as the reference.

d Concentrations were BLQ at all the time points.

*** *p* ≤ 0.001.

**** *p* ≤ 0.0001.

ns *p* is nonsignificant.

**Figure 3:**
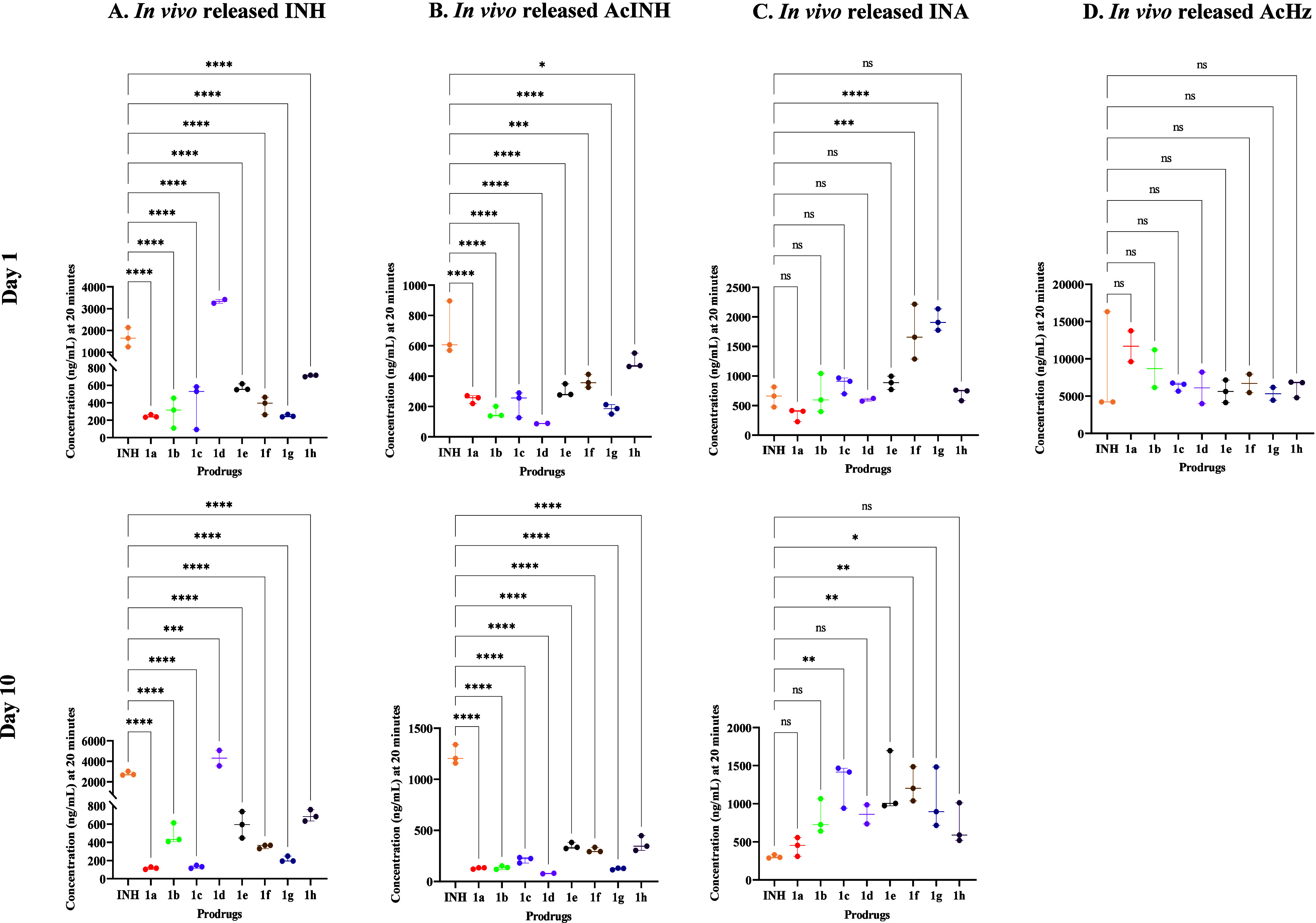
Plasma concentration of *in* vivo released free INH (A), AcINH (B), INA (C), and AcHz (D) at 20 min post oral administration of 1st and 10th dose of INH at 10 mg/kg and prodrugs **1a**–**1h** at molar equivalent dose of 10 mg/kg of INH to Balb/C mice (*n* = 3) via oral gavage in 10-day repetitive single oral dose plasma exposure analysis. AcHz levels were BLQ at a 10th dose analysis. Data are expressed as the mean ± SD, with statistics quantified using one-way ANOVA with Dunnett’s multiple comparison test. Note: *p ≤ 0.05; **p ≤ 0.01; ***p ≤ 0.001; ****p ≤ 0.0001; ns, *p* is nonsignificant.

### Comparative Plasma Exposure Analysis in Mice Following the Oral Administration of a Single Dose of **1d** and INH at Three Incremental Dose Levels

The plasma exposure of INH and its metabolites were analyzed following oral dosing of the lead **1d** compared to naïve INH at three incremental oral doses: 1, 3, and 10 mg/kg. It is important to mention that all the dose concentrations of **1d** in this experiment represent 0.53 mol equivalents loading of INH compared to naïve INH doses, considering the molecular weight of **1d** and INH. The plasma concentrations of *in vivo*-released INH and INA at 20 min postdosing for both **1d** and naïve INH increased proportionally with the administered dose (Figure 4). Notably, at all dosing concentrations, the plasma levels of AcINH in mice dosed with **1d** were found to be lower than the AcINH levels in mice dosed with naïve INH at a 20 min time point. The total systemic exposure (eAUC) of INH was also found to be higher in **1d** dosed groups compared to naïve INH at all dosing concentrations, even at a half molar equivalent dose (Table 7). In this experiment, the eAUC of the unchanged **1d** was also quantified along with INH. There was a significant amount of unchanged **1d** in systemic circulation at all three doses. The data of Table 7 reveal distinct pharmacokinetic patterns between prodrug **1d** and free INH across incremental dose ranges. At all the dosing concentrations, there was a significant systemic exposure of unchanged **1d**, which showed a dosing concentration dependent increase. The total systemic exposure of released INH also showed dose linearity in **1d** as well as naïve INH dosing group. Importantly, in all three dosing concentrations of **1d**, there was a significantly higher eAUC of released INH compared to that of naive INH dosing. These findings highlight the potential of **1d** as an efficient prodrug of INH that provides a significantly higher systemic exposure of the free INH.

**Figure 4:**
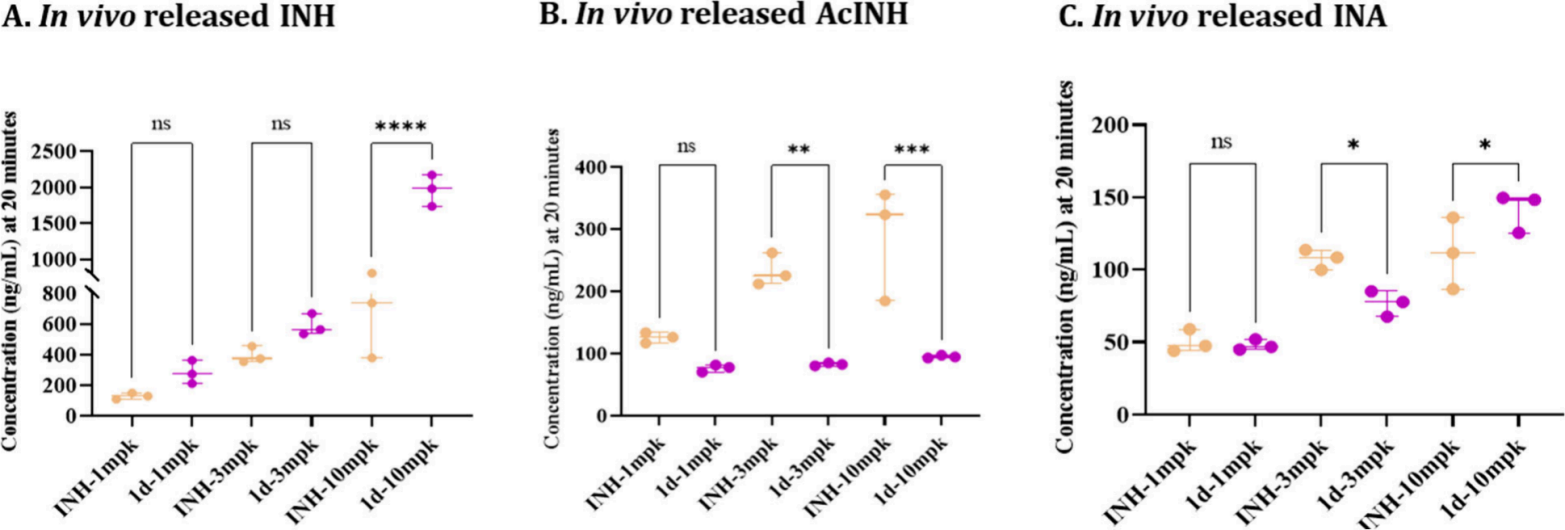
Plasma concentration of *in vivo* released free INH (A), AcINH (B), and INA (C) at 20 min after oral administration of single dose of INH and **1d** at three incremental doses (1, 3, and 10 mg/kg) to Balb/C mice (*n* = 3) via oral gavage. Data are expressed as the mean ± SD, with statistics quantified using one-way ANOVA with Dunnett’s multiple comparison test. Note: *p ≤ 0.05; **p ≤ 0.01; ***p ≤ 0.001; ****p ≤ 0.0001; ns, *p* is nonsignificant.

**Table 7:** Plasma eAUC_20m–6h_ ^a^ of Prodrug **1d**, Free INH, AcINH, and INA Post Oral Administration of Single Dose of INH and **1d** at Three Incremental Doses^b^ ^,^ ^c^ ^,^ ^d^ ^,^ ^e^

compound	dose (mg/kg)	mol. eq. of INH	eAUC (unchanged **1d**)	eAUC (INH)
INH	1	1	NA	21 ± 90^f^
**1d**	1	0.53	2916 ± 1005	624 ± 90[Table-fn t7fn9]
INH	3	1	NA	690 ± 88
**1d**	3	0.53	5799 ± 858	927 ± 82[Table-fn t7fn7]
INH	10	1	NA	1115 ± 207
**1d**	10	0.53	20,138 ± 2821	2580 ± 351[Table-fn t7fn8]

a Blood samples were withdrawn at *t* = 20 m, 120 m, and 6 h.

b Balb/C mice (*n* = 3, age: 6–7 weeks) were administered with INH and 1d at dose of 1, 3, and 10 mg/kg.

c Data are expressed as the mean ± SD (*n* = 3), with statistics quantified using one-way ANOVA with Dunnett’s multiple comparison test.

d AcHz formed *in vivo* was reported as BLQ.

e In ng·h/mL.

f eAUC calculated from 0 to 0.083 h, since INH was detected only at *t* = 0.083 h and found to be BLQ at other time points. NA, not applicable.

* *p* ≤ 0.05.

** *p* ≤ 0.01.

**** *p* ≤ 0.0001.

### Complete Pharmacokinetic Analysis after Oral Administration of a Single Dose of INH and Selected Prodrugs (**1d**, **1a**, and **1h**) in Mice

A comprehensive pharmacokinetic (PK) analysis was conducted on three selected prodrugs, including the lead compound **1d** and naive INH, in mice (*n* = 12 per compound) to validate the trends observed in single-dose plasma exposure studies. This study evaluated PK parameters for the unchanged prodrugs, released INH, and its metabolites by measuring their respective plasma levels at multiple time points over a 24 h period following oral gavage of prodrug **1a**, **1d**, and **1h** and naïve INH as control. Prodrug **1d** was selected based on its higher plasma exposure of released INH observed in previous studies (Tables 5, [Table tbl6A], and 6b). The prodrugs **1a** and **1h** were included to validate single-dose plasma exposure studies and evaluate the impact of structural variations compared to **1d**. Naive INH was administered at a dose of 10 mg/kg, and the prodrugs (**1a**, **1d**, and **1h**) were dosed at a molar equivalent to 10 mg/kg of INH. Consistent with earlier findings, the total systemic exposure (AUC) of *in vivo*-released INH from prodrug **1d** exceeded that of naive INH and the other prodrugs tested (Figure 5 and Table 8). Naive INH displayed a rapid absorption profile, with a *C*_max_ of 2559 ng/mL at *T*_max_ = 0.08 h and a significant systemic exposure (AUC = 2716 ng·h/mL). It was efficiently eliminated, with a half-life (*t*_1/2_) of 0.68 h and a clearance rate (Cl) of 113750 mL/kg·h, consistent with its known PK profile.^20^

**Figure 5:**
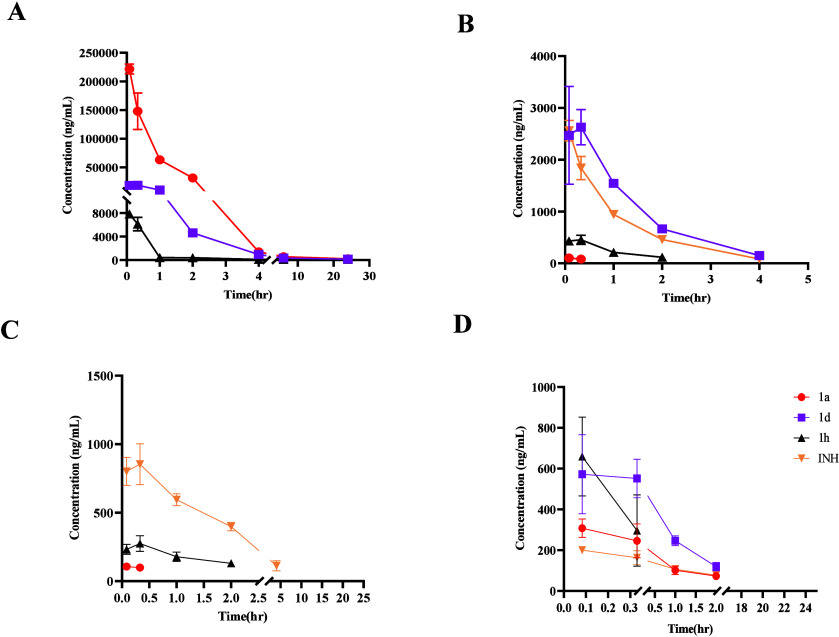
Pharmacokinetic profile of prodrugs **1a**, **1d**, and **1h** (A), *in vivo* released INH (B), AcINH (C), and INA (D) after oral administration of **INH** at 10 mg/kg and **1a**, **1d**, and **1h** at molar equivalent dose of 10 mg/kg of INH to Balb/C mice (*n* = 12) via oral gavage. Data are expressed as the mean ± SD.

**Table 8:** Pharmacokinetic Parameters of Prodrugs (**1a**, **1d**, **1h**), Free INH, AcINH, and INA Formed *In Vivo* Post Oral Administration of a Single Dose of INH, **1a**, **1d**, and **1h** ^a^ ^,^ ^b^

	Unchanged Prodrug (**1a**, **1d**, **1h**)					
dosed compound	*t*_1/2_ ^c^	*C*_max_ ^d^	*T*_max_ ^c^	AUC_5m‑24 h_ ^e^	V_d_ ^f^	Cl^g^
**1a**	0.46	221,707 ± 12,083	0.08	205,519 ± 17,382	77 ± 4	133 ± 56
**1d**	1.04	18,690 ± 1881	0.33	33,216 ± 1119	1121 ± 345	1279 ± 731
**1h**	0.34	7819 ± 755	0.08	8879 ± 2871	2899 ± 300	6088 ± 1081

	Released INH					
dosed compound	*t*_1/2_ ^c^	*C*_max_ ^d^	*T*_max_ ^c^	AUC_5m–24h_ ^e^	*V*_d_ ^f^	Cl^g^
INH	0.68	2559 ± 281	0.08	2716 ± 198	3958 ± 461	113,750 ± 7898
**1a**	ND[Table-fn t8fn9]	102 ± 7	0.08	23 ± 3	98,831 ± 6271	ND^f^
**1d**	0.88	2628 ± 482	0.33	3948 ± 453	6837 ± 5349	1700 ± 1241
**1h**	2.28	453 ± 124	0.33	495 ± 76	24,116 ± 4318	18,969 ± 6294

	Released AcINH					
dosed compound	*t*_1/2_ ^c^	*C*_max_ ^d^	*T*_max_ ^c^	AUC_5m–24h_ ^e^	*V*_d_ ^f^	Cl^g^
INH	3.14	854 ± 122	0.33	1698 ± 101	NA	NA
**1a**	ND[Table-fn t8fn9]	107 ± 2	0.08	26 ± 1	NA	NA
**1d**	BLQ[Table-fn t8fn10]	BLQ[Table-fn t8fn10]	BLQ[Table-fn t8fn10]	BLQ[Table-fn t8fn10]	NA	NA
**1h**	1.943	275 ± 47	0.33	369 ± 33	NA	NA

	Released INA					
dosed compound	*t*_1/2_ ^c^	*C*_max_ ^d^	*T*_max_ ^c^	AUC_5m–24h_ ^e^	*V*_d_ ^f^	Cl^g^
INH	0.64	200 ± 14	0.08	227 ± 16	NA	NA
**1a**	0.31	308 ± 37	0.08	272 ± 49	NA	NA
**1d**	1.93	573 ± 159	0.08	589 ± 60	NA	NA
**1h**	0.19	659 ± 158	0.08	289 ± 71	NA	NA

a Balb/C mice (*n* = 12, age: 8–9 weeks) were administered with INH at a dose of 10 mg/kg and prodrugs **1a**, **1d**, and **1h** at a dose of a molar equivalent dose of 10 mg/kg of INH. NA, not applicable; ND, not determined.

b AcHz formed *in vivo* was found to be BLQ in all samples.

c In hr.

d In ng/mL.

e In ng·h/mL.

f In mL/kg.

g In mL/kg·h.

h Not Determined since levels were BLQ for most of the data points.

I Concentrations were BLQ at all the time points.

The primary acetylated metabolite, AcINH, exhibited a *C*_max_ of 854 ng/mL, a longer *t*_1/2_ of 3.14 h, and an AUC of 1698 ng·h/mL, reflecting slower clearance. INA formation was limited, with a *C*_max_ of 200 ng/mL and an AUC of 227 ng·h/mL, while AcHz levels were below the limit of quantification (BLQ). These findings confirm that INH undergoes a primary metabolism to AcINH with minimal INA formation. Prodrug **1a** demonstrated exceptionally high systemic exposure of the unchanged parent prodrug **1a**, with a *C*_max_ of 221,707 ng/mL at *T*_max_ = 0.08 h and an AUC of 205,519 ng·h/mL, indicating rapid absorption but poor bioconversion to free INH. This was evident with the limited release of INH (AUC = 23 ng·h/mL) and AcINH (AUC = 26 ng·h/mL) in the **1a** dosing group. Conversely, INA showed modest systemic exposure (AUC = 272 ng·h/mL, *t*_1/2_ = 0.31 h). These results indicate that prodrug **1a** is poorly suited for the *in vivo* delivery of INH. Prodrug **1h** showed limited systemic exposure of the unchanged prodrug (AUC = 8879 ng·h/mL, *t*_1/2_ = 0.34 h, Cl = 6088 mL/kg·h). The INH released from **1h** achieved a *C*_max_ of 453 ng/mL and an AUC of 495 ng·h/mL, indicating moderate efficiency *in vivo*, surpassing **1a** but underperforming compared to naive INH. The systemic exposure of AcINH (AUC = 369 ng·h/mL) and INA (AUC = 289 ng·h/mL) was notable but still lower than the concentration of AcINH in naïve INH dosing group. However, prodrug **1d** exhibited a better PK profile, characterized by efficient *in vivo* release of free INH and minimal metabolite formation. Released INH from **1d** was found to have an AUC of 3948 ng·h/mL, the highest among all compounds evaluated. Significant systemic exposure of INA (*C*_max_ = 573 ng/mL, AUC = 589 ng·h/mL) suggested efficient metabolism of **1d** to INA. Notably, AcINH levels and AcHz levels were BLQ, indicating minimal acetylation of released INH. These findings confirm that prodrug **1d** efficiently delivers INH with favorable release kinetics and enhanced systemic exposure while minimizing the formation of undesired metabolites such as AcINH and AcHz. The complete PK analysis concluded that oral administration of prodrug **1d** resulted in a 1.5-fold increase in plasma exposure of free INH (AUC = 3948 ng·h/mL) compared to naive INH (AUC = 2716 ng·h/mL), with comparable *C*_max_. Additionally, released INH from **1d** demonstrated an extended *T*_max_ and a notable 1.3-fold increase in *t*_1/2_. **1d** also exhibited a significant systemic exposure of the unchanged **1d** (*C*_max_ = 18,690 ng/mL, *T*_max_ = 0.33 h, AUC = 33,216 ng·h/mL) just like **1a** and **1h**. We also attempted to quantify the released Hz in INH and **1d** dosing groups using a reported derivatization-based method (Figure S39).^12^ However, the amount of Hz was found to be BLQ in both INH and **1d** dosing groups (Table S34). Cumulatively, these results support the hypothesis that structural modification of INH with an *in vivo* labile moiety can favorably alter its pharmacokinetic profile, enhancing systemic exposure and reducing undesirable metabolism.

### Comparative Pharmacokinetic Analysis after Intravenous Administration of a Single Dose of **1d** and INH in Mice

We assessed the pharmacokinetic (PK) parameters of *in vivo-*released INH, AcINH, and INA following intravenous (IV) administration of prodrug **1d** and naive INH at a molar equivalent dose of 1 mg/kg of INH (Table 9 and Figure S47). After IV administration, the naive INH and **1d** dosing groups exhibited comparable PK profiles for *in vivo*-released INH, with matching *C*_max_, *t*_1/2_, AUC, and clearance (Cl) values. Notably, the dosing group for **1d** exhibited systemic exposure to the unchanged form of **1d** (AUC = 18 ng·h/mL) with an exceptionally high clearance rate (Cl = 106,642 mL/kg·h). Importantly, although the naive INH dosing group showed detectable systemic exposure to AcINH, no AcINH was observed in the metabolite analysis following **1d** administration. However, unlike the naive INH dosing group, plasma exposure of INA was observed only for the first time point in the **1d** dosing group. These findings suggest that **1d** undergoes limited and altered metabolism compared with naive INH.

**Table 9:** Pharmacokinetic Parameters of Prodrug **1d**, Free INH, AcINH, and INA Formed *In Vivo* Post Intravenous Administration of a Single Dose of INH and **1d** ^a^ ^,^ ^b^

	Unchanged **1d**					
dosed compound	*t*_1/2_ ^c^	*C*_max_ ^d^	*T*_max_ ^c^	AUC_5m–24h_ ^e^	*V*_d_ ^f^	Cl^g^
**1d**	ND^h^	90 ± 5	0.08	18 ± 1	21,024 ± 1190	106,642 ± 3838

	Released INH					
dosed compound	*t*_1/2_ ^c^	*C*_max_ ^d^	*T*_max_ ^c^	AUC_5m–24h_ ^e^	*V*_d_ ^f^	Cl^g^
**INH**	0.31	51 ± 3	0.17	759 ± 63	20,717 ± 253	1326 ± 106
**1d**	0.34	55 ± 4	0.08	758 ± 41	18,421 ± 1370	1323 ± 74

	Released AcINH					
dosed compound	*t*_1/2_ ^c^	*C*_max_ ^d^	*T*_max_ ^c^	AUC_5m–24h_ ^e^	*V*_d_ ^f^	Cl^g^
**INH**	3.02	44 ± 2	0.33	37 ± 2	NA	NA
**1d**	BLQ^i^	BLQ^i^	BLQ^i^	BLQ^i^	NA	NA

	Released INA					
dosed compound	*t*_1/2_ ^c^	*C*_max_ ^d^	*T*_max_ ^c^	AUC_5m–24h_ ^e^	*V*_d_ ^f^	Cl^g^
**INH**	BLQ^i^	BLQ^i^	BLQ^i^	BLQ^i^	NA	NA
**1d**	ND^h^	ND^h^	ND^h^	ND^h^	NA	NA

a Balb/C mice (*n* = 12, age: 8–9 weeks) were administered with INH at a dose of 1 mg/kg and prodrug **1d** at a dose of a molar equivalent dose of 1 mg/kg of INH. NA, not applicable; ND, not determined.

b AcHz formed *in vivo* was found to be BLQ in all samples.

c In hr.

d In ng/mL.

e In ng·h/mL.

f In mL/kg.

g In mL/kg·h.

h Cannot be determined since levels were BLQ for most of the data points.

i Concentration were BLQ at all the time points.

### Intestinal and Stomach Homogenate Conversion Assay of INH, **1a**, **1d**, and **1h**

The bioconversion of prodrugs **1a**, **1d**, and **1h** was evaluated using mice intestinal and stomach tissue homogenates with INH as a control. The percentage remaining of the parent compound and total exposure of released INH and metabolites were determined after incubation with the respective tissue homogenate. The low molecular weight prodrugs **1a** and **1d** demonstrated higher bioconversion in mice stomach homogenates. On the contrary, prodrug **1h** showed higher bioconversion in intestine homogenates (Table 10). The prodrug **1d** provided a better release of INH than naïve INH or the other two prodrugs, in both stomach and intestine tissue homogenates. All three prodrugs significantly restricted the total exposure of INA as a metabolite in both the tissue homogenates compared to naïve INH.

**Table 10:** Mouse Stomach and Intestine Homogenates Conversion and Estimated Total Exposure (eAUC_0–20h_) of the **1a**, **1d**, **1h**, Released Free INH, AcINH, and INA after 20 h *Ex Vivo* Incubation of the Respective Prodrug at 50 μg/mL Concentration^a^

		precentage of prodrug remaining after 20 h of incubation		eAUC^b^ in stomach homogenate				eAUC^b^ in intestinal homogenate			
compound	mol. eq of INH	stomach homogenate	intestine homogenate	prodrug	INH	AcINH	INA	prodrug	INH	AcINH	INA
INH	NA	NA	NA	NA	118,433	6583	15,336	NA	195,148	9661	23,906
**1a**	0.59	1	85	412,453	175,756	7556	11,174	908,764	20,338	9383	9934
**1d**	0.53	3	87	433,081	205,246	7473	12,302	728,889	339,286	8931	8851
**1h**	0.53	29	43	691,560	10,448	6449	9716	761,596	15,566	6741	12,696

a AcHz formed was found to be BLQ.

b In ng·h/mL; NA, not applicable.

### Mouse Liver Homogenate Conversion Assay and Human Liver Microsomal Stability Analysis of INH and **1d**

The bioconversion of prodrug **1d** was assessed using mouse liver tissue homogenate with INH serving as a control. Following a 2 h incubation, the percentage of parent compound remaining and the total exposure of released INH and its metabolites were quantified. Only 27% of the prodrug **1d** was found remaining after 2 hours of incubation. Prodrug **1d** demonstrated a 2-fold, 7-fold, and 2.1-fold increase in the cumulative exposure of INH, AcINH, and INA, respectively, compared to the INH control group (Table 11). However, isolated human liver microsomal stability analysis revealed that prodrug **1d** exhibited slightly high metabolic stability along with a significant exposure of free INH.

**Table 11:** Mouse Liver Tissue Homogenate Conversion and Human Liver Microsomal Stability Analysis of the Prodrug **1d** and INH after 2 h *Ex Vivo* Incubation^a^

		mouse liver homogenate					human liver microsomes		
			eAUC_5–120m_ ^b^					eAUC_0–120m_ ^b^	
compound	mol. eq. of INH	precentage of **1d** remaining after 2 h of incubation	**1d**	INH	AcINH	INA	percentage of **1d**/INH remaining after 2 h of incubation	**1d**	INH
INH	NA	NA	NA	4,017,424	6161	5869	66	NA	19,332
**1d**	0.53	27	261,525	8,306,610	43,180	12,377	77	80,176	28,416

a AcHz formed was found to be BLQ.

b In ng·h/mL; NA, not applicable.

### Evaluation of Serum Alanine Transaminase (ALT) and Aspartate Transaminase (AST) for Liver Injury

To evaluate the impact of **1d** on liver injury markers (ALT/AST), a repeat-dose oral study was conducted using higher doses of INH and **1d**. INH was administered at 90 mg/kg, a dose known from the literature^21^ to induce liver injury in mice. Given that **1d** produces 1.45-fold higher INH AUC compared to native INH, its dose was adjusted accordingly and set at 117.5 mg/kg (see Supporting Information). Serum ALT and AST levels in the **1d**-treated group were significantly lower than those in the INH group and were comparable to control levels (Figure S48). INH is associated with peripheral neuropathy through the depletion of vitamin B6. To investigate this, we measured levels of pyridoxal and pyridoxal-5′-phosphate, from the liver tissue samples. The concentrations of both metabolites were found comparable between the INH and **1d** treatment groups (Figure S49).

## Discussion

A comprehensive review of INH’s *in vivo* metabolic transformations highlights the critical role of the terminal −NH_2_ group due to its high reactivity. To address these challenges, a labile prodrug approach was proposed as a strategy to restrict these adverse reactions.^3^ There are numerous successful examples where similar metabolic issues in other drugs were mitigated by chemically modifying reactive functional groups into labile prodrugs.^8,9^ However, to our understanding, no similar effort is reported for INH. The literature review highlights that a codrug strategy using INH conjugates has been explored to mitigate INH-induced hepatotoxicity, which is different from the present prodrug approach. These codrug studies largely focus on combining INH with antioxidant agents (e.g., cinnamic acid derivatives,^22^ phenolic acids,^23^ aminothiols,^24^ and α-lipoic acid).^25^ The reported conjugates showed varying degrees of hepatoprotective effects, primarily through antioxidant mechanisms owing to coeluting pharmacologically active moiety. However, significant gaps remain, as most studies did not investigate INH’s *in vivo* release kinetics, metabolism, or pharmacokinetics after conjugation. Controlled-release formulations, such as polyaspartic acid, polysuccinimide,^26^ or polyethylene glycol (PEG),^27^ have also been proposed for prolonged INH exposure and reduced toxicity. While these approaches show promise, further investigation is needed to fully understand these conjugates’ metabolic and pharmacokinetic implications. With this understanding, we designed and synthesized a series of carbamate-based prodrugs of INH, masking its terminal −NH_2_ group, to mitigate undesired metabolic transformations and toxicity. The prodrugs (**1a**–**1j**) were synthesized using a straightforward chemical method by reacting INH with corresponding alkyl chloroformates in a THF–water mixture without requiring an external base. This method provided the corresponding carbamate prodrugs in good to moderate yields. The simple low molecular weight alkyl substitutions were preferred to avoid any pharmacological activity or metabolism associated with the linker moieties. These structural modifications significantly altered the physicochemical properties (Table 1). The changes were particularly pronounced in prodrugs with longer carbon chains or higher molecular weight moieties. All prodrugs exhibited reduced aqueous solubility compared to free INH, due to the impact of these structural modifications on lipophilicity (Table 1). Chemical stability analyses revealed that all synthesized prodrugs were stable in neat DMSO and alkaline buffers except **1j**. However, partial hydrolysis of prodrugs **1b**, **1c**, and **1d** was observed under acidic aqueous conditions, suggesting their potential for hydrolysis in the gastric environment. To explore this further, selected prodrugs were incubated with stomach, intestinal, and liver tissue homogenates from mice and found to be labile. Bioconversion studies conducted in mice and human plasma confirmed that all prodrugs were biologically labile. These findings demonstrate that modifying the terminal −NH_2_ group of INH via a carbamate linkage with low molecular weight moieties successfully generated prodrugs that exhibit chemical stability and desired lability.

The primary objective of this study was to evaluate how biologically labile derivatization of INH, specifically targeting its terminal −NH_2_ group, influences systemic exposure of INH and its major metabolites (AcINH, INA, and AcHz) following oral and intravenous administration in healthy mice. AcINH and AcHz are two key undesired and potentially toxic metabolites of INH, formed *in vivo* via the enzymatic action of NAT-2 and amidases in the gut, plasma, and liver. Among these, plasma exposure of AcINH is a critical marker for INH-induced hepatotoxicity.^28^ A novel, derivatization-free LC-MS method was developed to simultaneously quantify the concentrations of INH, AcINH, INA, and AcHz. Initial *in vivo* screening for plasma exposure involved a snapshot pharmacokinetic experiment (Figure 2 and Table 5) in mice (*n* = 3) following single oral doses of the prodrugs, with naïve INH serving as the control. All the prodrugs demonstrated *in vivo* bioconversion, releasing free INH through hydrolytic enzymes in the GI tract, liver, and plasma. This initial screening revealed that all prodrugs significantly altered the plasma concentrations of released INH and AcINH compared to naïve INH. While all the prodrugs restricted the formation of AcINH, prodrugs **1d** (isopropyl carbamate derivative) and **1i** (sec-butyl derivative) showed a remarkable increase in released INH concentration, achieving a 1.9-fold and 2.6-fold improvement in plasma eAUC values for free INH respectively (Table 5, entries **1d** and **1i**). Importantly, the total plasma exposure or eAUC of the notorious metabolite AcINH formed in these dosing groups was found to be below the limit of quantification. These results are particularly noteworthy because the INH equivalent loading in **1d** was only half that of naïve INH, as one mole of **1d** contains 0.53 mol equivalents of INH. The structure–activity relationship within this small set of prodrugs was also intriguing. For example, the difference in eAUC of released INH between **1c** (*n*-propyl; eAUC: BLQ) and **1d** (isopropyl; eAUC 2224 ng·h/mL) suggested a possible role of the sterically hindered carbonyl group of the carbamate moiety as a factor influencing *in vivo* release of free INH from the prodrug. The effect of steric hindrance was also prominent on the INH release from **1i** (sec-butyl; eAUC 3101 ng·h/mL) compared to **1e** (*n*-butyl; eAUC 677 ng·h/mL). The comparative eAUC values of **1e**, **1i**, and **1f** (isobutyl; eAUC 244 ng·h/mL) underscore the impact of alkyl substitution adjacent to the carbonyl group on prodrug’s performance.

The 10-day repeat oral dosing study provided additional insight into the pharmacokinetic profile of free INH and its various metabolites post a single dose daily oral gavage of prodrugs **1a**–**1h** using naïve INH as a control (Figure 3 and Tables [Table tbl6A] and 6b). Plasma exposure (eAUC) of released INH and its metabolites was assessed after the first and 10th doses. Once again, prodrug **1d** outperformed naïve INH by providing significantly increased plasma eAUC of released INH and reduced eAUC of AcINH at both time points. The robust eAUC of released INH at days 1 and 10 in **1d** dosing groups reflects the continuous efficient release of INH, leading to sustained systemic exposure under chronic dosing conditions. There was no significant difference in the eAUC of INA or AcHz between **1d** and naïve INH at either time point, suggesting that **1d** selectively modulates INH and AcINH exposure without affecting other metabolites. All dosing groups, including naïve INH control, showed BLQ levels for AcHz at day 10 compared to day 1, suggesting rapid elimination or inhibition of pathways responsible for AcHz formation. The dose-ranging study in mice provided a comparative plasma exposure of INH, AcINH, and INA following oral administration of **1d** and naïve INH at three incremental doses of 1, 3, and 10 mg/kg (Figure 4 and Table 7). Consistent with previous findings, prodrug **1d** provided higher plasma concentrations of free INH compared to naïve INH across all three doses. The naïve INH and prodrug **1d** both exhibited a dose-dependent in vivo release of INH. Additionally, a significant exposure of unchanged **1d** was found at all three incremental doses in a dose linearity manner which highlighted the efficient gastrointestinal absorption of the **1d**.

Following the encouraging snapshot pharmacokinetic profile and repeat-dose plasma exposure analysis in a small group of mice (*n* = 3), the study was expanded to determine the complete pharmacokinetic profile of the lead compound **1d** compared to naïve INH in a larger cohort of mice (*n* = 12 per group per compound) over a 24 h period. All key pharmacokinetic parameters, i.e., *t*_1/2_, *C*_max_, *T*_max_ and AUC for the circulating prodrug, released INH, AcINH, and INA, were determined. The prodrugs **1a** and **1h** were also included in this study as two structural analogues of **1d** along with the naïve INH group. Similar to previous results, among the three tested analogues, lead **1d** exhibited a 1.45-fold increase in AUC_5m–24h_ and a 1.5-fold increase in half-life (*t*_1/2_) of released INH, compared to naïve INH at molar-equivalent doses. While the *C*_max_ value remained similar, *T*_max_ was extended up to four times that of naïve INH. Additionally, there was a significant decrease in *C*_max_ and AUC of AcINH in the **1d** group compared to the naïve INH dosed group (Table 8 and Figure 5). Unlike AcINH, there was an increase in *C*_max_ and AUC for INA in the **1d** group compared to that in the INH-dosed group. It was intriguing to see a high concentration of unchanged prodrug **1d** circulating in plasma and increased free INH in the **1d** dosing group. Some plasma exposure of unchanged prodrugs was anticipated depending on their differential *in vivo* lability, bioconversion, and oral absorption. However, a high amount of total INH (total sum of free released INH and unchanged parent prodrug) in plasma, especially after equimolar oral gavage of **1d** compared to naïve INH, was surprising. The oral gavage of prodrug **1d** provides a significantly higher concentration of free INH compared to naïve INH and maintains a considerable total exposure of **1d** itself as an unhydrolyzed prodrug in plasma. This can be explained using stomach, intestine, and liver homogenate-based *ex vivo* conversion data (Tables 10 and 11). The *ex vivo* incubation of the naïve INH in mice stomach and intestine tissue homogenates provided a higher concentration of INA and AcINH than prodrug **1d**. This indicates that **1d** prevents significant biotransformation of INH by restricting the hydrolytic and acetylation activity of amidases and NAT-2 in the GI tract of mice. This was also reflected when GI tract and liver metabolism was bypassed using IV dosing of **1d** and naïve INH (Table 9 and Figure S47). The INH released in the **1d** dosing group was found to have AUC, *C*_max_, *t*_1/2_, *V*_d_, and Cl values, matching those of the naïve INH dosing arm. The IV administration of **1d** also showed a very high clearance (Cl 106,642 mL/kg·h) and volume of distribution (*V*_d_ 21,024 mL/kg) for unchanged **1d**, reflecting its limited total plasma exposure (AUC 18 ng·h/mL). Unlike the naïve INH dosing group, the AcINH was BLQ in the **1d** dosing group, suggesting the superiority of the **1d** as a prodrug of INH. Prodrug **1d** also demonstrated a safer profile than INH, and did not alter the levels of serum ALT and AST at higher dose, indicative of reduced sign of hepatotoxicity. The overall data suggest that prodrug **1d** exhibits a distinct pharmacokinetic profile, offering significant advantages over naive INH through efficient active drug release, enhanced systemic retention, and reduced formation and exposure of undesired metabolites. As a next step, it will be important to determine the superiority of **1d** or one of its analogues with a similar or better PK profile using well-designed animal efficacy and detailed safety studies.

## Conclusion

Despite its pivotal role in TB treatment, INH faces significant challenges including severe adverse effects that contribute to poor patient compliance and treatment dropouts. These dropouts are a major factor in the emergence of drug-resistant TB. The dose-related toxicity of INH is primarily linked to its metabolism, driven by the reactive terminal −NH_2_ group. To address these challenges, we proposed the development of plasma-labile derivatives of INH through chemical modification and masking of this terminal −NH_2_ group.^3^ This strategy aims to limit undesired metabolic transformations of INH before it reaches its bacterial target. In this proof-of-concept study, we designed, synthesized, and evaluated a series of carbamate-based prodrugs of INH. *Ex vivo* and *in vivo* investigations were conducted to assess the release of free INH and its various metabolites. This approach and developed prodrugs minimize undesired metabolic degradation and increase systemic exposure of free INH compared to direct dosing of INH. The lead prodrug, **1d**, demonstrated significantly improved systemic exposure to free INH (1.5-fold increase in AUC), a reduced formation of undesired metabolites, and an extended half-life (1.3-fold increase in *t*_1/2_) compared to unmodified INH. The prolonged half-life and elevated plasma concentrations of free INH indicate the potential for less frequent dosing, which could enhance patient adherence to long-duration TB treatment regimens. This prodrug strategy represents a promising translational approach by improving INH’s therapeutic index, offering scope for safer and more effective TB therapy with broader clinical applicability.

## Supplementary Material





## Notes

Experimental procedures; ^1^H and ^13^C NMR spectra; MS spectra; HPLC chromatograms; detailed results of LCMS method validation; time versus plasma concentration data and curves; plasma exposure (eAUC) data of PK studies; and pharmacokinetic profile of prodrug **1d** and INH post intravenous administration (PDF)
Transparent Peer Review report available (PDF)

## Abbreviations

INH: Isoniazid
AcINH: Acetyl Isoniazid
INA: Isonicotinic acid
AcHz: Acetyl Hydrazine
eAUC: Estimated Area Under Curve
MRM: Multiple Reaction Monitoring
DP: Deceleration Potential
CE: Collision Energy
LLOQ: Lower Limit OF Quantification
LQC: Lower Quality Control
MQC: Medium Quality Control
HQC: Higher Quality Control
HLOQ: Highest Level of Quantification
*V*_d_: Volume of Distribution
Cl: Clearence
