# Supplementary material for: Carbamate Prodrugs Restrict In Vivo Metabolism and Improve the Pharmacokinetics of Isoniazid
Source: ACS Cent Sci. 2025 Jul 9;11(8):1467–80. doi: 10.1021/acscentsci.5c00576 (PMC12395308; doi:10.1021/acscentsci.5c00576)
Supplement: Supplementary file 1 [file oc5c00576_si_001.pdf]

Supporting Information

Carbamate Prodrugs Restrict In Vivo Metabolism and Improve the Pharmacokinetics of Isoniazid

Jishnu Sankar[1,2], Manish Kumar Bajpai[1‡], Anjali Chauhan[1‡], Ravi Maddheshiya[1], Nidhi Sharma[1], Aditya Sharma[1], Yashwant Kumar[1], Dinesh Mahajan[1*]

[1]Centre for Drug Discovery, BRIC-Translational Health Science and Technology Institute, Faridabad, Haryana, India, 121001
[2]Manipal Academy of Higher Education, Manipal, India

Corresponding Author
Dinesh Mahajan
E-mail: dinesh.mahajan@thsti.res.in

List of Figure and tables:

Figure S1. ^{1}H NMR and ^{13}C NMR of Prodrug **1a**.

Figure S2. Mass spectra of **1a**

Figure S3. HPLC Chromatogram of **1a**

Figure S4. ^{1}H NMR and ^{13}C NMR of Prodrug **1b**.

Figure S5. Mass spectra of **1b**

Figure S6. HPLC Chromatogram of **1b**

Figure S7. ^{1}H NMR and ^{13}C NMR of Prodrug **1c**.

Figure S8. Mass spectra of **1c**

Figure S9. HPLC Chromatogram of **1c**

Figure S10. ^{1}H NMR and ^{13}C NMR of Prodrug **1d**

Figure S11. Mass spectra of **1d**

Figure S12. HPLC Chromatogram of **1d**

Figure S13. ^{1}H NMR and ^{13}C NMR of Prodrug **1e**.

Figure S14. Mass spectra of **1e**

Figure S15. HPLC Chromatogram of **1e**

Figure S16. ^{1}H NMR and ^{13}C NMR of Prodrug **1f**.

Figure S17. Mass spectra of **1f**

Figure S18. HPLC Chromatogram of **1f**

Figure S19. ^{1}H NMR and ^{13}C NMR of Prodrug **1g**

Figure S20. Mass spectra of **1g**

Figure S21. HPLC Chromatogram of **1g**

Figure S22. ^{1}H NMR and ^{13}C NMR of Prodrug **1h**.

Figure S23. Mass spectra of **1h**

Figure S24. HPLC Chromatogram of **1h**

Figure S25. ^{1}H NMR and ^{13}C NMR of Prodrug **1i**

Figure S26. Mass spectra of **1i**

Figure S27. HPLC Chromatogram of **1i**

Figure S28. ^{1}H NMR and ^{13}C NMR of Prodrug **1j**

Figure S29. Mass spectra of **1j**

Figure S30. HPLC Chromatogram of **1j**

Figure S31. ^{1}H NMR and ^{13}C NMR of Prodrug **AcINH**.

Figure S32. Mass spectra of **AcINH**

Figure S33. Mass spectra of **INH**

Figure S34. Mass spectra of **INA**

Figure S35. Mass spectra of **AcHz**

Figure S36. ^{1}H NMR and ^{13}C NMR of 1,2-bis((E)-4-methylbenzylidene)hydrazine (Derivatized form of hydrazine using p-tolualdehyde)

Figure S37. Mass spectra of 1,2-bis((E)-4-methylbenzylidene)hydrazine (Derivatized form of hydrazine using p-tolualdehyde)

Figure S38. Linearity and trueness of back-calculated results for INH and its metabolites in mouse plasma

Figure S39. Linearity of trueness of back-calculated result for 1,2-bis((E)-4-methylbenzylidene)hydrazine (Derivatized form of hydrazine using p-tolualdehyde)

Figure S40. Time versus plasma concentration profiles of in vivo released INH (A) and AcINH (B), along with the eAUC of in vivo released INH (C) and AcINH (D), following oral administration of INH and prodrugs 1a–1j in a single-dose plasma exposure analysis.

Figure S41. Time versus plasma concentration profile of *in* vivo released INH**(A)**, AcINH**(B)** along with the eAUC of in vivo released INA**(C)** post oral administration of 1st dose of naïve INH and prodrugs **1a-1h** in multiple-dose plasma exposure analysis

Figure S42. eAUC (20m-6hr) of *in vivo* released INH**(A)**, AcINH**(B)**, INA**(C) &** AcHz**(D)** post oral administration of 1st dose of I n multiple dose plasma exposure analysis of INH and prodrugs **1a-1h**

Figure S43. Time versus plasma concentration profile of *in* vivo released INH**(A)**, AcINH**(B)** and INA**(C)** post oral administration of 10th dose of naïve INH and prodrugs **1a-1h** in multiple dose plasma exposure analysis.

Figure S44. eAUC (20m-6hr) of *in vivo* released INH**(A)**, AcINH**(B)** & INA**(C)** post oral administration of 10th dose of in multiple dose plasma exposure analysis of INH and prodrugs **1a-1h.**

Figure S45. Time versus plasma concentration profile of prodrug **1d** and free INH post oral administration of single dose of prodrug **1d** and naïve INH at three incremental doses, 1mg/Kg **(A)**, 3mg/Kg**(B)**, 10mg/Kg**(C)**.

Figure S46. eAUC (20m-6hr) of prodrug **1d** and free INH post oral administration of single dose of prodrug **1d** and naïve INH at three incremental doses, 1mg/Kg **(A)**, 3mg/Kg**(B)**, 10mg/Kg**(C)**.

Figure S47. Pharmacokinetic profile of prodrugs **1d** (A), *in vivo* released INH (B), AcINH (C) and INA (D) after intravenous administration of **INH** at 1mg/kg and prodrug **1d** at molar equivalent dose of 1mg/kg of INH to Balb/C mice (n=12) via oral gavage. Data are expressed as the mean ± SD.

Figure S48. Serum ALT and AST levels after repeat single oral dose daily for 6 consecutive days (blood was drawn post 24hours of 6th dose) in Balb/C mice (n=3/group). Data are expressed as the mean ± SD.

Figure S49. Tissue levels of pyridoxal and pyridoxal-5-phosphate in mice liver using LC-MS analysis. Data are expressed as the mean ± SD.

Table S1. Mass parameters of prodrugs **1a-1j**.

Table S2. Summarized results of selectivity evaluation

Table S3. Detailed results for selectivity evaluation

Table S4. Detailed results LOD determination of LOD

Table S5. Detailed results of LOQ determination

Table S6. Summarized results for stability evaluation under auto-sampler conditions

Table S7. Detailed results of stability evaluation under auto sampler conditions

Table S8. Summarized results of stability analysis on bench top conditions

Table S9. Detailed results of stability analysis on bench top conditions

Table S10. Summarized results of matrix effect evaluation

Table S11. Detailed results of matrix effect evaluation

Table S12. Summarized results of recovery evaluation

Table S13. Detailed results of recovery evaluation

Table S14. Summarized results of carry over evaluation

Table S15. Detailed results of carry over evaluation

Table S16. Detailed results of accuracy analysis – Intraday 1

Table S17. Detailed results of accuracy analysis – Intraday 2

Table S18. Detailed results of accuracy analysis – Interday 1

Table S19. Detailed results of accuracy analysis – Interday 2

Table S20. Detailed results of accuracy analysis – Interday 3

Table S21. Detailed results of precision analysis – Intraday 1

Table S22. Detailed results of precision analysis – Intraday 2

Table S23. Detailed results of precision analysis – Inter Day 1

Table S24. Detailed results of precision analysis – Inter Day 2

Table S25. Detailed results of precision analysis – Inter Day 3

Table S26. Body weight and feed intake of animals used in repetitive single oral dosing of INH and pro\drug **1d**.

Table S27. Time versus plasma concentration profile of prodrug **1d** and *in vivo* released INH from **1d** post oral administration of single dose of prodrug **1d** at three incremental doses, 1mg/Kg, 3mg/Kg, 10mg/Kg.

Table S28. Time versus bntion profile of naïve INH post oral administration of single dose of naïve INH at three incremental doses, 1mg/Kg, 3mg/Kg and 10mg/Kg.

Table S29. Time versus plasma concentration profile of unchanged **1a**, **1d** and **1h** post oral administration of single dose (n=12) of prodrug **1a**, **1d** and **1h**.

Table S30. Time versus plasma concentration profile of *in* vivo free INH post oral administration of single dose (n=12) of naïve INH, prodrug **1a**, **1d** and **1h**.

Table S31. Time versus plasma concentration profile of *in* vivo released AcINH post oral administration of a single dose (n=12) of naïve INH, prodrug **1a**, **1d** and **1h**.

Table S32. Time versus plasma concentration profile of *in* vivo released INA post oral administration of a single dose (n=12) of naïve INH, prodrug **1a**, **1d** and **1h**.

Table S33. Time versus plasma concentration profile of *in* vivo released AcHz post oral administration of a single dose (n=12) of naïve INH, prodrug **1a**, **1d** and **1h**.

Table S34. Time versus plasma concentration profile of *in* vivo released Hz post oral administration of a single dose (n=12) of naïve INH, prodrug **1a**, **1d** and **1h**.

Table S35. Time versus plasma concentration profile of unchanged **1d** post intravenous administration of single dose (n=12) of prodrug **1d**.

Table S36. Time versus plasma concentration profile of *in* vivo released INH post intravenous administration of a single dose (n=12) of naïve INH and prodrug **1d**.

Table S37. Time versus plasma concentration profile of *in* vivo released AcINH post intravenous administration of a single dose (n=12) of naïve INH and prodrug **1d**.

Table S38. Time versus plasma concentration profile of *in* vivo released AcHz post intravenous administration of a single dose (n=12) of naïve INH and prodrug **1d**.

Table S39. Time versus plasma concentration profile of *in* vivo released Hz post oral administration of repetitive single dose (n=3) of naïve INH and prodrug **1d** for six consecutive days.

EXPERIMENTAL SECTION

General Chemistry information

All the reagents and starting materials were obtained from commercial sources like Sigma Aldrich or Merck and used as received. The solvents were purified and dried by standard procedures before use. Column chromatography was used for the isolation and purification of the final products from crude reaction mixtures using silica gel (100 – 200 mesh). Thin-layer chromatography was performed on silica gel and visualized using a UV chamber and Iodine. The ^{1}H and ^{13}C NMR spectra were recorded using a Bruker Avance 300 (300 MHz for ^{1}H and 75 MHz for ^{13}C) spectrometer in DMSO-d_6 using TMS as an internal standard. ^{1}H NMR spectra are reported in terms of chemical shift (δ, ppm) relative to the singlet at δ 2.50 ppm for dimethyl sulfoxide. ^{13}C NMR spectra were fully decoupled and are reported in terms of chemical shift (δ, ppm) relative to the septet at δ 39.5 ppm for DMSO-d_6. The chemical shifts (δ) for ^{1}H and ^{13}C are given in ppm relative to the residual signals of the solvent. Coupling constants are given in Hz. The following abbreviations are used to indicate the multiplicity: s, singlet; d, doublet; t, triplet; q, quartet; m, multiplet; br, broad signal. LCMS spectra were recorded on a Sciex API 6500+ Q-Trap mass spectrometer equipped with a turbo ion spray source in positive ion mode through direct infusion by syringe. HPLC purity, lipophilicity, chemical stability in pH buffers and solubility were determined using a Waters e-2695 Alliance separation HPLC module with an autosampler, column oven, injector, solvents (quaternary gradient mode) and a 2998 PDA detector. The chemical stability of prodrugs in DMSO was determined using Spectra Max M2 Multi-

Mode Microplate Reader (Molecular Devices) at a wavelength of 300nm. NMR spectra, HPLC chromatograms, and MS spectra of unknown compounds (**1a-1j**) are provided below.

Synthesis of Compound N-Acetyl Isoniazid (AcINH): To a stirring mixture of isoniazid (1.46 mmol; 200 mg) and pyridine (2 eq.) in DCM (8mL), acetyl chloride (2 eq.) was added. The reaction mixture was stirred for sixteen hours at room temperature, and the progress of the reaction was monitored on TLC. After complete consumption of the starting material, the reaction mixture was diluted with 10 mL of DCM and quenched with water. The organic layer was separated and dried using anhydrous sodium sulfate, filtered and concentrated under a vacuum to afford the crude desired product, which was further purified using silica gel column chromatography.

Synthesis of compound 1,2-bis((E)-4-methylbenzylidene)hydrazine (p-THz) for LC-MS method development: To a stirring mixture of hydrazine hydrate (1.99 mmol; 100 mg) in DCM (4mL), 4-methylbenzaldehyde (2 eq.) was added. The reaction was carried out by ultrasonication (40 KHz) for 50 min at room temperature and the progress of the reaction was monitored on TLC. After complete consumption of the starting material, the reaction mixture was diluted with 10 mL of DCM and quenched with water. The organic layer was separated and dried using anhydrous sodium sulfate, filtered and concentrated under a vacuum to afford the crude desired product, which was further purified using silica gel column chromatography.

General procedure for the synthesis of compounds 1a-1j: To a stirring mixture of THF-water (8 mL: 0.5 mL), isoniazid (0.7291 mmol; 100 mg) and respective chloroformate (1 eq.) were added. The reaction mixture was stirred for two to six hours, and the progress of the reaction was monitored on TLC. After complete consumption of the starting material, the reaction mixture was diluted with 10 mL of DCM and quenched with water. The organic layer was separated and dried using anhydrous sodium sulfate, filtered and concentrated under a vacuum to afford the crude desired product. Prodrug **1a-1j** were obtained in more than >95% purity post silica gel column chromatography using increasing concentration of Methanol in DCM as eluent. The chemical purity of all the compounds was determined using HPLC. The structural characterization of all compounds was done using ^{1}H and ^{13}C NMR spectroscopy.

5.3.1. N-acetylisonicotinoylhydrazine or Acetyl isoniazid ***(AcINH)***

Yellow solid; yield 79.6%**,** HPLC Purity 100%, melting point 190°C – 194°C; ^{1}H NMR (300 MHz, DMSO-d_6) δ 10.95 (s, 1H), 10.17 (s, 1H), 8.96 (d, *J* = 6.5 Hz, 2H), 8.11 (d, *J* = 6.5 Hz, 2H), 1.95 (s, 3H). ^{13}C NMR (76 MHz, DMSO-d_6) δ 168.8, 163.0, 146.8, 143.7, 123.5, 20.7; LCMS (ESI$^+$) calcd for $C_8H_{10}N_3O_3$ $(M+H)^+$, 180.1; found, 180.0.

5.3.2. 1,2-bis((E)-4-methylbenzylidene)hydrazine ***(p-THz)***

Yellow solid; yield 90%; HPLC Purity, 99.4%; melting point 146°C-150°C; ^{1}H NMR (300 MHz, DMSO-d_6) δ 8.67 (s, 2H), 7.77 (d, *J* = 8.3 Hz, 4H), 7.32 (d, *J* = 7.8 Hz, 4H), 2.37 (s, 6H). ^{13}C NMR (76 MHz, DMSO-d_6) δ 161.7, 141.8, 131.7, 130.0, 128.8, 21.6.; LCMS (ESI$^+$) calcd for $C_{13}H_{12}N_3O_3$ $(M+H)^+$, 236.1; found, 237.0.

5.3.3. Methyl-2-isonicotinoylhydrazine-1-carboxylate hydrochloride salt **(1a)**

White solid; yield 98%**;** HPLC purity 95.4%; melting point 182°C-184°C; ^{1}H NMR (300 MHz, DMSO-d_6) δ 10.91 (s, 1H), 9.50 (s, 1H), 8.93 (d, *J* = 6.4 Hz, 2H), 8.05 (d, *J* = 6.5 Hz, 2H), 3.65 (s, 3H). ^{13}C NMR (76 MHz, DMSO-d_6) δ 163.3, 156.5, 146.3, 143.6, 123.5, 52.2; LCMS (ESI$^+$) calcd for $C_8H_{10}N_3O_3$ $(M+H)^+$, 196.1; found, 196.1.

5.3.4. Ethyl-2-isonicotinoylhydrazine-1-carboxylate hydrochloride salt **(1b)**

Off white solid; yield 88%**;** HPLC purity 96.9%; melting point 184°C-192°C; ^{1}H NMR (300 MHz, DMSO-d_6) δ 10.89 (s, 1H), 9.44 (s, 1H), 8.93 (d, *J* = 6.5 Hz, 2H), 8.05 (d, *J* = 6.5 Hz, 2H), 4.10 (q, *J* = 7.3 Hz, 2H), 1.23 (t, *J* = 7.1 Hz, 3H). ^{13}C NMR (76 MHz, DMSO-d_6) δ 163.5, 156.1, 147.1, 142.9, 123.1, 60.9, 14.6; LCMS (ESI$^+$) calcd for $C_9H_{12}N_3O_3$ (M+H)$^+$, 210.1; found, 210.4.

5.3.5. Propyl-2-isonicotinoylhydrazine-1-carboxylate hydrochloride salt **(1c)**

Off white solid; yield 29.6%**,** HPLC purity 98.7%; melting point 142°C -160°C; ^{1}H NMR (300 MHz, DMSO-d_6) δ 10.86 (s, 1H), 9.44 (s, 1H), 8.91 (d, *J* = 6.3 Hz, 2H), 8.01 (d, *J* = 6.1 Hz, 2H), 4.01 (t, *J* = 6.8 Hz, 2H), 1.58 – 1.66 (m, 2H), 0.92 (t, *J* = 7.6 Hz, 3H). ^{13}C NMR (76 MHz, DMSO-d_6) δ 164.6, 156.7, 149.7, 141.1, 122.3, 66.7, 22.4, 10.6; LCMS (ESI$^+$) calcd for $C_{10}H_{14}N_3O_3$ (M+H)$^+$, 223.1; found, 224.1.

5.3.6. Isopropyl-2-isonicotinoylhydrazine-1-carboxylate hydrochloride salt **(1d)**

Off white solid; yield 33.4%**;** HPLC purity 98.0%; melting point 196°C-202°C; ^{1}H NMR (300 MHz, DMSO-d_6) δ 10.55 (s, 1H), 9.27 (s, 1H), 8.76 (d, *J* = 6.1 Hz, 2H), 7.75 (d, *J* = 6.1 Hz, 2H), 4.76 – 4.88 (m, 1H), 1.23 (d, *J* = 6.1 Hz, 6H). ^{13}C NMR (76 MHz, DMSO-d_6) δ 164.6, 155.9, 150.5, 139.4, 121.3, 68.2, 21.9; LCMS (ESI$^+$) calcd for $C_{10}H_{14}N_3O_3$ (M+H)$^+$, 224.1; found, 224.2.

5.3.7. Butyl-2-isonicotinoylhydrazine-1-carboxylate hydrochloride salt **(1e)**

Off white solid; yield 36.1%**;** HPLC purity 95.7%; melting point 160°C-178°C; ^{1}H NMR (300 MHz, DMSO-d_6) δ 10.94 (s, 1H), 9.46 (s, 1H), 8.96 (d, *J* = 6.5 Hz, 2H), 8.10 (d, *J* = 4.9 Hz, 2H), 4.06 (t, *J* = 6.5 Hz, 2H), 1.56 – 1.61 (m, 2H), 1.43 – 1.27 (m, 2H), 0.91 (t, *J* = 7.2 Hz, 2H). ^{13}C NMR (76 MHz, DMSO-d_6) δ 162.9, 156.2, 145.1, 145.0, 124.3, 64.6, 30.7, 18.6, 13.7; LCMS (ESI$^+$) calcd for $C_{11}H_{16}N_3O_3$ (M+H)$^+$, 238.1; found, 238.1.

5.3.8. Isobutyl-2-isonicotinoylhydrazine-1-carboxylate hydrochloride salt **(1f)**

White solid; yield 33.6%; HPLC purity 98.9%; melting point 160°C-170°C; ^{1}H NMR (300 MHz, DMSO-d_6) δ 10.79 (s, 1H), 9.44 (s, 1H), 8.87 (d, *J* = 6.3 Hz, 2H), 7.95 (d, *J* = 5.7 Hz, 2H), 3.85 (d, *J* = 6.7 Hz, 2H), 1.81-1.97(m, 1H), 0.92 (d, *J* = 6.6 Hz, 6 H). ^{13}C NMR (76 MHz, DMSO-d_6) δ 162.9, 156.3, 145.2, 144.9, 124.4, 70.7, 27.7, 18.9; LCMS (ESI$^+$) calcd for $C_{11}H_{16}N_3O_3$ (M+H)$^+$, 238.1; found, 238.3.

5.3.9. Allyl-2-isonicotinoylhydrazine-1-carboxylate hydrochloride salt **(1g)**

White solid; yield 59%; HPLC purity 98.4%; melting point 168°C-180°C; ^{1}H NMR (300 MHz, DMSO-d_6) δ 10.68 (s, 1H), 9.48 (s, 1H), 8.78 (d, *J* = 6.1 Hz, 2H), 7.77 (d, *J* = 6.1 Hz, 2H), 5.95 (m, 1H), 5.35 (d, *J* = 17.5 Hz, 1H), 5.24 (d, *J* = 10.5 Hz, 1H), 4.58 (d, *J* = 5.3 Hz, 2H). ^{13}C NMR (76 MHz, DMSO-d_6) δ 163.1, 155.9, 145.7, 144.4, 133.1, 124.0, 117.7, 65.3; LCMS (ESI$^+$) calcd for $C_{10}H_{12}N_3O_3$ (M+H)$^+$, 222.1; found, 222.2.

5.3.10. Benzyl-2-isonicotinoylhydrazine-1-carboxylate hydrochloride salt **(1h)**

White solid; yield 76%; HPLC purity 98.7%; melting point 122°C-130°C; ^{1}H NMR (300 MHz, DMSO-d_6) δ 10.67 (s, 1H), 9.52 (s, 1H), 8.77 (d, *J* = 6.1 Hz, 2H), 7.76 (d, *J* = 6.1 Hz, 2H), 7.45 – 7.28 (m, 5H), 5.13 (s, 2H). ^{13}C NMR (76 MHz, DMSO-

d$_6$) δ 164.5, 156.6, 149.5, 148.3, 142.6, 136.8, 129.0, 128.6, 128.4, 123.2, 122.7, 66.7; LCMS (ESI$^+$) calcd for $C_{14}H_{14}N_3O_3$ $(M+H)^+$, 272.1; found, 272.3.

5.3.11. Sec-butyl-2-isonicotinoylhydrazine-1-carboxylate hydrochloride salt **(1i)**

White solid; yield 34%; HPLC Purity 96.7%; melting point 176°C-178°C; ^{1}H NMR (300 MHz, DMSO-d$_6$) δ 10.60 (s, 1H), 9.27 (s, 1H), 8.76 (d, J = 6.1 Hz, 2H), 7.76 (d, J = 6.1 Hz, 2H), 4.61 – 4.72 (m, 1H), 1.51 – 1.60 (m, 2H), 1.20 (d, J = 6.4 Hz, 3H), 0.89 (t, J = 7.4 Hz, 3H). ^{13}C NMR (76 MHz, DMSO-d$_6$) δ 164.6, 156.1, 150.5, 137.3, 121.2, 72.6, 28.5, 19.6, 9.5; LCMS (ESI$^+$) calcd for $C_{11}H_{16}N_3O_3$ $(M+H)^+$, 238.1; found, 238.1.

5.3.12. Phenyl-2-isonicotinoylhydrazine-1-carboxylate hydrochloride salt **(1j)**

Off white solid; yield 98 %; HPLC Purity 98.1%; melting point 180°C-184°C; ^{1}H NMR (300 MHz, DMSO-d$_6$) δ 10.86 (s, 1H), 10.04 (s, 1H), 8.79 (d, J = 6.1 Hz, 2H), 7.79 (d, J = 6.1 Hz, 2H), 7.43 (d, J = 7.8 Hz, 2H), 7.26 (t, J = 7.4 Hz, 1H), 7.16 (d, J = 7.3 Hz, 2H). ^{13}C NMR (76 MHz, DMSO-d$_6$) δ 164.6, 154.5, 150.56, 150.56, 139.1, 129.6, 125.5, 121.5, 121.3; LCMS (ESI$^+$) calcd for $C_{13}H_{12}N_3O_3$ $(M+H)^+$, 258.1; found, 258.1.

HPLC/ UPLC method used to determine chemical purity, lipophilicity and chemical stability in pH buffers: The chemical purity, lipophilicity and chemical stability in two different pH buffers (pH~3.2 and pH~7.8) were analyzed using methods developed in either HPLC or UPLC. HPLC system consisted of a Waters e-2695 Alliance separation module with an autosampler, column oven, injector, solvents (quaternary gradient mode), and a 2998 PDA detector. Analysis was done using a Waters Sun Fire C_{18} column (4.6mm*250mm, 5µm) at 254nm. The mobile phase consisted of Water (A) and Acetonitrile (B). Runtime was 20 min. A gradient mode was used as follows: 0 – 3min, A:70%, B:30%; 3min – 5 min, A: 90%, B: 10%; 5min – 11min, A: 98%, B: 2%; 11 min – 13min, A: 30%, B: 70%; 13min – 16min, A: 10%, B: 90%; 16min – 17min, A: 2%, B: 98%; 17min – 20min, A: 70%, B: 30% at a flow rate of 1.2 ml/min. The injection volume was 10µl and the autosampler temperature was 20°C. Data analysis and data acquisition were done using Empower 3* software. A calibration curve of a range of 1ug/ml – 125ug/ml was used for the analysis. UPLC system included Waters Acquity H Class module with an autosampler, column oven, injector, solvents (quaternary gradient mode), and TUV detector. Analysis was done using the Acquity UPLC BEH C_{18} column (2.1mm*100mm, 1.7µm) at 254nm. The mobile phase consisted of Water (A) and Acetonitrile (B). Runtime was 10 min. 0 - 1min, A:95%, B:5%; 1min – 2min, A: 90%, B: 10%; 2min – 3min, A: 88%, B: 12%; 3min – 4min, A: 86%, B: 14%; 4min – 5min, A: 84%, B: 16%; 5min – 6min, A: 82%, B: 18%; 6min – 7min, A: 80%, B: 20%; 7min – 8min, A: 70%, B: 30%; 8min – 9.5min, A: 60%, B: 40%; 9.5min – 10min, A: 95%, B: 5% at a flow rate of 0.4 ml/min. The injection volume was 10µl and the autosampler temperature was 20°C. Data analysis and data acquisition were done using Empower 3* software. A calibration curve of a range of 1ug/ml – 125ug/ml was used for the analysis.

Lipophilicity, chemical stability and solubility determination method: The lipophilicity of **1a-1j** was analyzed using the octanol-water partition shake flask method as described before[1]. Briefly, 1mg of each prodrug was accurately weighed in a 2 mL micro-centrifuge tube. 500µL of octanol and 500µL of milli-Q water were added and stirred at 1000rpm for 3h at room temperature on a shaker. After incubation, the octanol and water layer were separated, reconstituted with mobile phase and analyzed in HPLC. LogP (predicted values) for prodrugs **1a-1j** was determined using ChemDraw Professional 16.0 software.

The solubility of prodrugs **1a-1j** was evaluated using a shake flask method, as reported in the literature[2]. A saturated solution of individual prodrug (**1a-1j)** was made in Tris HCl buffer with pH 7.4 and incubated at 37°C with 1000rpm for 24h in a shaker incubator. After incubation, an aliquot was withdrawn and filtered to ensure a clear solution. This clear sample was diluted in such a way that the analyte concentration may become 500μg/mL and injected into HPLC for analysis. The peak area was compared with the standard calibration curve to determine the dissolved concentration of the analyte.

The chemical stability of prodrugs **1a-1j** was assessed in DMSO, acidic pH buffer and basic pH buffer. For chemical stability analysis, 1mg of each prodrug was dissolved in 1 mL DMSO. 100μL of each solution was dispensed into 96 well plates. Analysis was done at two timepoints, i.e. 1hr and 24hr, using a UV-spectrophotometer. For pH stability analysis, an appropriate volume of standard stock solution of each analyte was incubated with 0.05M phosphate buffer, pH adjusted using orthophosphoric acid (acidic buffer, pH 3.2) and 0.05M phosphate buffer, pH adjusted using Tetrabutylammonium hydroxide (basic buffer, pH 7.6) for 24 hrs. Aliquots (100μL) were withdrawn at different time points, reconstituted with 100μL of mobile phase, and analyzed in HPLC. A control standard stock solution is used for comparison.

LCMS method used for bioanalytical quantifications: Analysis and quantification of prodrugs **1a-1j**, INH, AcINH, INA and AcHz during *ex vivo* ADME evaluation assays and *in vivo* pharmacokinetic analysis were performed using an LCMS/MS system included with Sciex – Exion HPLC system equipped with an autosampler, column oven, and binary pump which is coupled with a Sciex API 6500+ Q-Trap mass spectrometer equipped with turbo ion spray source. An Acquity UPLC BEH C_{18} column (100mm*2.1mm, 1.7μm) and a mobile phase consisting of 0.1% FA in water (A) and 0.1% FA in ACN (B) were used for analysis. Runtime was 5 min. An isocratic mode with a flow rate of 0.250μL has been used as follows: 0 - 5min, A:50%, B:50%. The volume of injection was 5μL. The autosampler and column oven were maintained at a temperature of 5°C and 40°C, respectively. The mass spectrometer was operated in a positive electron spray ionization mode.

Derivatization-based quantification of hydrazine in mice plasma using LC-MS: Hydrazine quantification in plasma during single oral dose (10mg/Kg) pharmacokinetic study was performed using a derivatization method with p-tolualdehyde, as described in the literature [3, 4]. Analysis was carried out on an Acquity UPLC BEH C_{18} column (100 mm × 2.1 mm, 1.7 μm) using a mobile phase of 0.1% formic acid in water (A) and 0.1% formic acid in acetonitrile (B). The total run time was 10 minutes. A gradient elution was employed at a flow rate of 0.350 mL/min as follows: 0–3 min: 15% A / 85% B; 3–6.8 min: 15% A / 85% B; 6.8–10 min: 80% A / 20% B, followed by re-equilibration to initial conditions. The injection volume was 10 μL.

Sample preparation for analytical quantifications: For quantification of prodrugs, INH, AcINH, INA and AcHz during calibration analysis, *ex vivo* plasma conversion assays, and *in vivo* pharmacokinetics studies, 25μL plasma samples were mixed with 25μL IS (Nicotinamide)[3] and deproteinized using 200μL acetonitrile. After vortexing for 2 min, samples were centrifuged at 10°C, 10000rpm for 10 min. The supernatant (50μL) was diluted with 450μL of 0.1% Formic acid in water and injected into LCMS for analysis.

Data analysis

Microsoft Office Excel 2019 was used for data analysis in stability studies and different parameters during LCMS method validation. GraphPad Prism 9.5.0 software was used to determine total plasma exposure over a period of time (eAUC) values and to determine the pharmacokinetic parameters of each prodrug, including *in vivo* released INH, AcINH, INA, and AcHz.

Bioconversion of prodrugs in plasma

To determine the bio reversibility of prodrugs **1a-1j**, 5µL of stock solutions of each prodrug in water at a concentration of 4mg/mL was added to a mixture of 25µL heparinized plasma (mice plasma and human plasma obtained from Innovative Research, Inc., USA IPLAWBNAH50ML-50059) and 20µL of Tris HCl buffer and kept for incubation for 20 hrs. Aliquots (25µL) were collected at four serial time points, 20min, 120min, 180min, and 20 hours, were mixed with 25µL IS (Nicotinamide) and deproteinized using 200µL acetonitrile, vortexed for 10min and centrifuged at 10°C for 10min at 10000rpm. Supernatants (50µL) were collected and reconstituted with mobile phase (450 µL) and analyzed using LCMS.

Animal experiments

All experimental *in vivo* work related to animals was performed after due approvals from the Institutional Animal Ethics Committee. All animal experiments were approved by the Institutional Animal Ethical Committee of Translational Health Science and Technology Institute. The protocols of these experiments are discussed below.

***In vivo* plasma exposure analysis after a single oral dose administration of INH and prodrugs 1a-1j to mice**

The reported RACE protocol[4] was followed to conduct this experiment. Briefly, animals were divided into eleven groups (Balb/C mice, n=3, age: 7–9 weeks). Each group of animals were orally administered with one of the prodrugs **(1a-1j)** at 10mg/kg concentration. 1% CMC was used as the vehicle. Blood was withdrawn at two timepoints i.e. 20min and 120min, by retroorbital puncture and was collected into 1.5mL microcentrifuge tubes coated with EDTA. Plasma was extracted and samples were stored at -80°C until further analysis in LCMS. Plasma exposure levels of *in vivo* released INH and AcINH from prodrugs **1a-1j** were evaluated.

***In vivo* plasma exposure analysis after repetitive single oral dosing of INH and prodrugs (1a-1h) for ten consecutive days**

Animals were divided into nine different groups (Balb/C mice, n=3, age: 12-14 weeks). Each group was dosed orally daily with one of the prodrugs **(1a-1h;** at a molar equivalent dose of 10mg/kg of INH) for ten consecutive days. INH at a dose of 10mg/kg was administered to one group of animals for comparative evaluation. 1% CMC was used as the vehicle. Blood samples were withdrawn at predetermined time points 20min, 120min and 6hr post-dose by retroorbital puncture on the first day and on the tenth day. Plasma samples were processed and stored at -80°C until further analysis using LCMS. Plasma exposure levels of INH, AcINH, INA and AcHz released *in vivo* from parent prodrugs **1a-1h,** were evaluated in this experiment.

Comparative plasma exposure analysis in mice following the oral administration of a single dose of 1d and INH at three incremental dose levels

Animals were divided into six groups (Balb/C mice, n=3/each group, age: 6-7 weeks) and orally administered with prodrug **1d** and INH. INH and prodrug **1d** were administered at three different doses: 1mg/kg, 3mg/kg and 10mg/kg using 1% CMC as the suitable vehicle. Blood samples were withdrawn at predetermined time points 20min, 120min and 6hr post-dose by retroorbital puncture. Plasma samples were processed and stored at -80°C until further analysis in LCMS. Plasma exposure levels of INH, AcINH, INA and AcHz, and their parent prodrug **1d** were quantified and evaluated using LCMS.

Complete pharmacokinetic analysis after oral administration of a single dose of INH and selected prodrugs (1d, 1a and 1h) in mice

Animals were divided into four groups (Balb/C mice, n=12/each group, age: 8–9 weeks). Three of the groups were dosed orally with prodrugs **1a, 1d,** and **1h** at a molar equivalent dose of 10mg/kg of INH. INH at a dose of 10mg/kg was administered to one of the groups of animals for comparative evaluation. 1% CMC has been used as a suitable vehicle. Blood samples were withdrawn at predetermined time points 0.083h, 0.33h, 1h, 2h, 4h, 6h and 24h post-dose by a retroorbital puncture (n=3/two timepoints; one group ~ n=3/three timepoints i.e. at 5min, 120min and 24hr). Plasma samples were processed and stored at -80°C until further analysis in LCMS. Plasma concentration levels of INH, AcINH, INA and AcHz and their parent prodrugs **1a, 1d** and **1h** were quantified and evaluated using LCMS. The complete pharmacokinetic profile of prodrugs **1a, 1d, 1h**, *in vivo* released INH, AcINH, INA were defined and compared with the PK profile of naïve INH.

Comparative pharmacokinetic analysis after intravenous administration of a single dose of 1d and INH in mice

Animals were divided into two groups (Balb/C mice, n=12/each group, age: 11-12 weeks) and intravenously administered with prodrug **1d** or INH**.** INH was administered at a dose of 1mg/kg and **1d** at a molar equivalent dose of 1mg/kg of INH. Normal saline solution was used as the suitable vehicle. Blood samples were withdrawn at predetermined time points 0.083h, 0.33h, 1h, 2h, 4h, 6h, 8h and 24h post-dose by retroorbital puncture (n=3/two timepoints). Plasma samples were processed and stored at -80°C until further analysis in LCMS. Plasma concentration levels of INH, AcINH, INA and AcHz and their parent prodrugs **1a, 1d** and **1h** were quantified and evaluated using LCMS. The complete pharmacokinetic profile of prodrugs **1a, 1d, 1h**, *in vivo* released INH, AcINH, INA were defined and compared with the PK profile of naïve INH.

Intestinal and stomach homogenate conversion assay of INH, 1a, 1d and 1h

To determine the bio reversibility of prodrugs in stomach and intestinal homogenate, 0.1 mL of 4mg/mL stock solution of each prodrug **1a, 1d, 1h** was incubated with 3.9mL of tissue homogenate for 20 hrs (Both stomach and intestinal mice tissue homogenate were prepared in-house following a reported method[5]). Aliquots (100µL) were collected at four time points, 20min, 120min, 180min and 20hours deproteinized using 400µL acetonitrile, vortexed for 10min and centrifuged at 10°C for 10min at 12800rpm. Supernatants (100µL) were collected and reconstituted with mobile phase (900 µL 0.1% FA in water) and analyzed using LCMS. Concentration levels of prodrugs **1a, 1d, 1h,** released INH, AcINH and INA were quantified and evaluated using LCMS.

Mouse liver homogenate conversion assay and human liver microsomal stability analysis of INH and 1d

To assess the stability of prodrug **1d** in liver tissue homogenate, 0.1 mL of a 4 mg/mL stock solution of **1d** was incubated with 3.9 mL of in-house prepared mouse liver homogenate, following a previously reported method [7], at 37°C for 2 hours. Aliquots of 50µL were collected at 15 minutes, 60 minutes, and 120 minutes. Each sample was deproteinized with 450µL of acetonitrile, vortexed for 10 minutes, and centrifuged at 10°C for 10 minutes at 12,800 rpm. Supernatants (50µL) were then reconstituted in 450µL of 0.1% formic acid in water and analyzed using LC-MS to quantify the remaining prodrug **1d** and released metabolites, including INH, AcINH, and INA.

Liver microsomal stability of prodrug **1d** and INH was evaluated using a literature-reported protocol [8–10]. In this assay, 50µM of either compound was incubated with 1 mg/mL of commercial human liver microsomes (Sigma Aldrich, M0567-

1VL) in the presence of 1 mM NADPH, in a total reaction volume of 500µL at 37°C. Aliquots (50µL) were collected at 0, 5, 30, 60, and 120 minutes, deproteinized with 450µL of ice-cold methanol, vortexed for 10 minutes, and centrifuged at 10°C for 10 minutes at 10,000 rpm. Supernatants (50µL) were reconstituted with 450µL of 0.1% formic acid in water and analyzed using LC-MS to quantify prodrug **1d** and free INH levels.

Evaluation of Serum alanine transaminase (ALT) and Aspartate transaminase (AST) after repetitive single oral dosing of INH and prodrug 1d for six consecutive days

In this study, Balb/C mice (n = 3 per group; age: 22–24 weeks) were divided into three groups and orally administered with either prodrug **1d**, INH, or vehicle control. INH was administered at 90 mg/kg as the reference dose. Given that **1d** (at molar equivalent dose of INH) produces a 1.45-fold higher INH AUC compared to native INH, its dose was adjusted accordingly. The molecular weight of INH is 137.139 and that of **1d** is 259.7, giving a molar ratio of 1.89. Therefore, the molar equivalent dose of **1d** corresponding to 90 mg/kg INH was calculated as 170.43 mg/kg. Adjusting for the 1.45-fold increase in AUC, the final administered dose of **1d** was 117.5 mg/kg (i.e., 170.43 ÷ 1.45). All compounds were formulated in 60% propylene glycol in water as the vehicle. On day 7, 24 hours after the sixth oral dose, blood samples were collected via retroorbital puncture. Plasma was separated and analyzed for serum ALT and AST levels using commercially available assay kits according to the manufacturers' protocols.

Derivatization-based quantification of hydrazine in mice plasma using LC-MS: To measure the plasma concentration levels of hydrazine, samples were processed with suitable derivatization using p-tolualdehyde, based on a reported method[3, 4], and analyzed using LC-MS.

Quantification of pyridoxal and pyridoxal-5-phosphate in mice liver: The tissue levels of pyridoxal and pyridoxal-5-phosphate in mice liver were determined using untargeted metabolomic analysis workflow. Samples were prepared based on the extraction method reported in literature.[6] Briefly, 0.1g of liver tissue samples were homogenized well in 300µL of water. An aliquot of 200µL of the tissue homogenate was been deproteinized using 400µL ice cold methanol, vortexed, centrifuged at 18000g at 10°C for 20min. 50µL of the supernatant was withdrawn, evaporated with a gentle nitrogen flow at room temperature and reconstituted with 1:1 v/v of 100µLwater and ACN and analysed in LCMS. The acquired metabolomics data were processed using the TidyMass project[7]. The raw data was first converted to the mzML format through ProteoWizard. Then peak picking and grouping were performed using the massProcessor package to extract the metabolic features. The data cleaning was performed using the massCleaner package, where features with 20% missing values in the QC samples or more than 50% missing values in all the study groups were considered noisy and excluded. The K-nearest neighbors (KNN) algorithm was applied to impute the missing values, and support vector regression (SVR) based data normalization was performed. The metabolite annotation was done using the metID package by matching the accurate mass, retention time, and fragmentation pattern against the in-house and public datasets. The Retention time tolerance was set at 30s.

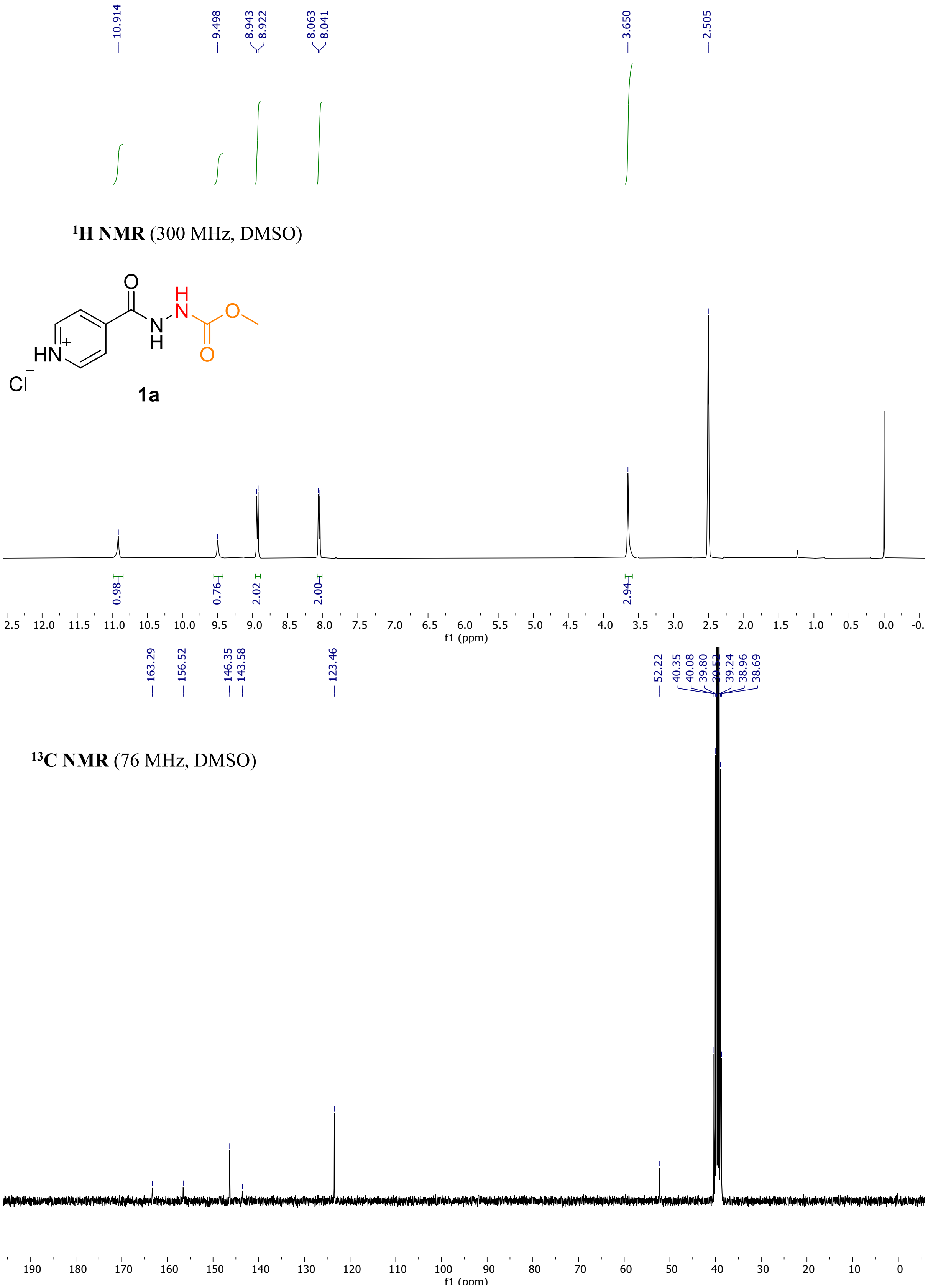

Figure S1. ^{1}H NMR and ^{13}C NMR of Prodrug **1a**.

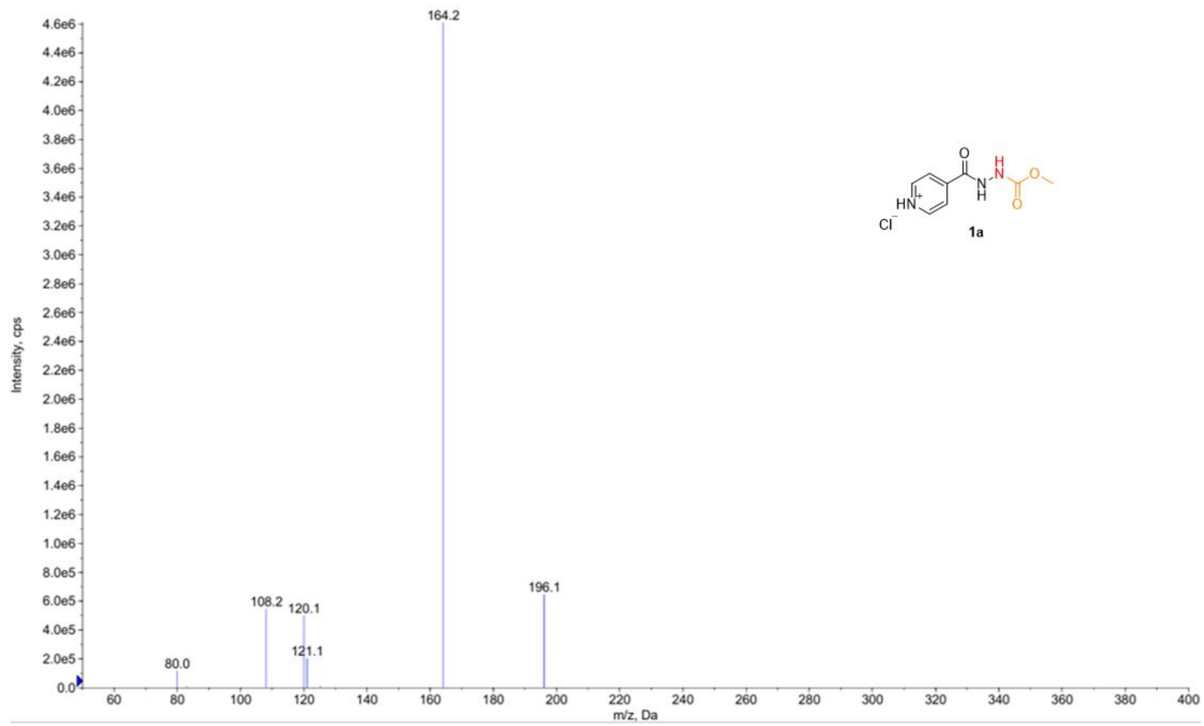

Figure S2. Mass spectra of **1a**

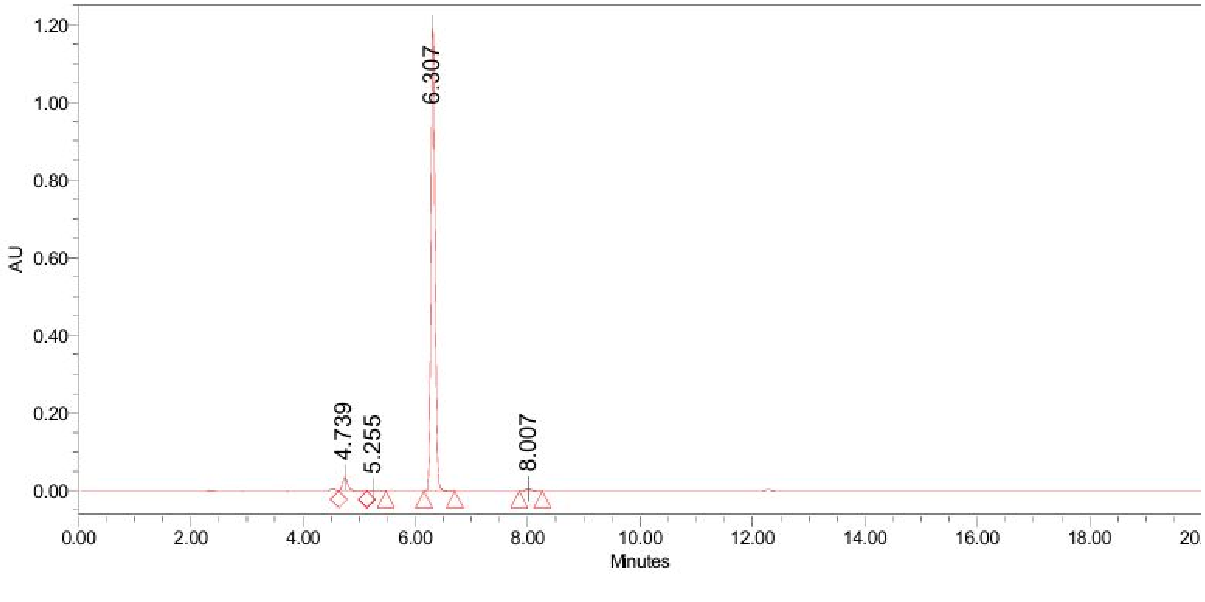

Peak Results

	Name	RT	% Area	Area	Height	Amount	Units
1		4.739	3.81	240994	35279		
2		5.255	0.11	6908	720		
3		6.307	95.44	6043686	1191415		
4		8.007	0.64	40561	5263		

Figure S3. HPLC Chromatogram of **1a**

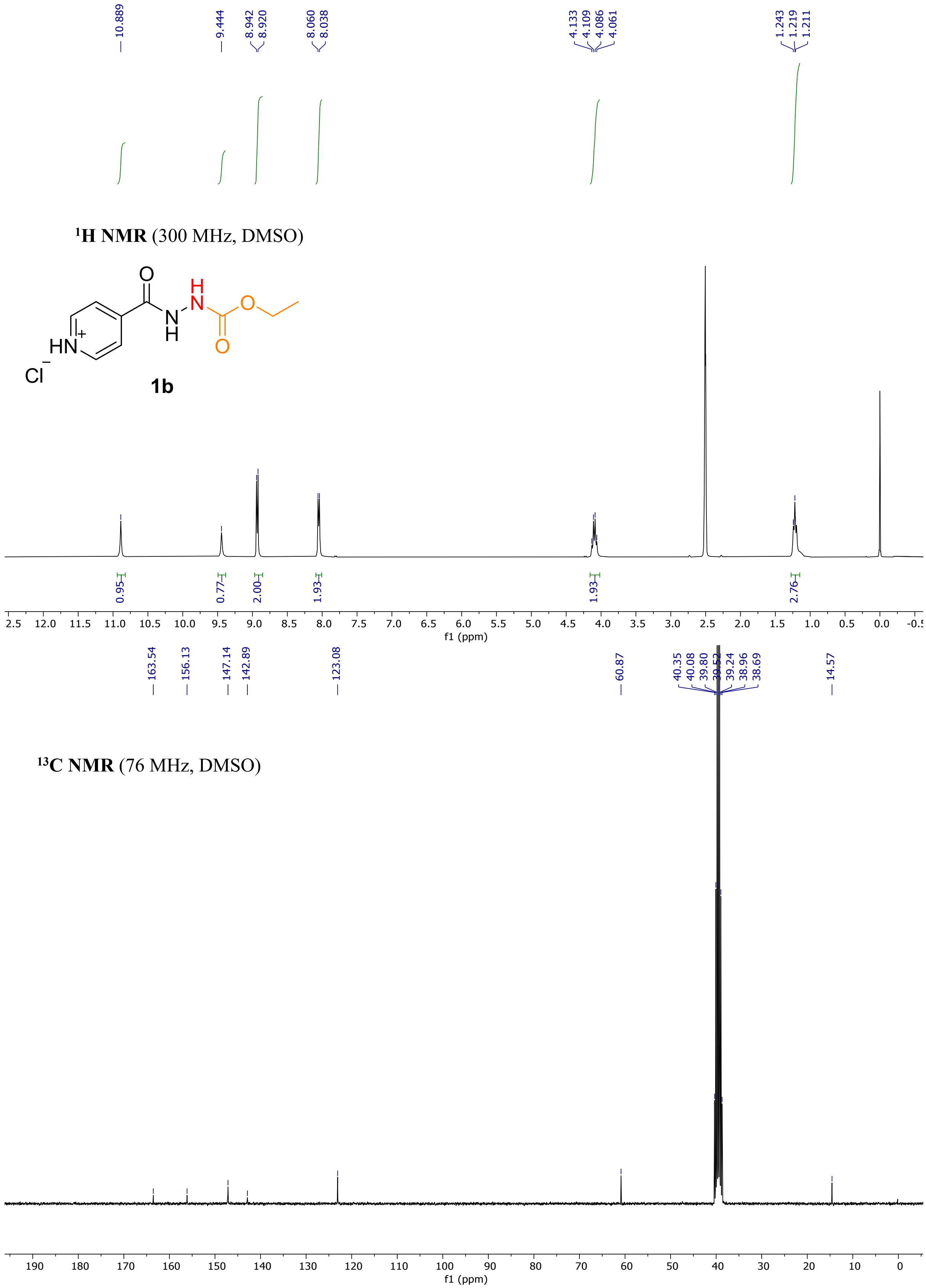

Figure S4. ^{1}H NMR and ^{13}C NMR of Prodrug **1b**.

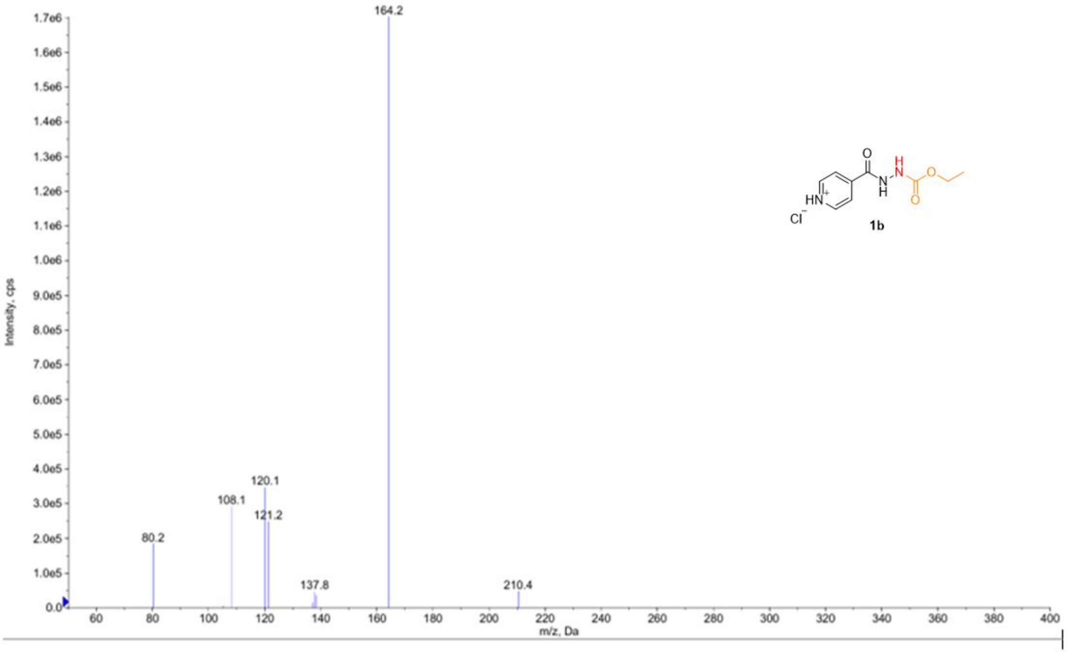

Figure S5. Mass spectra of **1b**

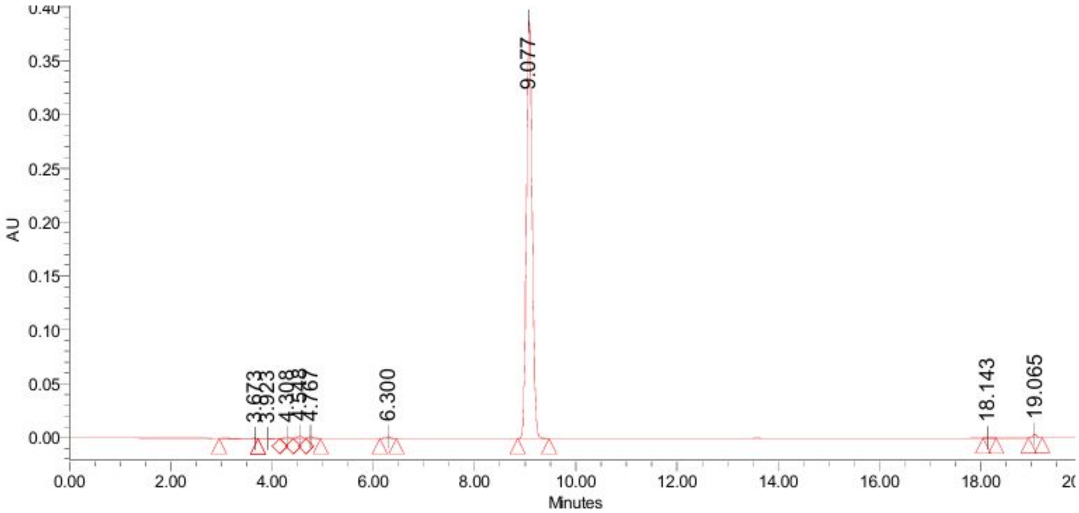

Peak Results

	Name	RT	% Area	Area	Height	Amount	Units
1		3.673	0.60	18653	598		
2		3.923	0.27	8443	398		
3		4.308	0.30	9302	932		
4		4.548	0.54	16759	2250		
5		4.767	0.39	12207	1839		
6		6.300	0.37	11533	1567		
7		9.077	96.93	3030726	387724		
8		18.143	0.08	2359	425		
9		19.065	0.53	16674	3288		

Figure S6. HPLC Chromatogram of **1b**

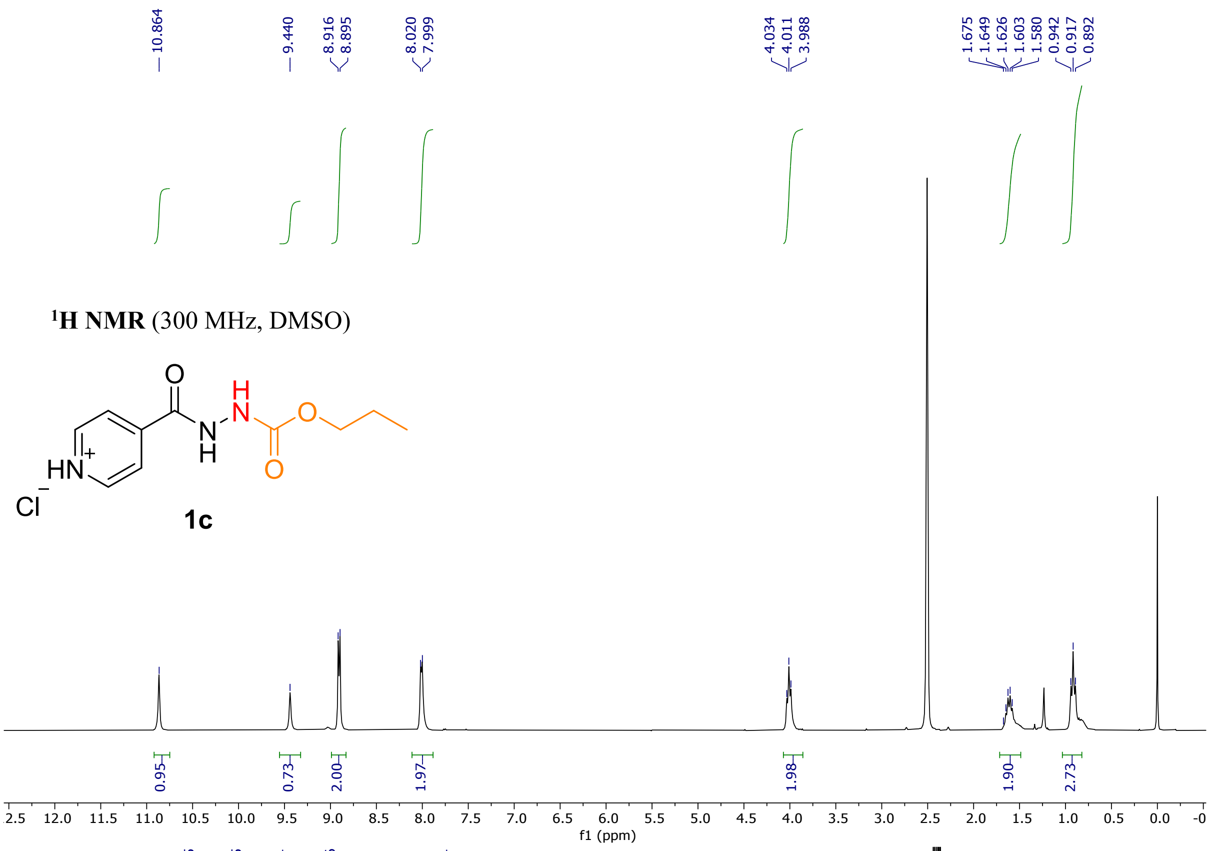

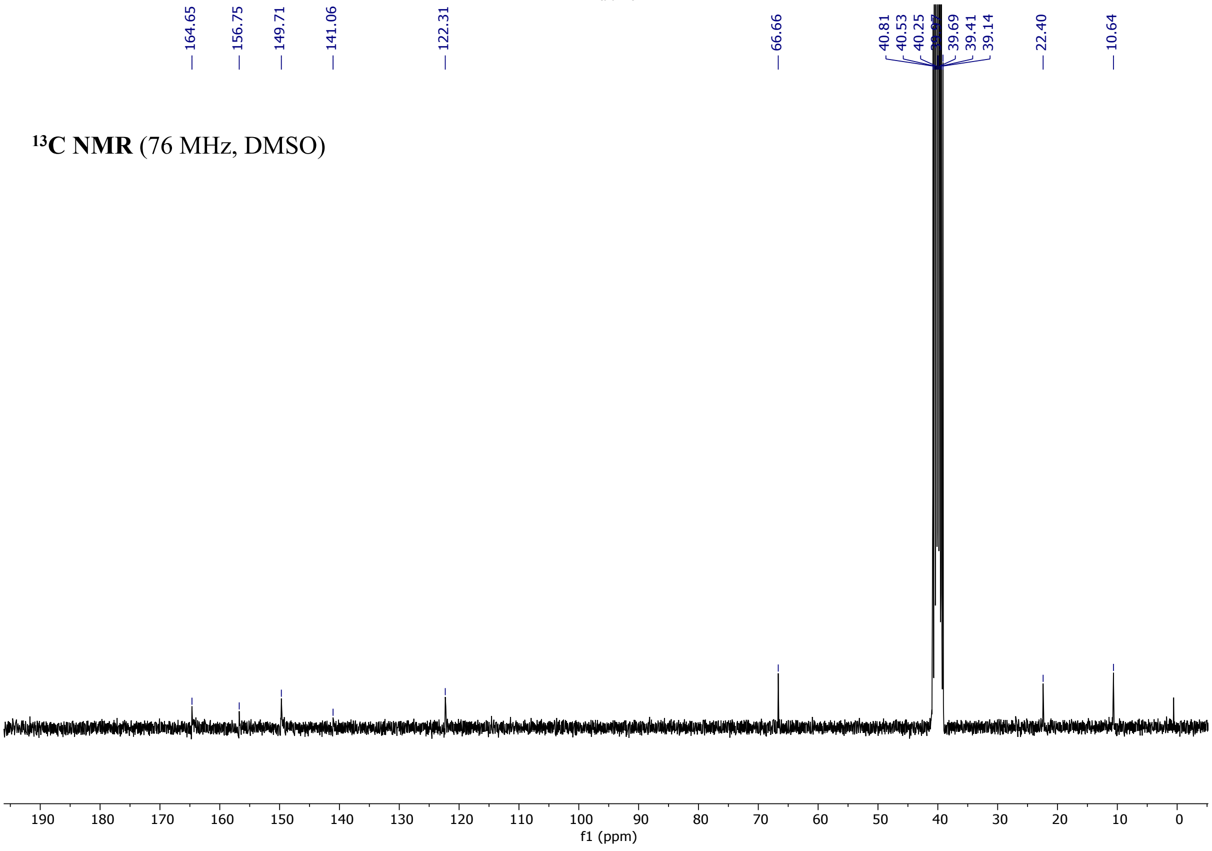

Figure S7. ^{1}H NMR and ^{13}C NMR of Prodrug **1c**.

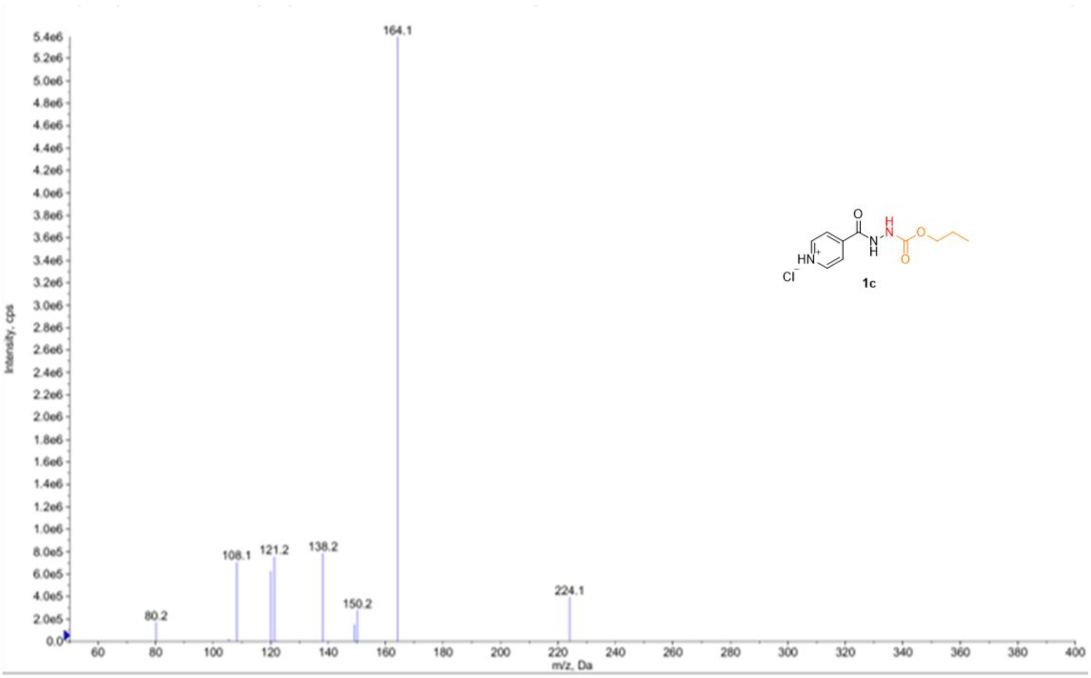

Figure S8. Mass spectra of **1c**

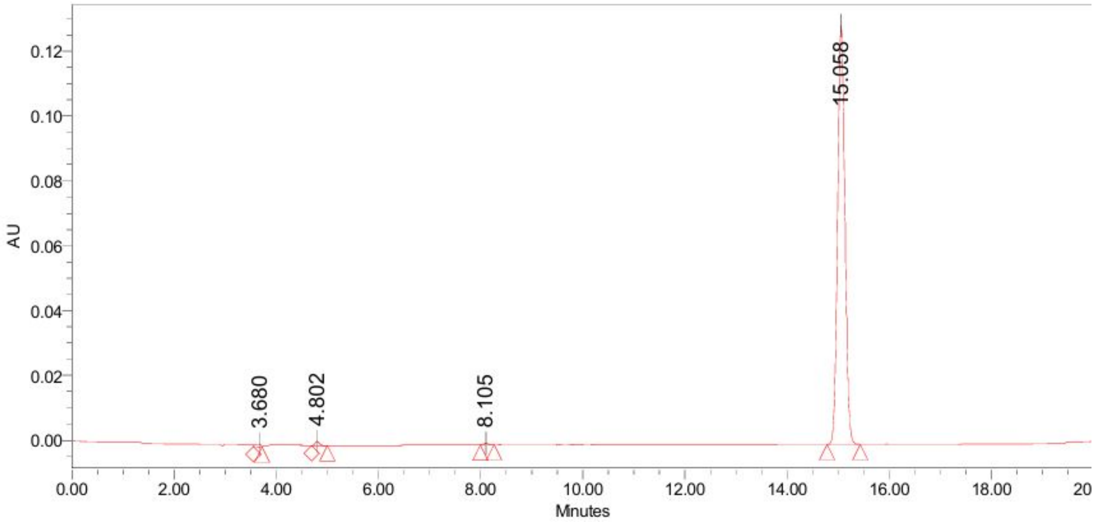

Peak Results

	Name	RT	% Area	Area	Height	Amount	Units
1		3.680	0.44	5691	766		
2		4.802	0.61	7810	1294		
3		8.105	0.26	3371	527		
4		15.058	98.69	1271675	129145		

Figure S9. HPLC Chromatogram of **1c**

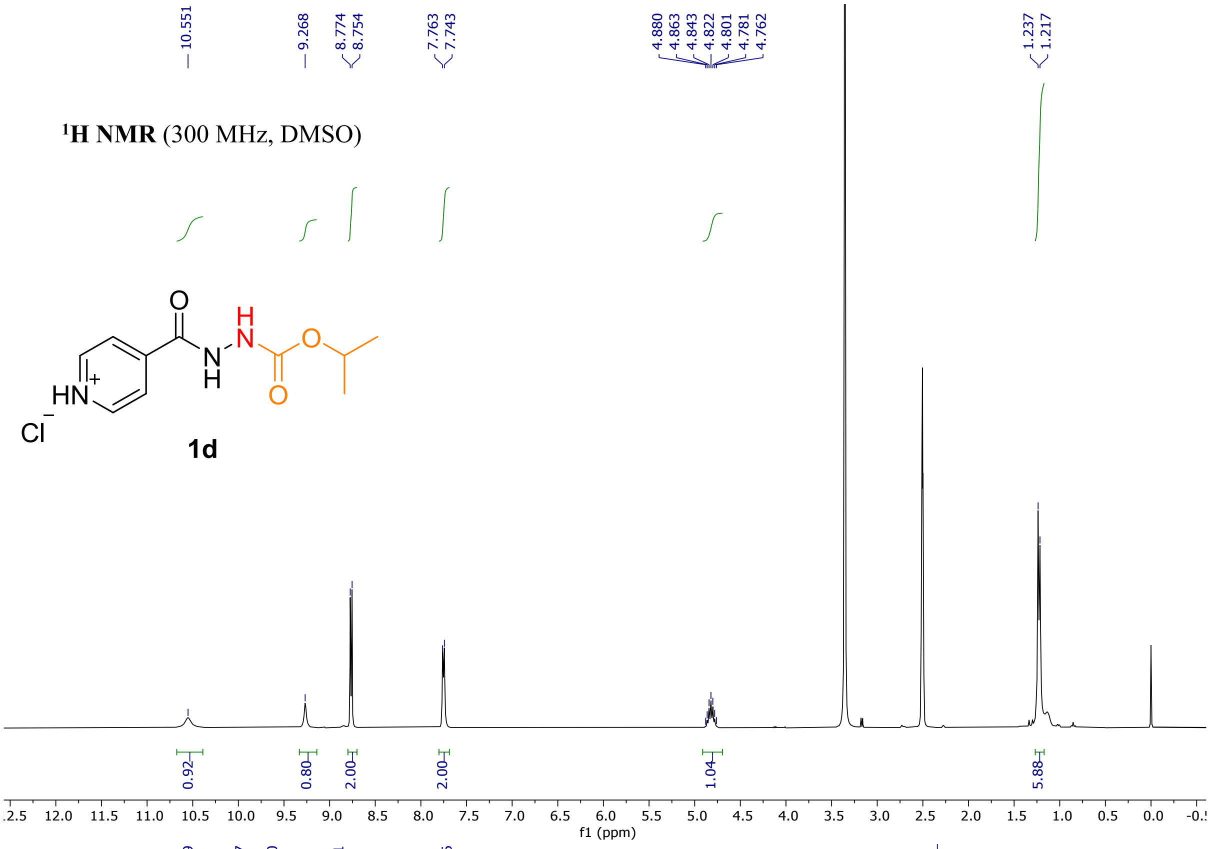

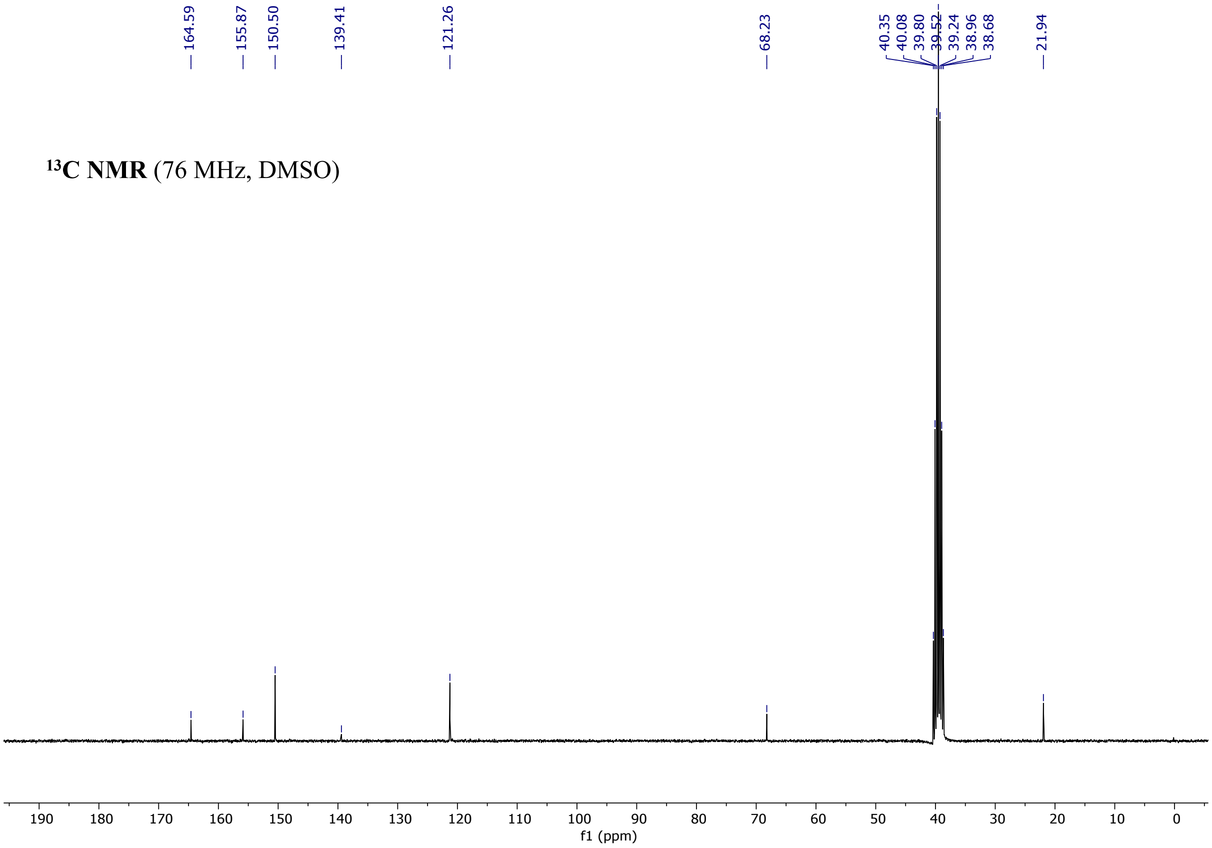

Figure S10. ^{1}H NMR and ^{13}C NMR of Prodrug **1d**.

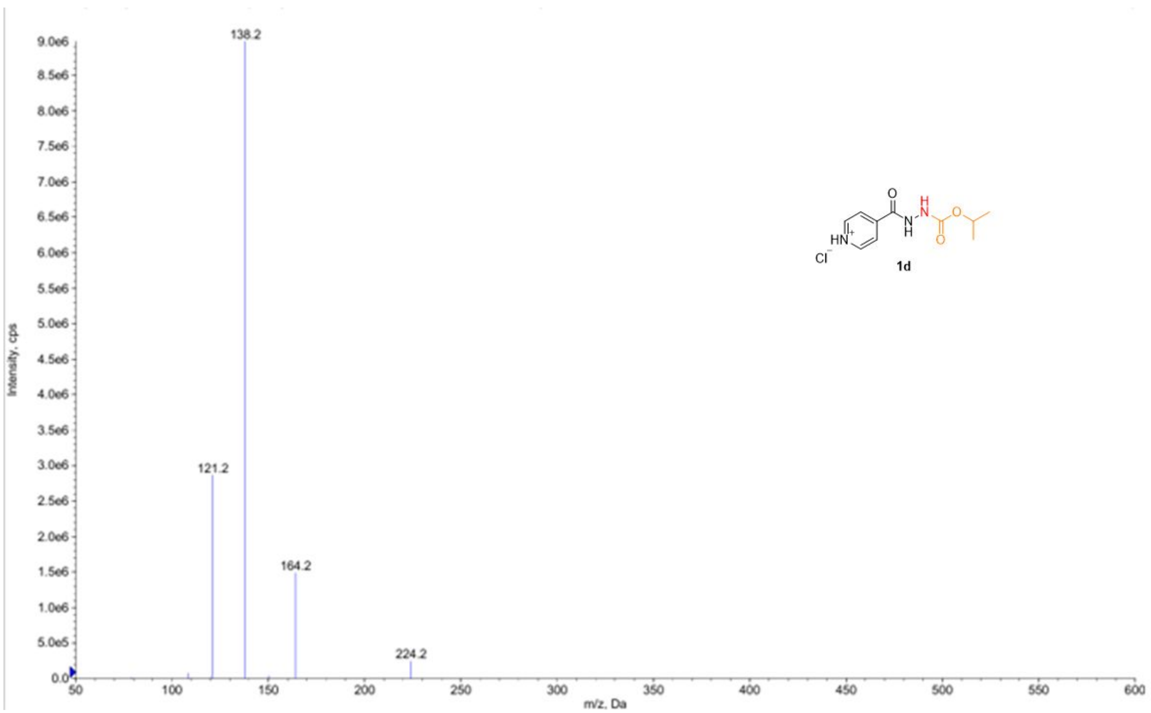

Figure S11. Mass spectra of **1d**

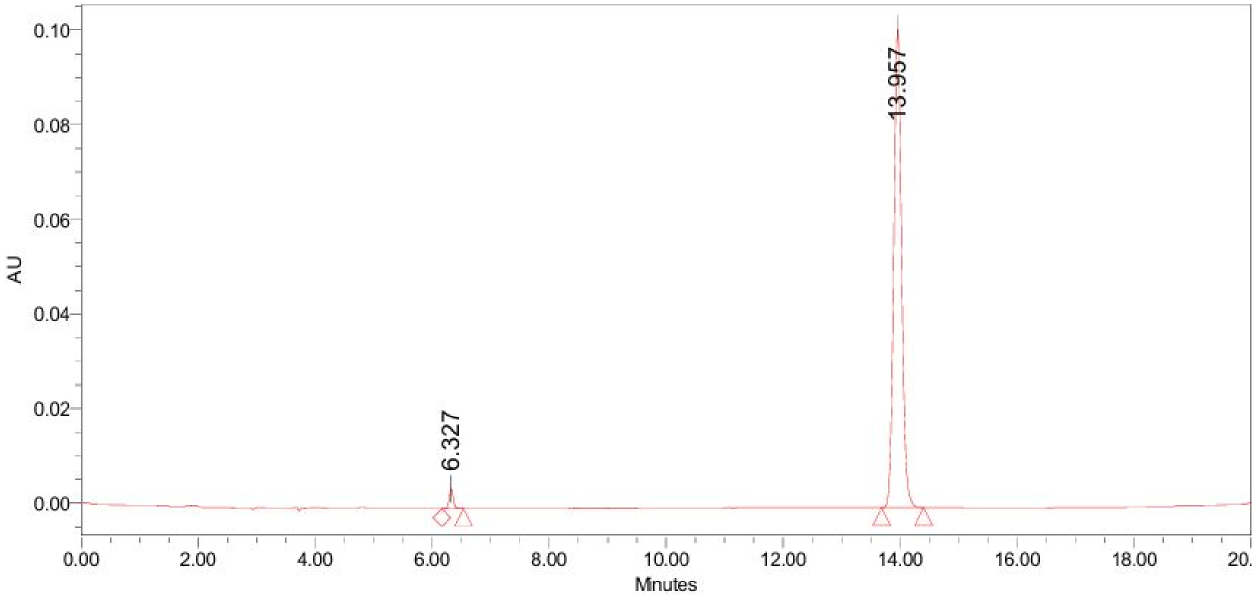

Peak Results

	Name	RT	% Area	Area	Height	Amount	Units
1		6.327	1.98	19392	4193		
2		13.957	98.02	961275	101266		

Figure S12. HPLC Chromatogram of **1d**

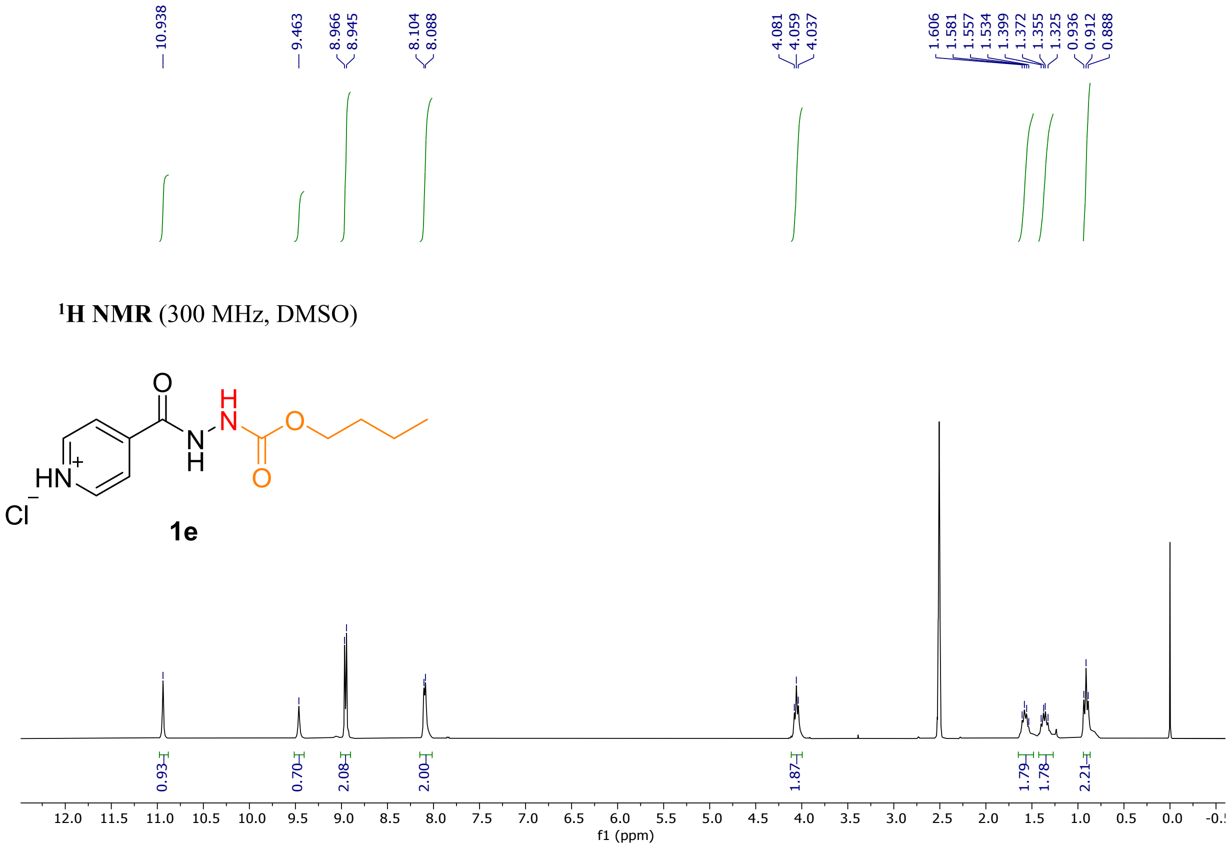

^{1}**H NMR** (300 MHz, DMSO)

1e

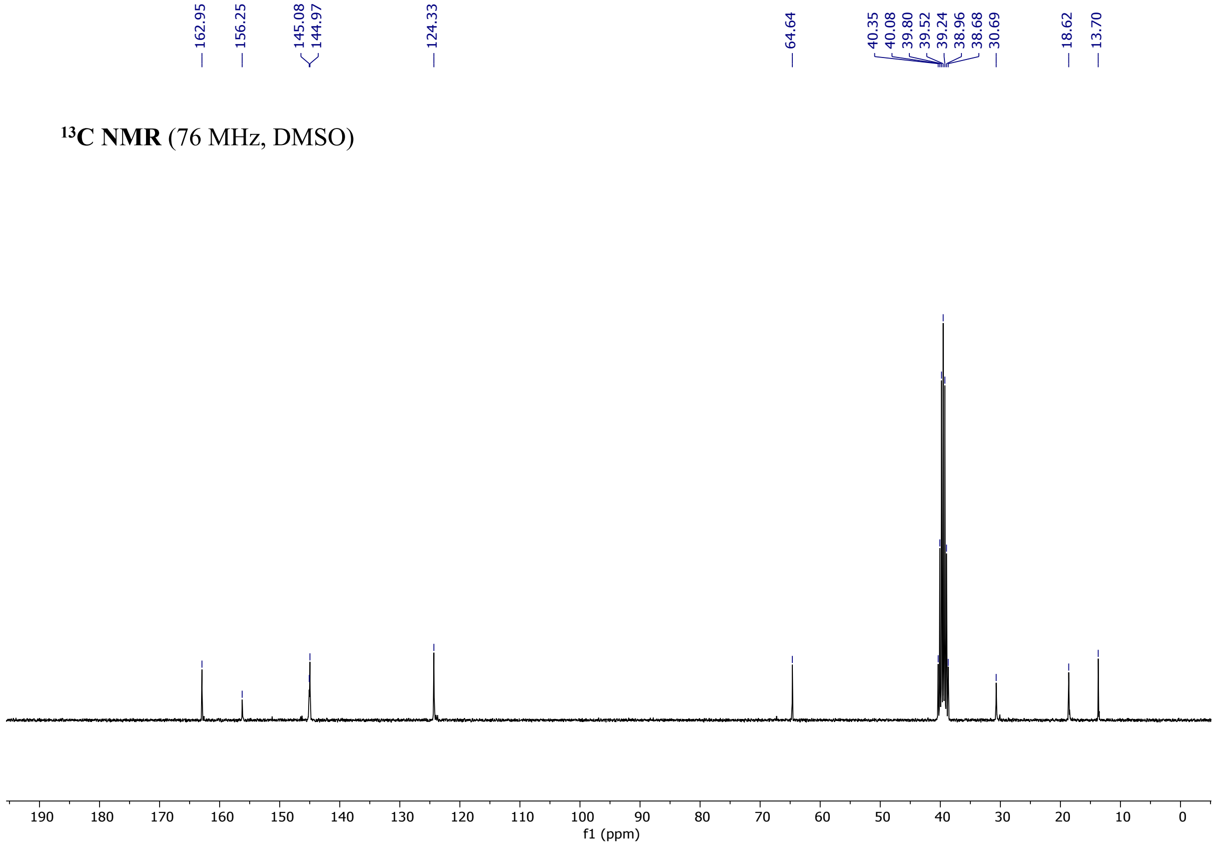

^{13}**C NMR** (76 MHz, DMSO)

Figure S13. ^{1}H NMR and ^{13}C NMR of Prodrug **1e**.

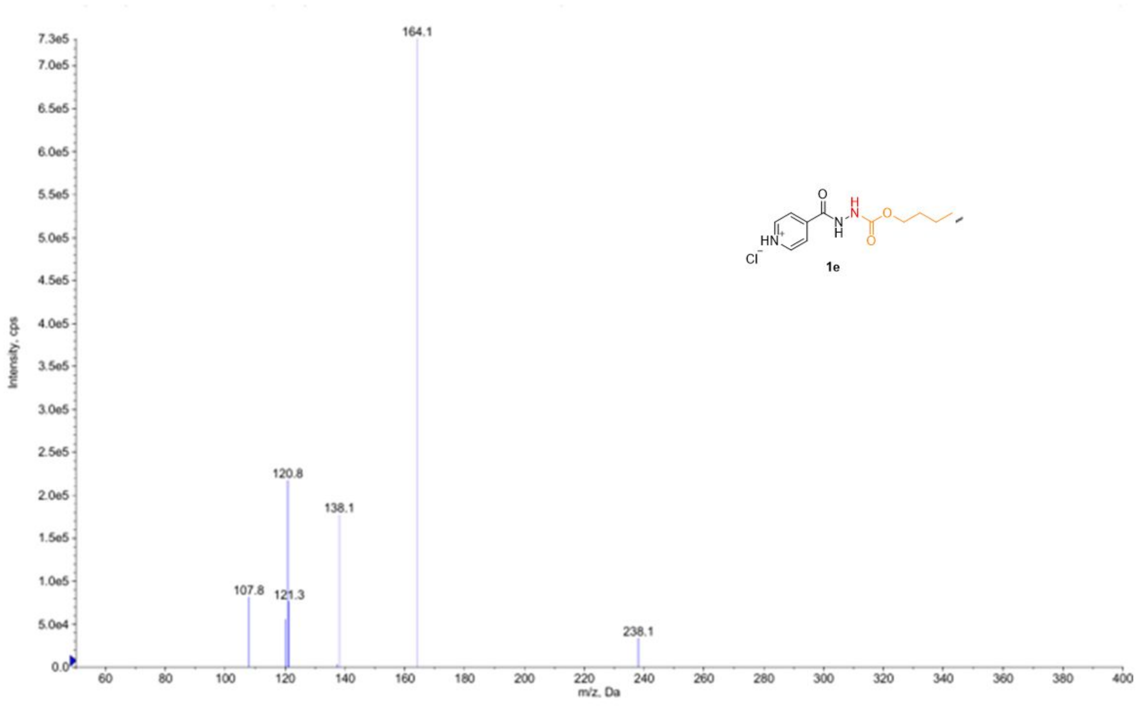

Figure S14. Mass spectra of **1e**

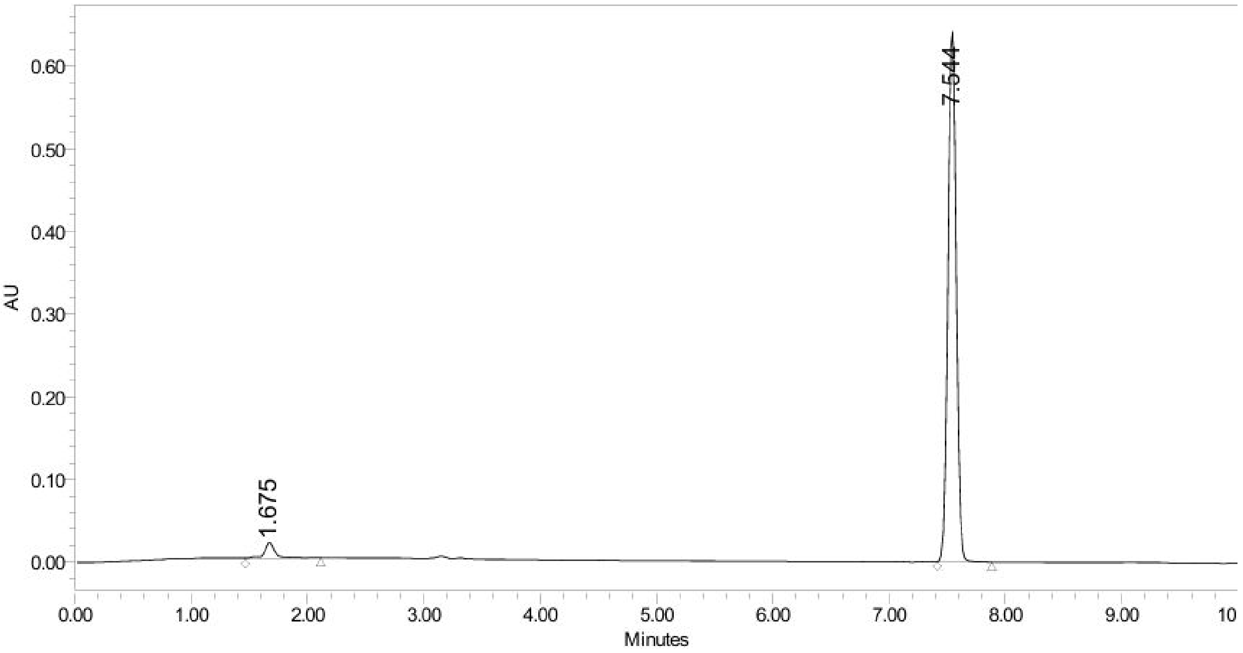

	RT	Area	% Area	Height
1	1.675	141576	4.26	19330
2	7.544	3182079	95.74	639693

Figure S15. HPLC Chromatogram of **1e**

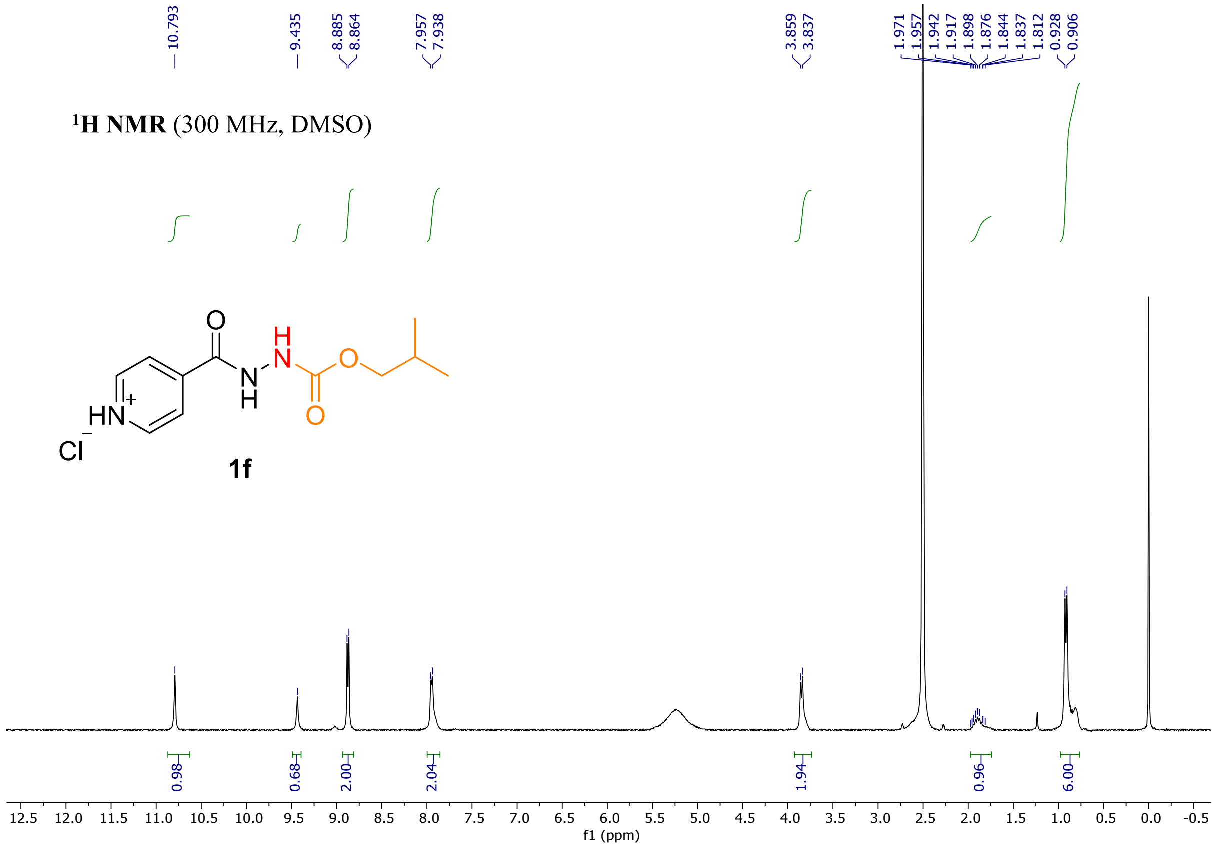

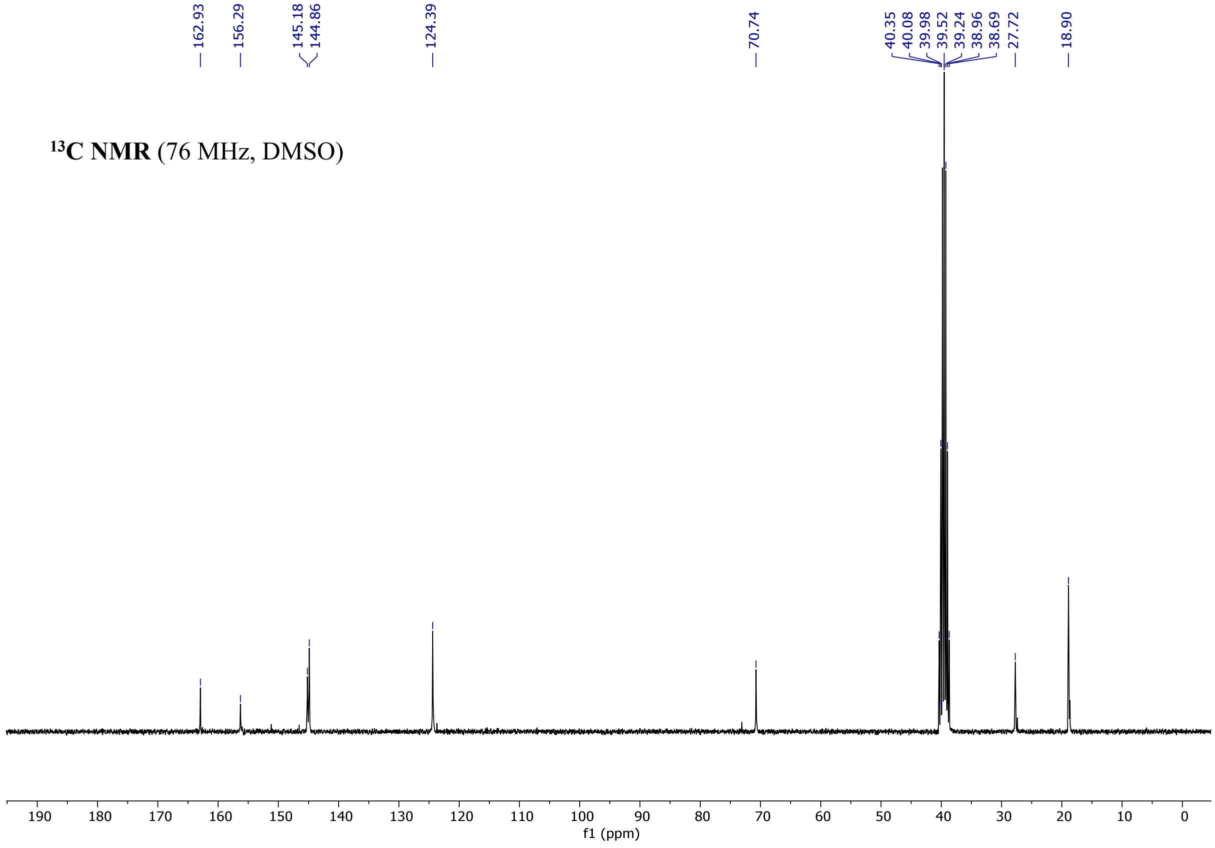

Figure S16. ^{1}H NMR and ^{13}C NMR of Prodrug **1f**.

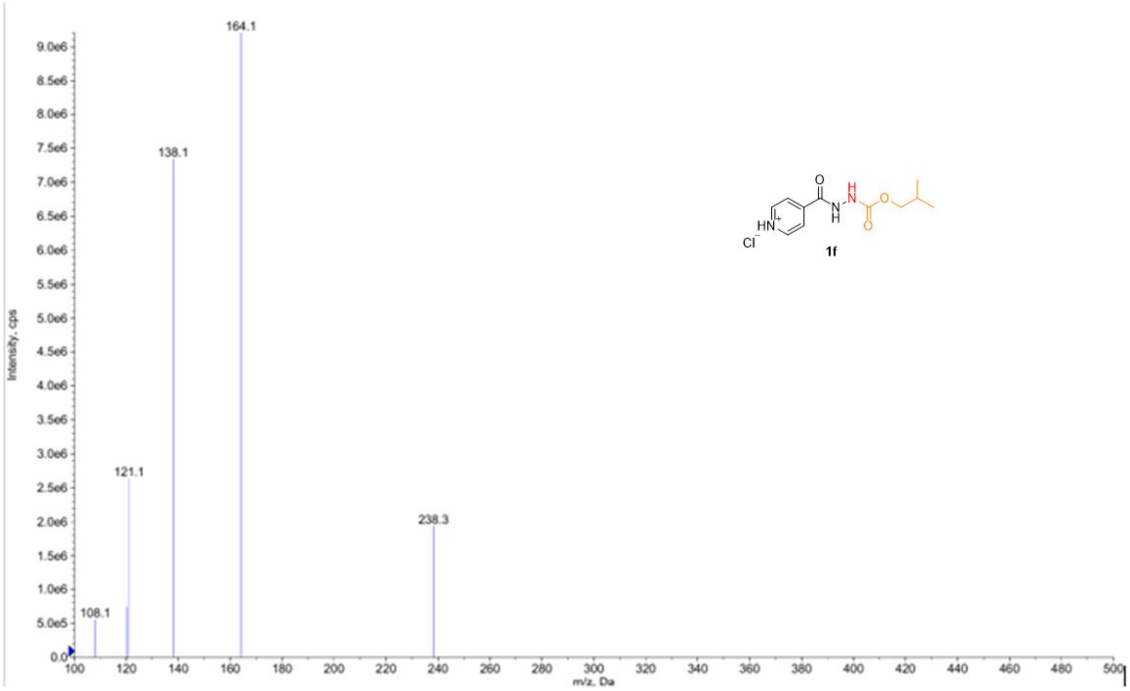

Figure S17. Mass spectra of **1f**

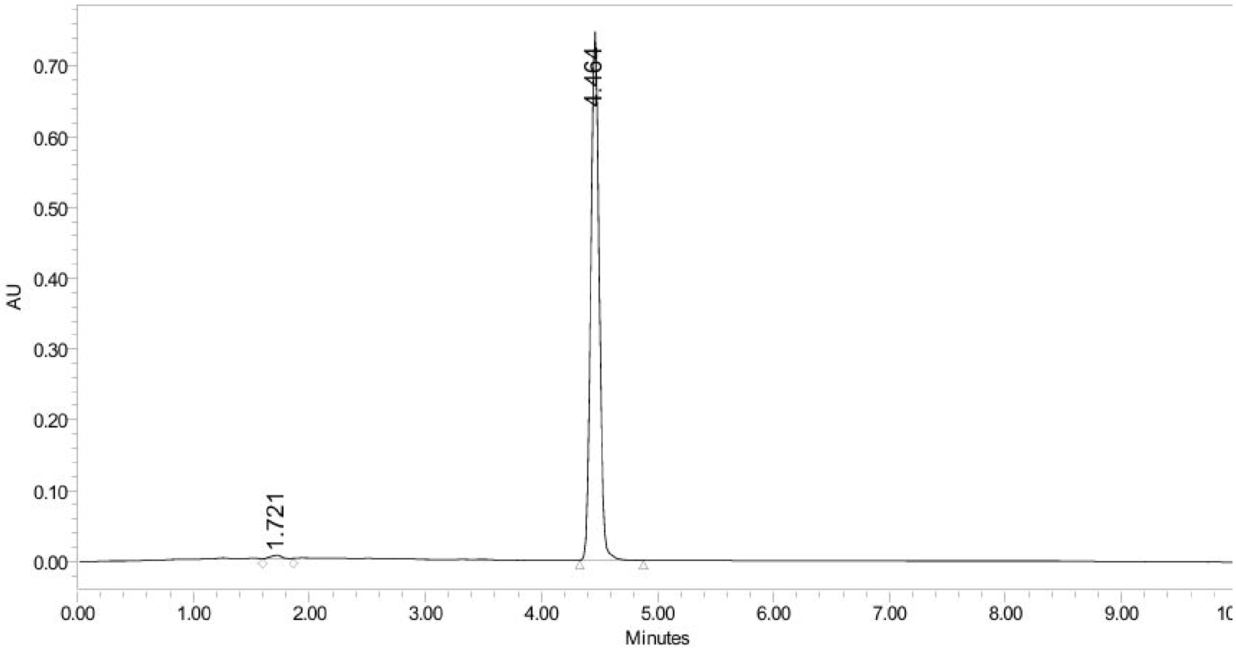

	RT	Area	% Area	Height
1	1.721	38397	1.01	5154
2	4.464	3749898	98.99	739046

Figure S18. HPLC Chromatogram of **1f**

^{1}H NMR (300 MHz, DMSO)

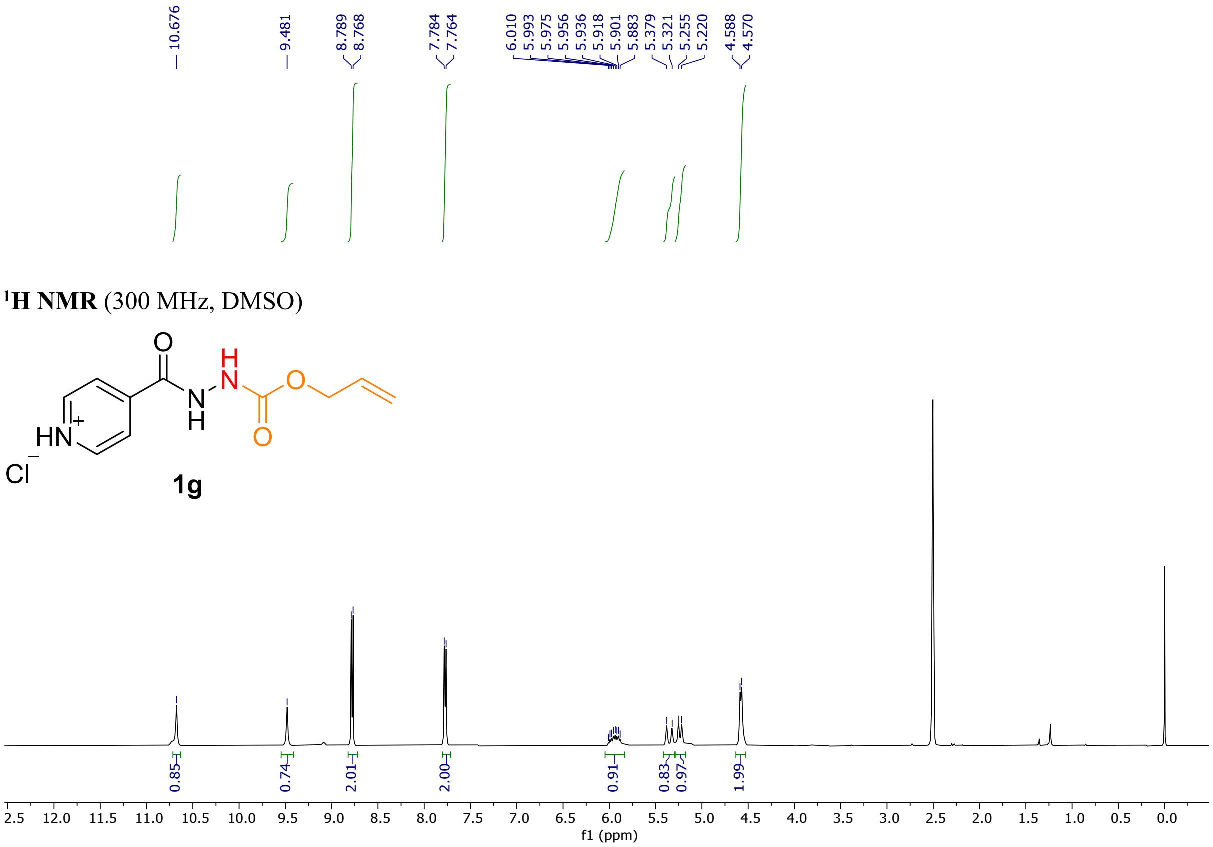

^{13}C NMR (76 MHz, DMSO)

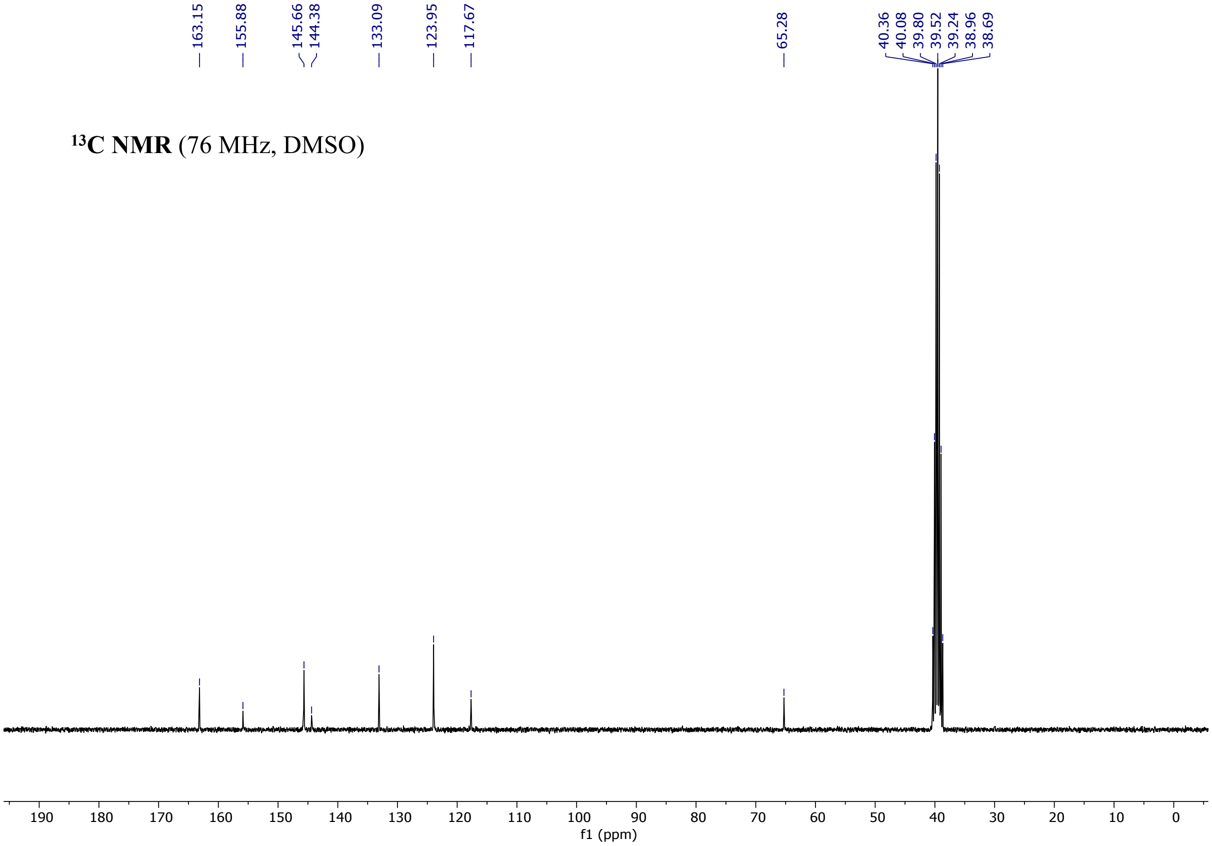

Figure S19. ^{1}H NMR and ^{13}C NMR of Prodrug **1g**.

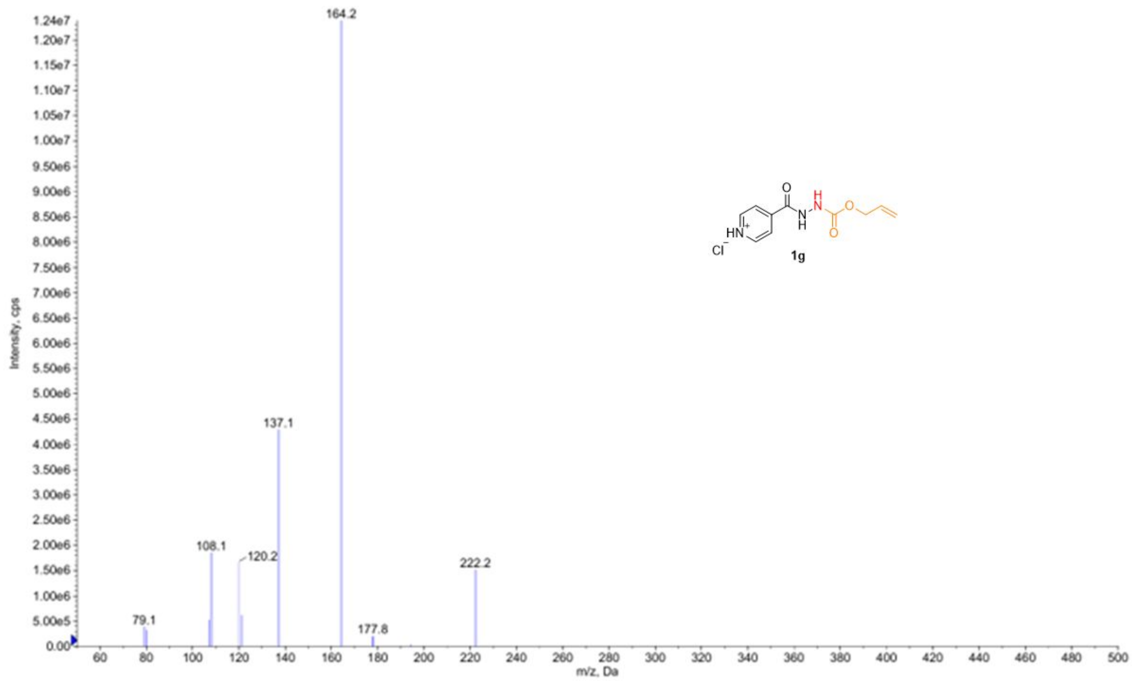

Figure S20. Mass spectra of **1g**

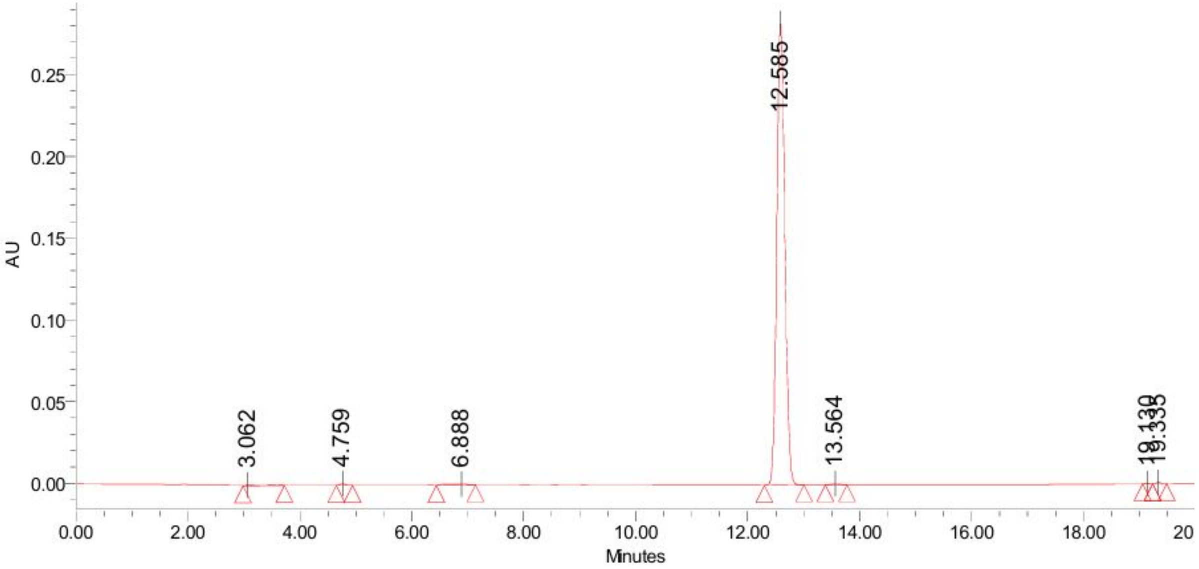

Peak Results

	Name	RT	% Area	Area	Height	Amount	Units
1		3.062	0.46	12942	413		
2		4.759	0.21	5857	963		
3		6.888	0.54	15327	604		
4		12.585	98.36	2782938	281958		
5		13.564	0.25	7206	744		
6		19.130	0.05	1315	281		
7		19.335	0.13	3799	780		

Figure S21. HPLC Chromatogram of **1g**

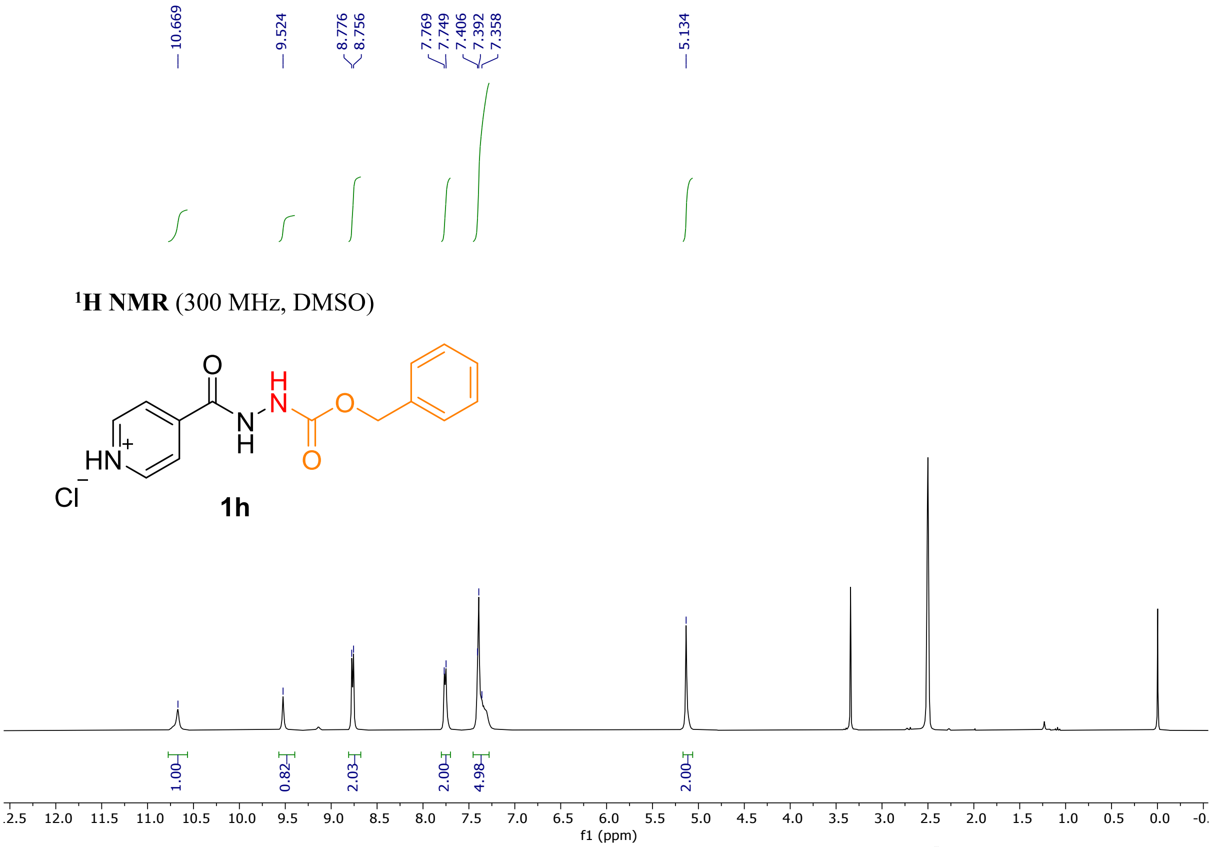

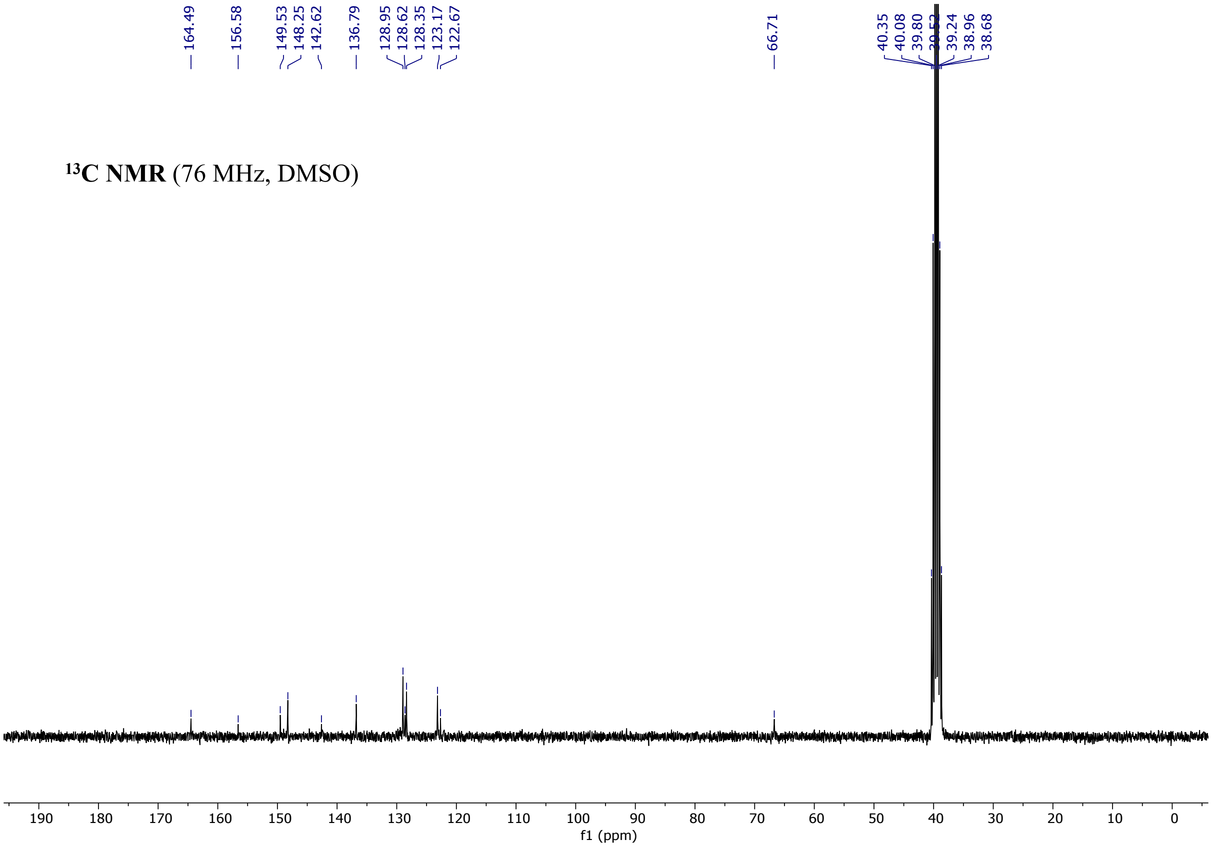

Figure S22. ^{1}H NMR and ^{13}C NMR of Prodrug **1h**.

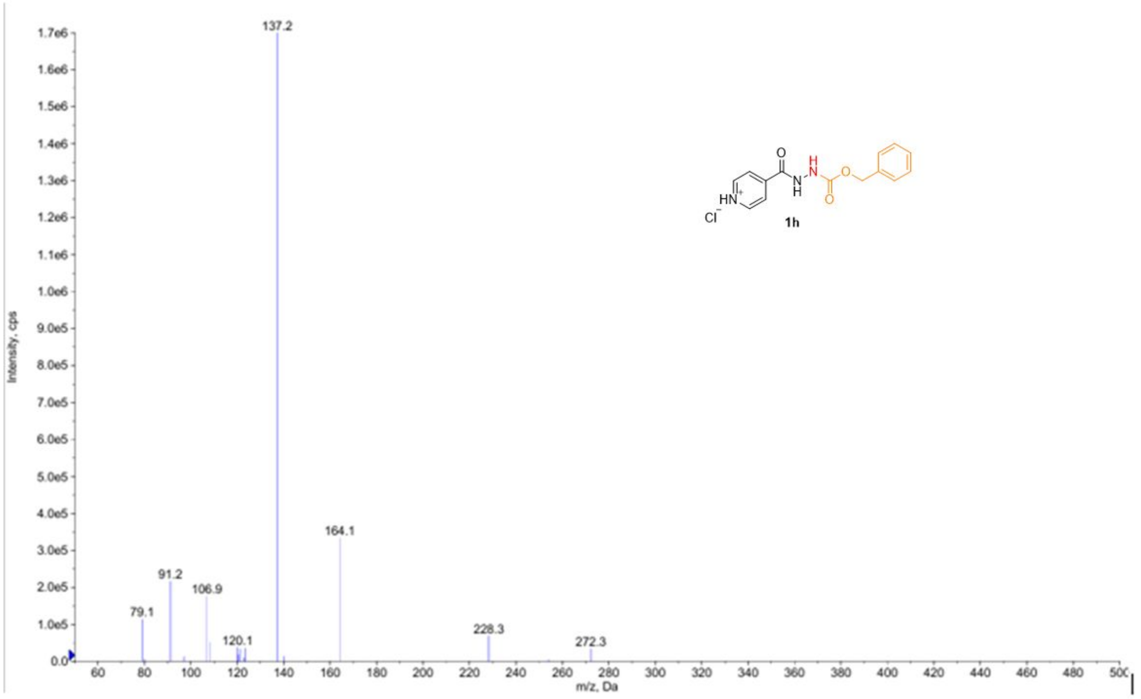

Figure S223. Mass spectra of **1h**

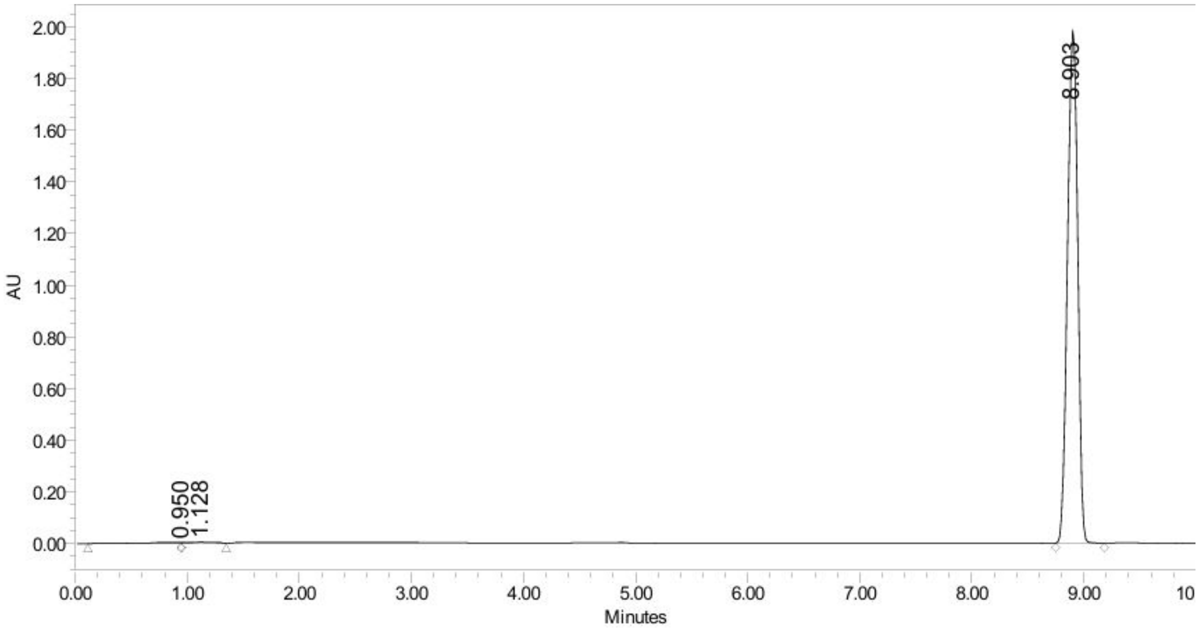

	RT	Area	% Area	Height
1	0.950	79011	0.62	3028
2	1.128	84891	0.67	5186
3	8.903	12544290	98.71	1986635

Figure S24. HPLC Chromatogram of **1h**

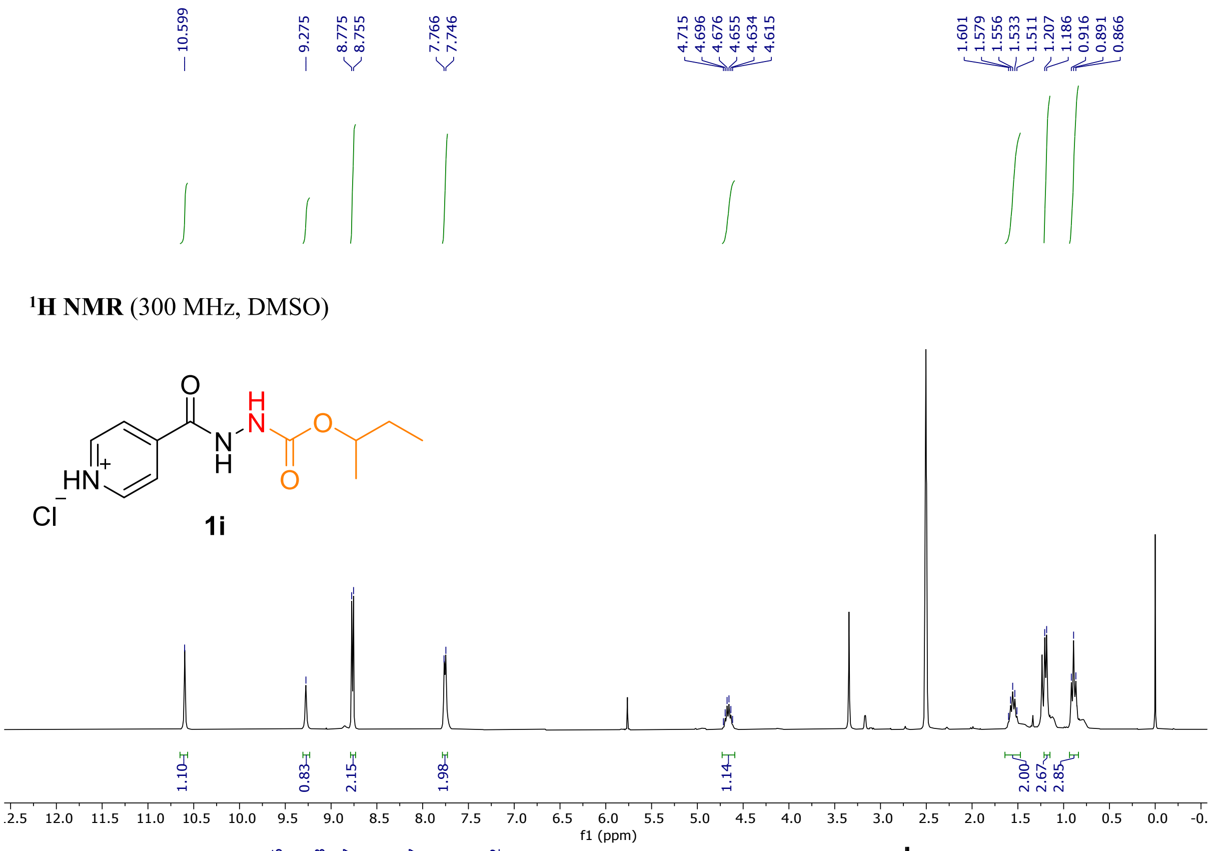

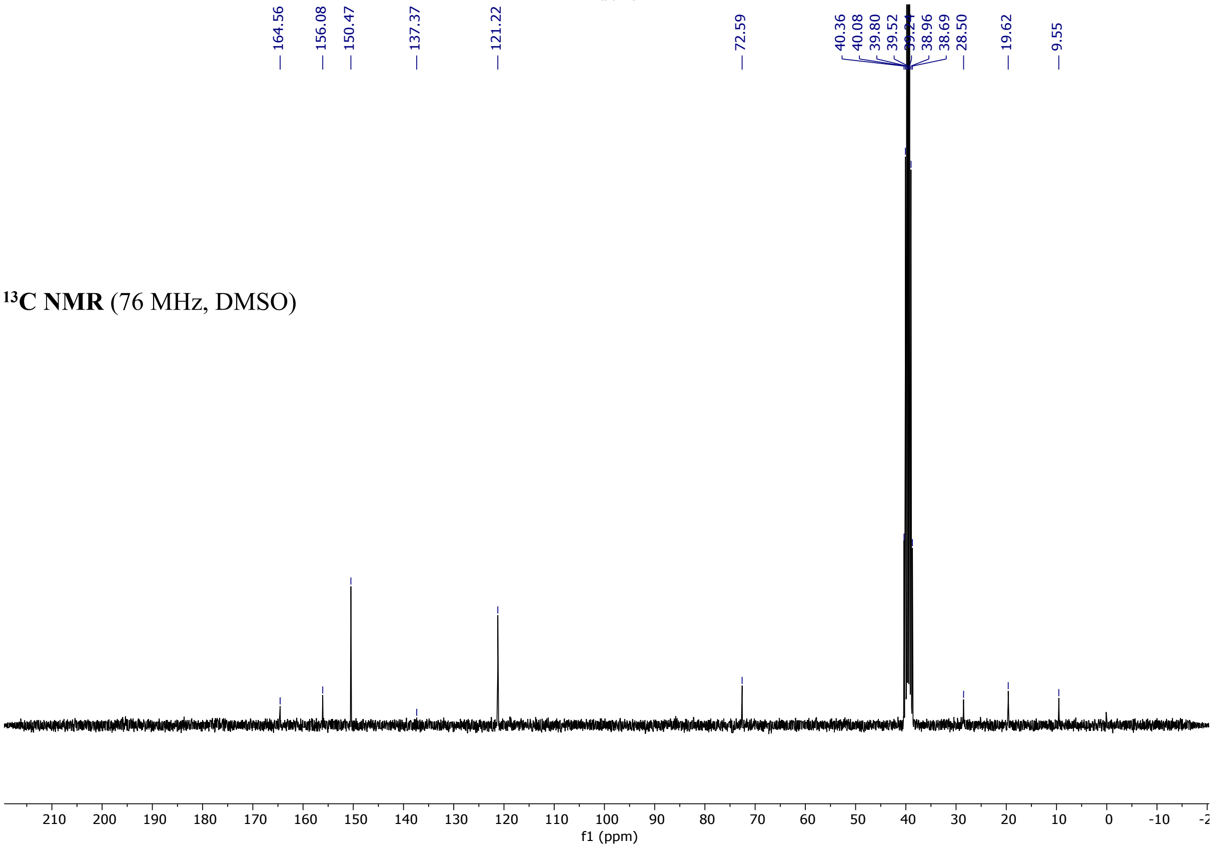

Figure S25. ^{1}H NMR and ^{13}C NMR of Prodrug **1i**.

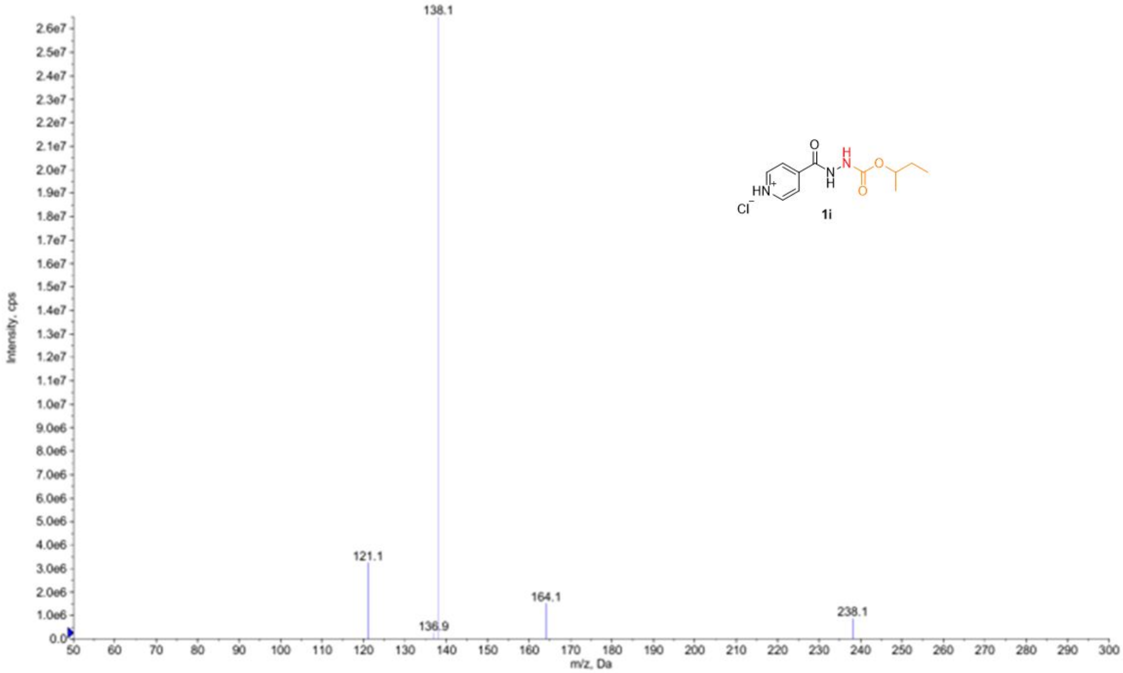

Figure S26. Mass spectra of **1i**

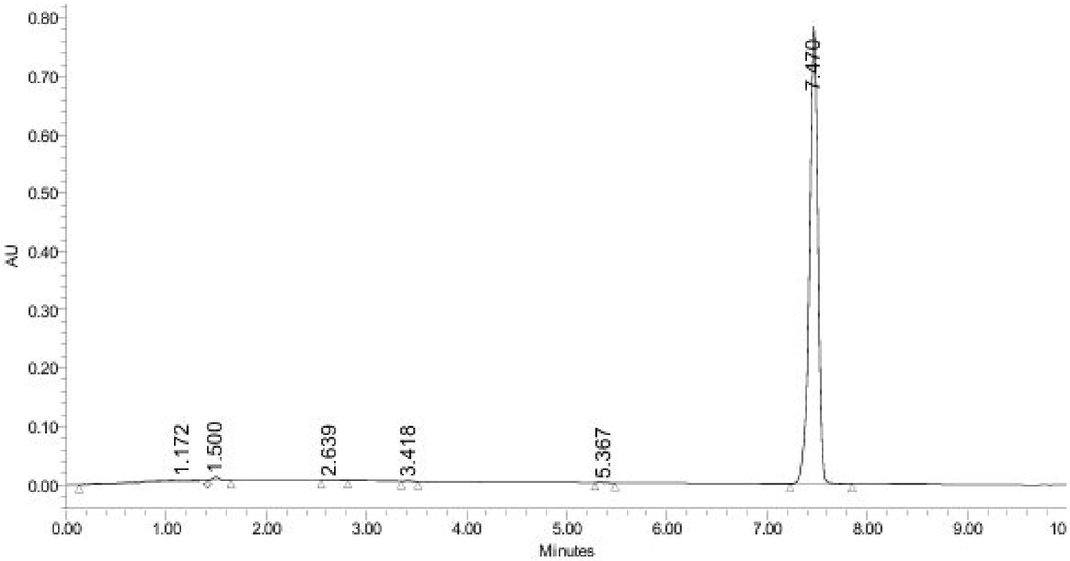

	RT	Area	% Area	Height
1	1.172	116537	2.43	3445
2	1.500	29317	0.61	6146
3	2.639	4080	0.09	642
4	3.418	5674	0.12	1290
5	5.367	2997	0.06	640
6	7.470	4635634	96.69	778647

Figure S27. HPLC Chromatogram of **1i**

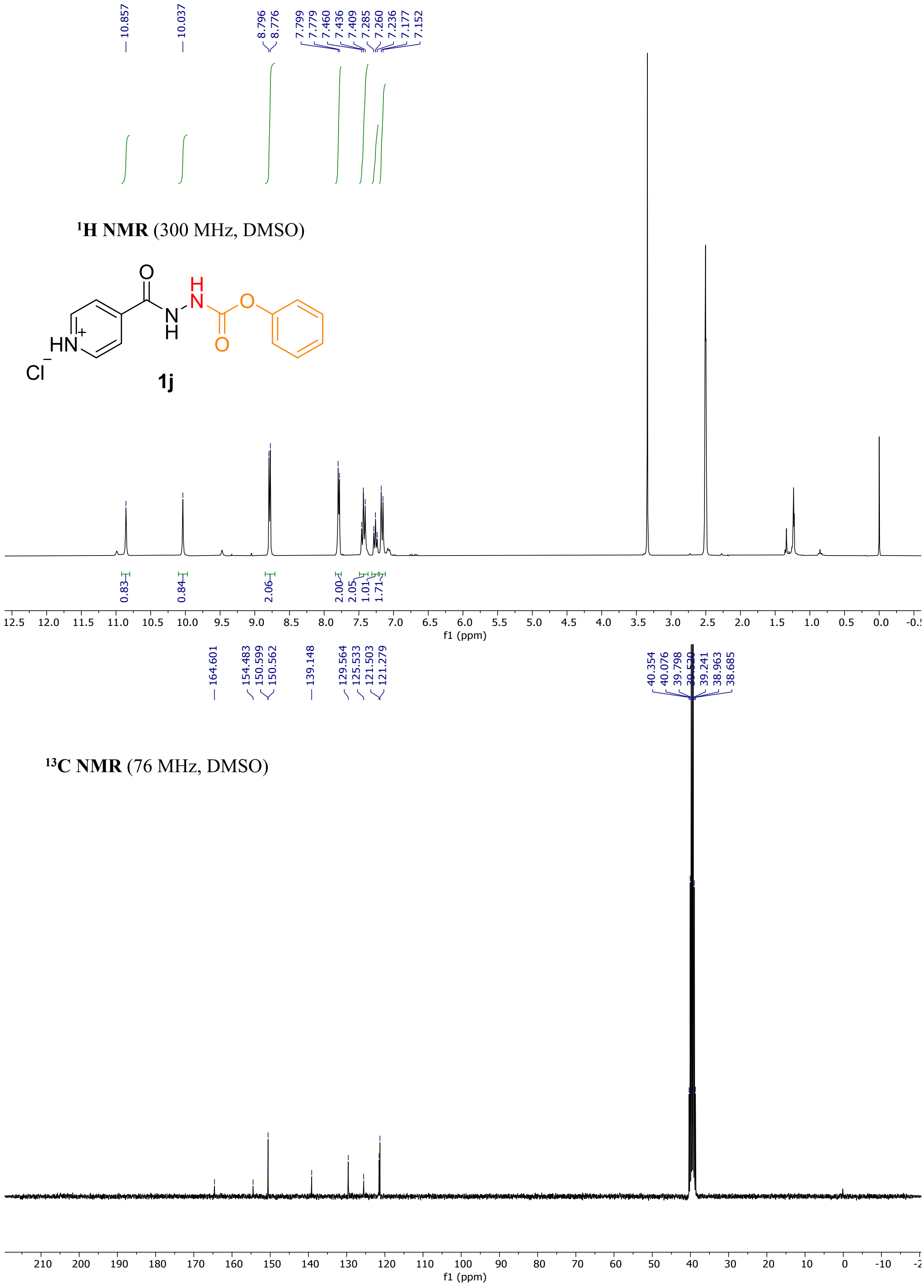

Figure S28. ^{1}H NMR and ^{13}C NMR of Prodrug **1j**.

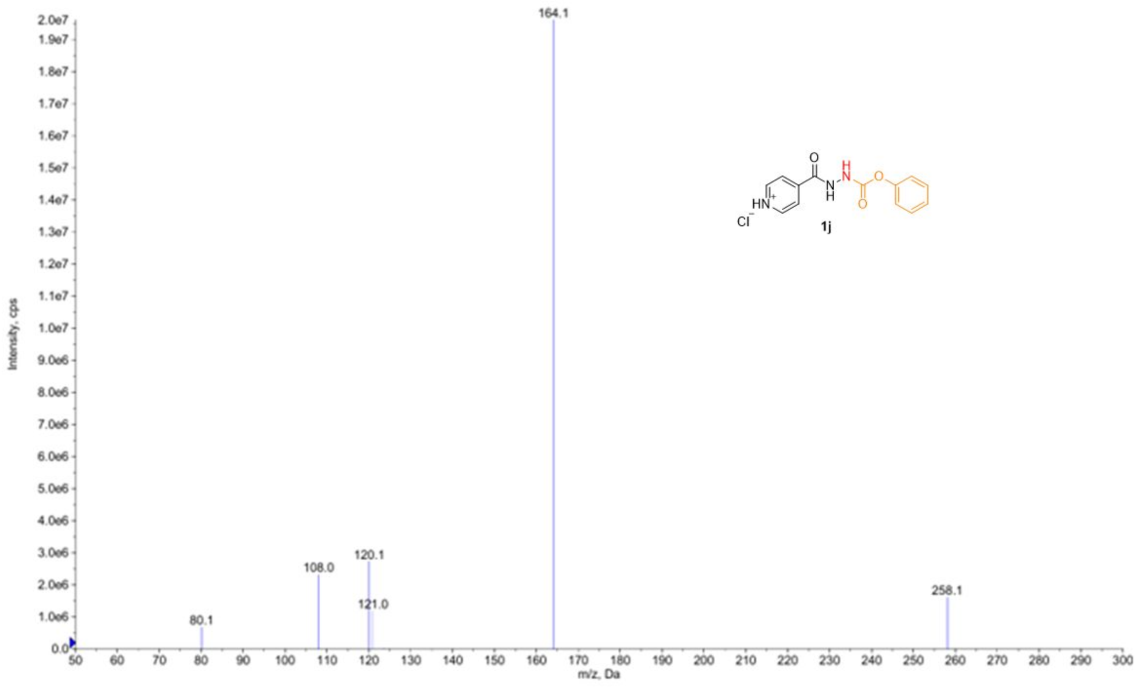

Figure S29. Mass spectra of **1j**

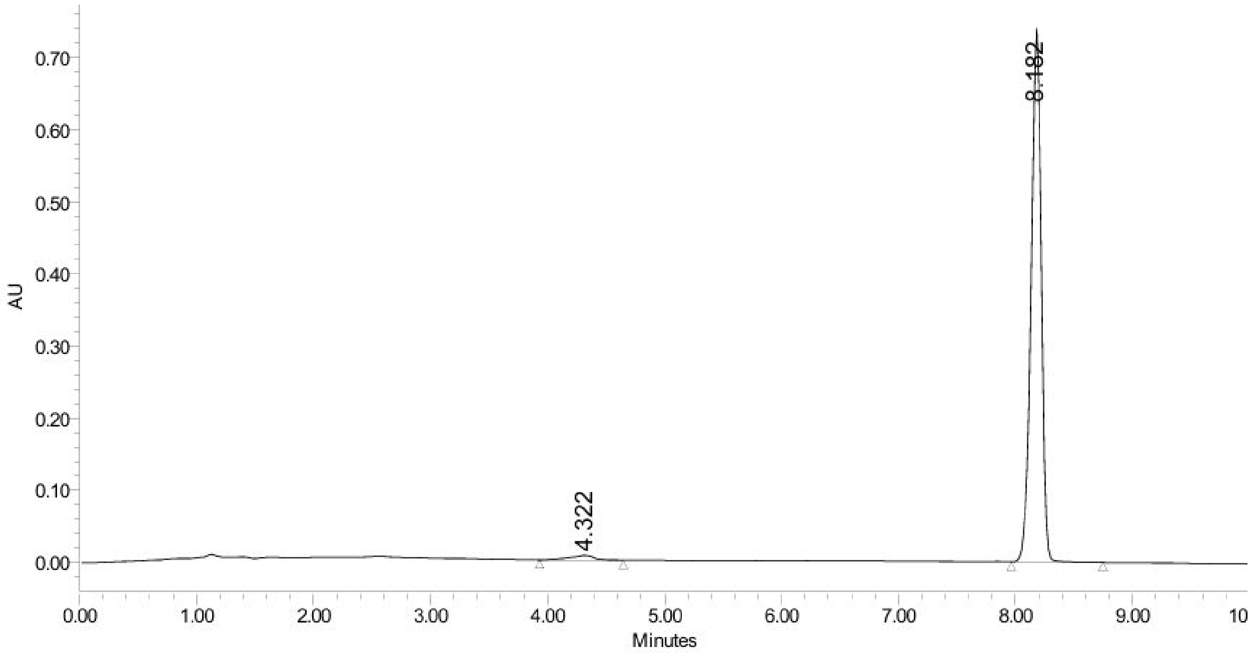

	RT	Area	% Area	Height
1	4.322	87776	1.93	6295
2	8.182	4468895	98.07	733956

Figure S30. HPLC Chromatogram of **1j**

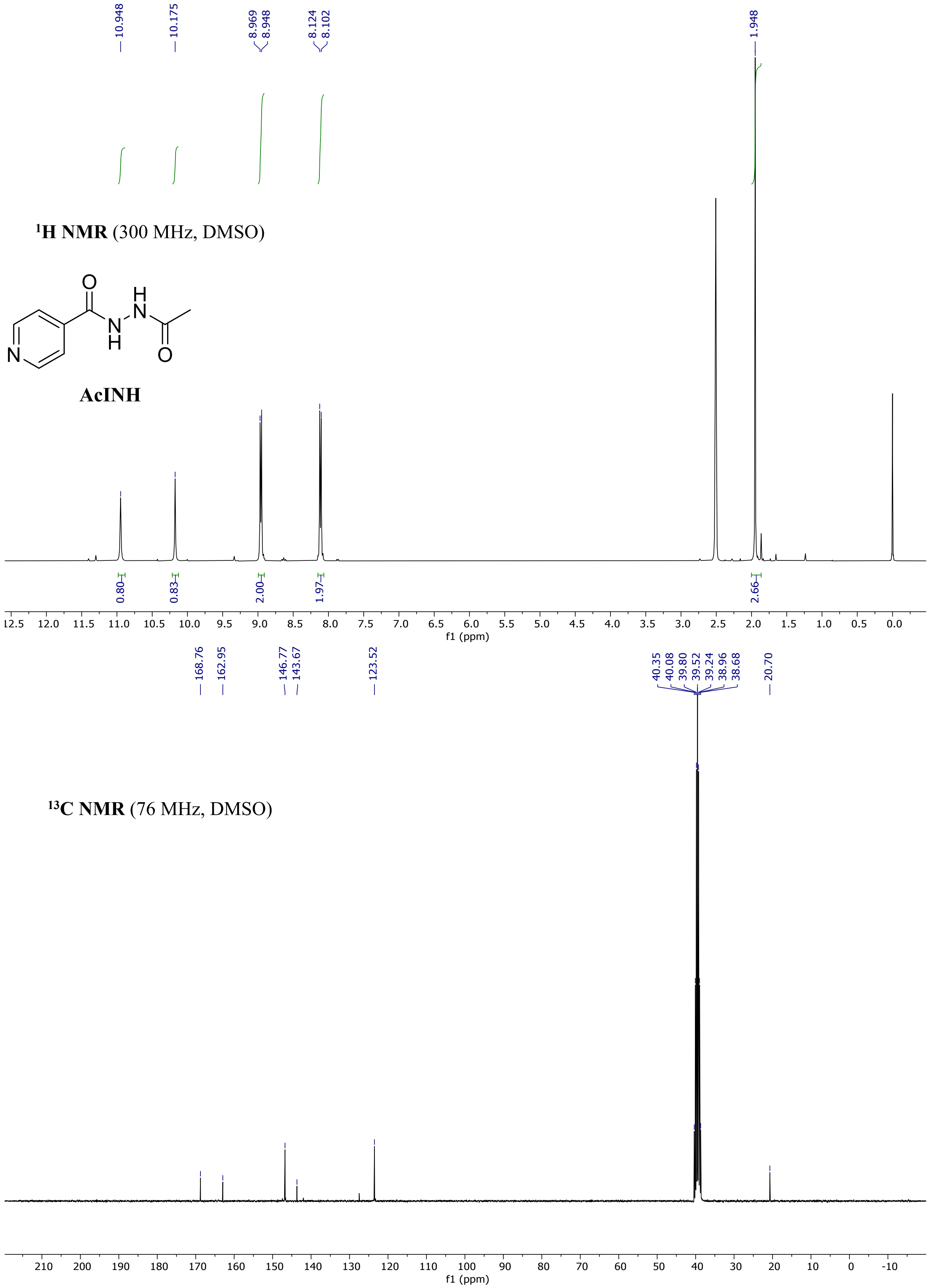

Figure S31. ^{1}H NMR and ^{13}C NMR of **AcINH**.

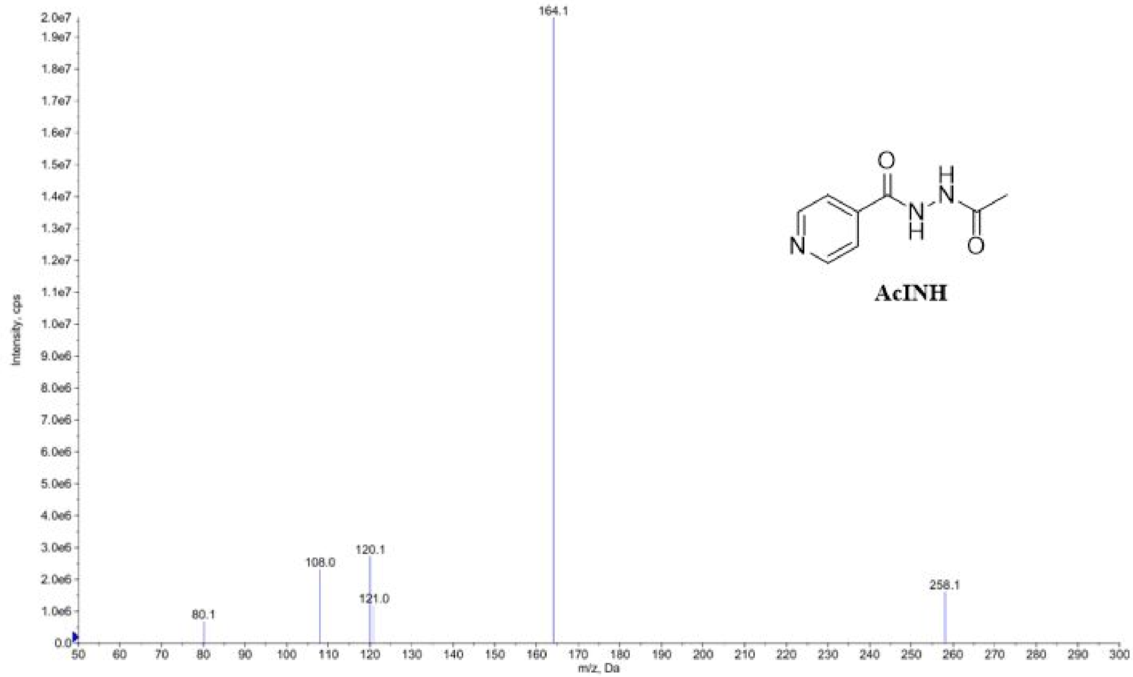

Figure S32. Mass spectra of **AcINH**

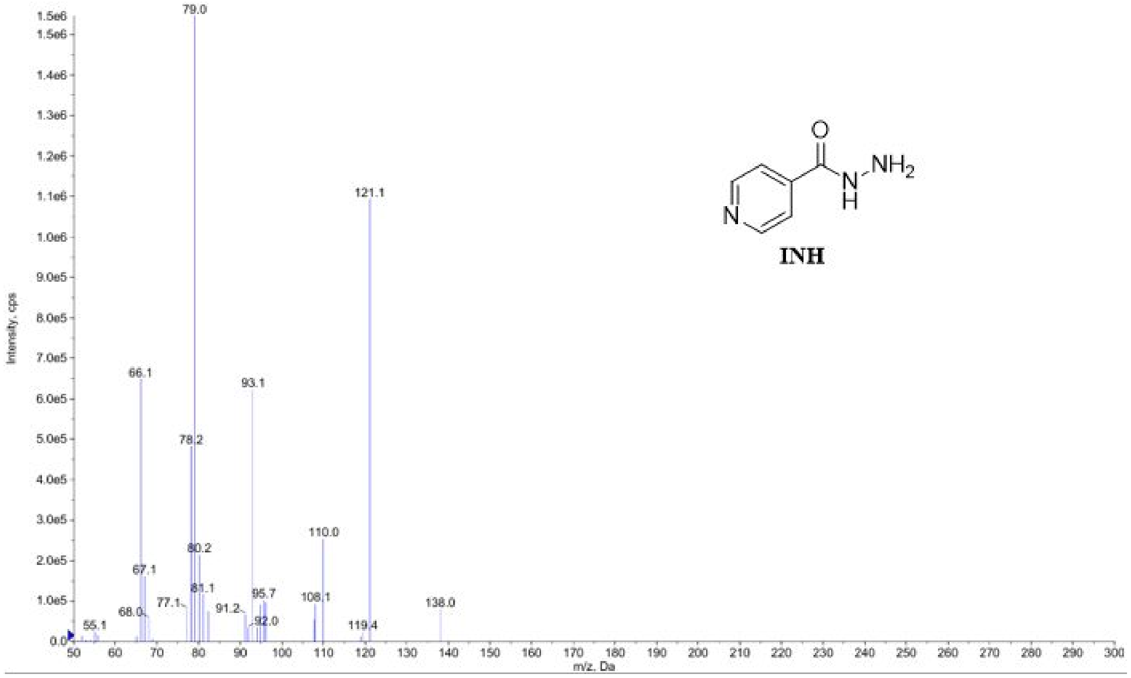

Figure S33. Mass spectra of **INH**

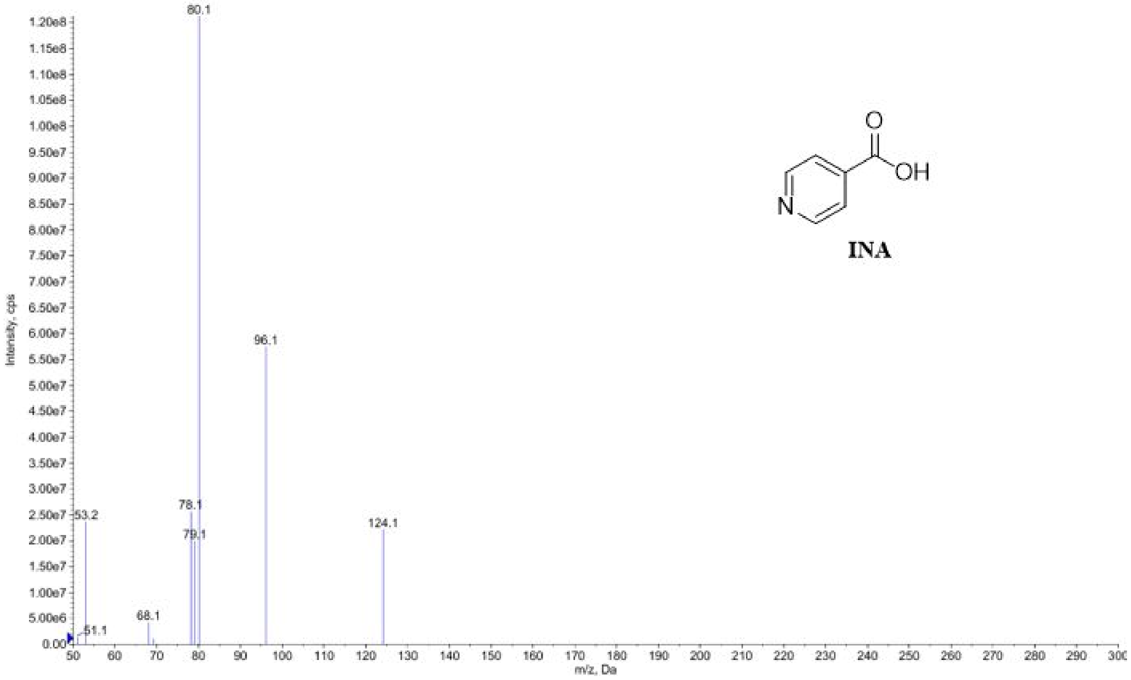

Figure S34. Mass spectra of **INA**

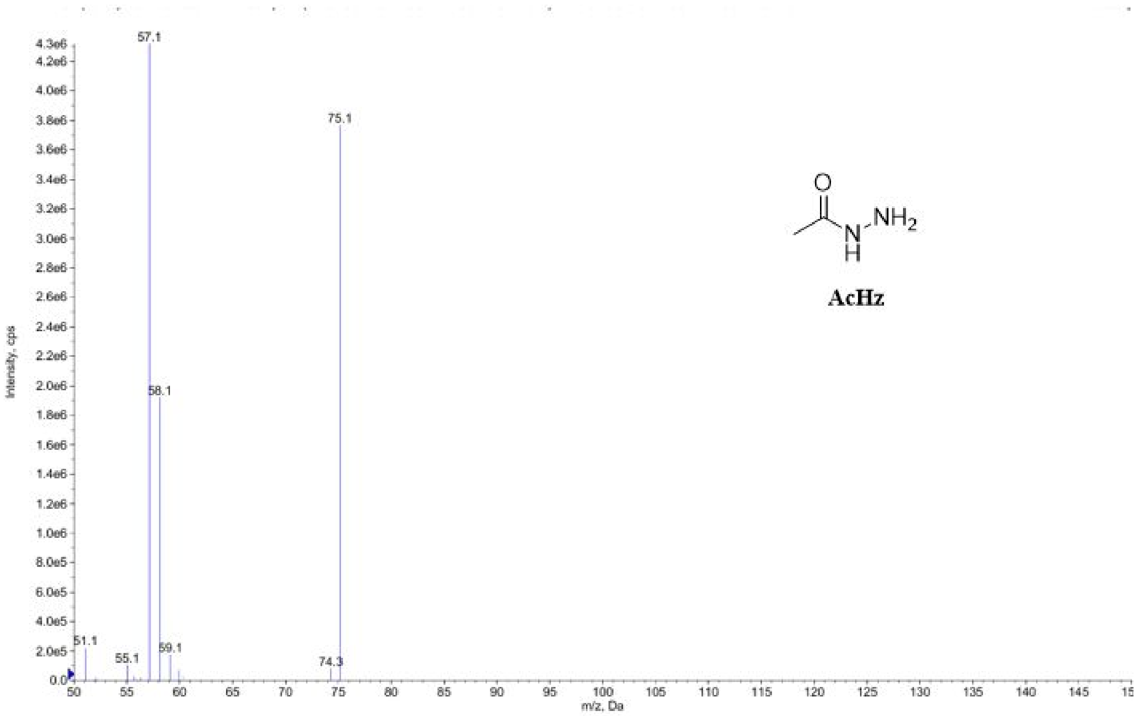

Figure S35. Mass spectra of **AcHz**

^{1}H NMR (300 MHz, DMSO)

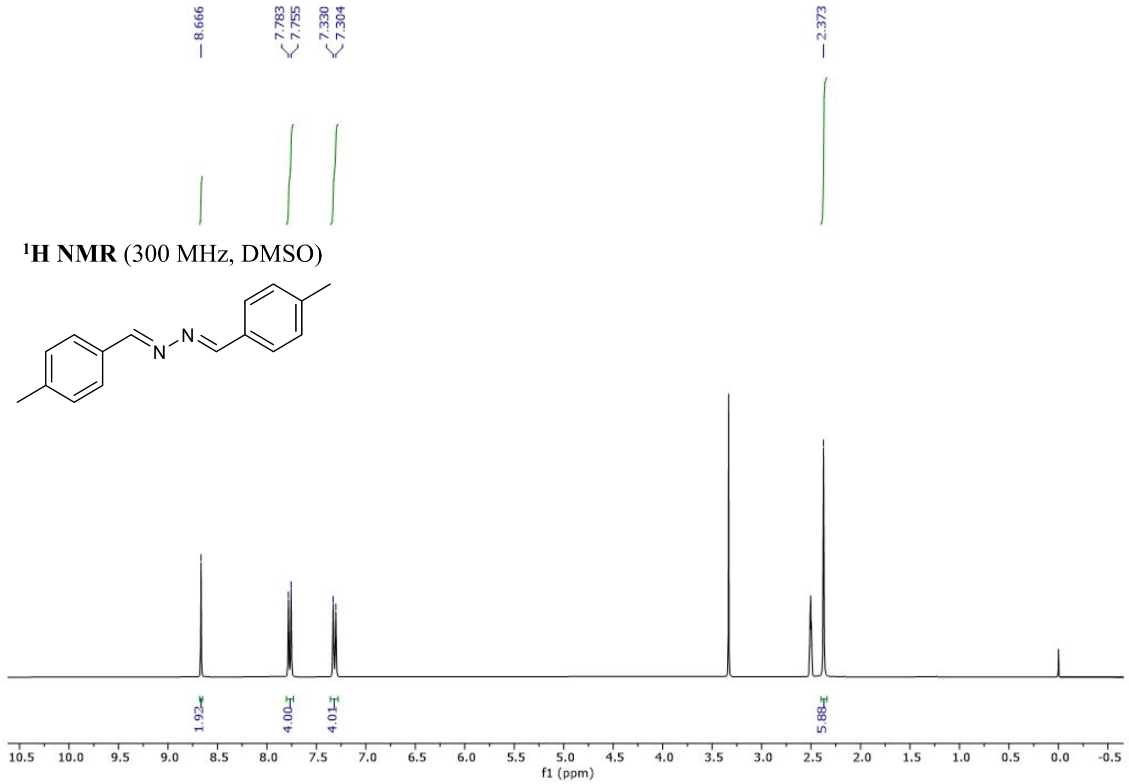

^{13}C NMR (76 MHz, DMSO)

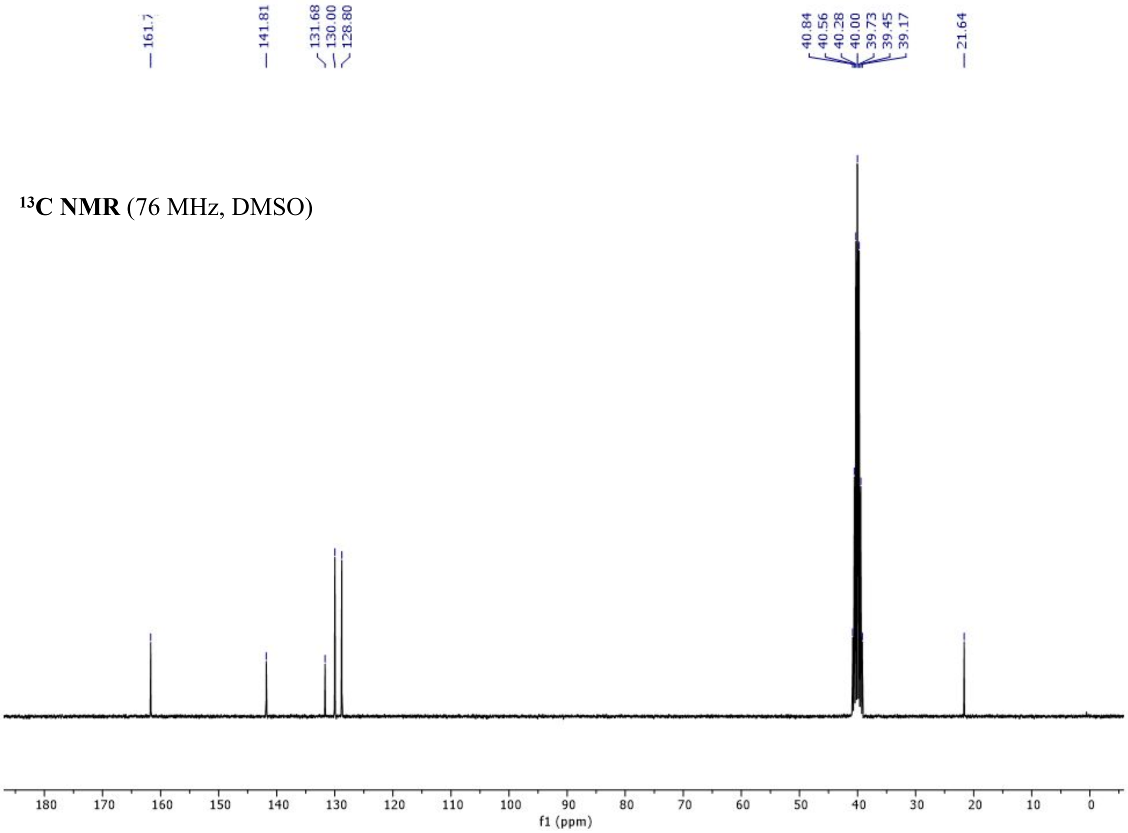

Figure S36. ^{1}H NMR and ^{13}C NMR of 1,2-bis((E)-4-methylbenzylidene)hydrazine (Derivatized form of hydrazine using p-tolualdehyde)

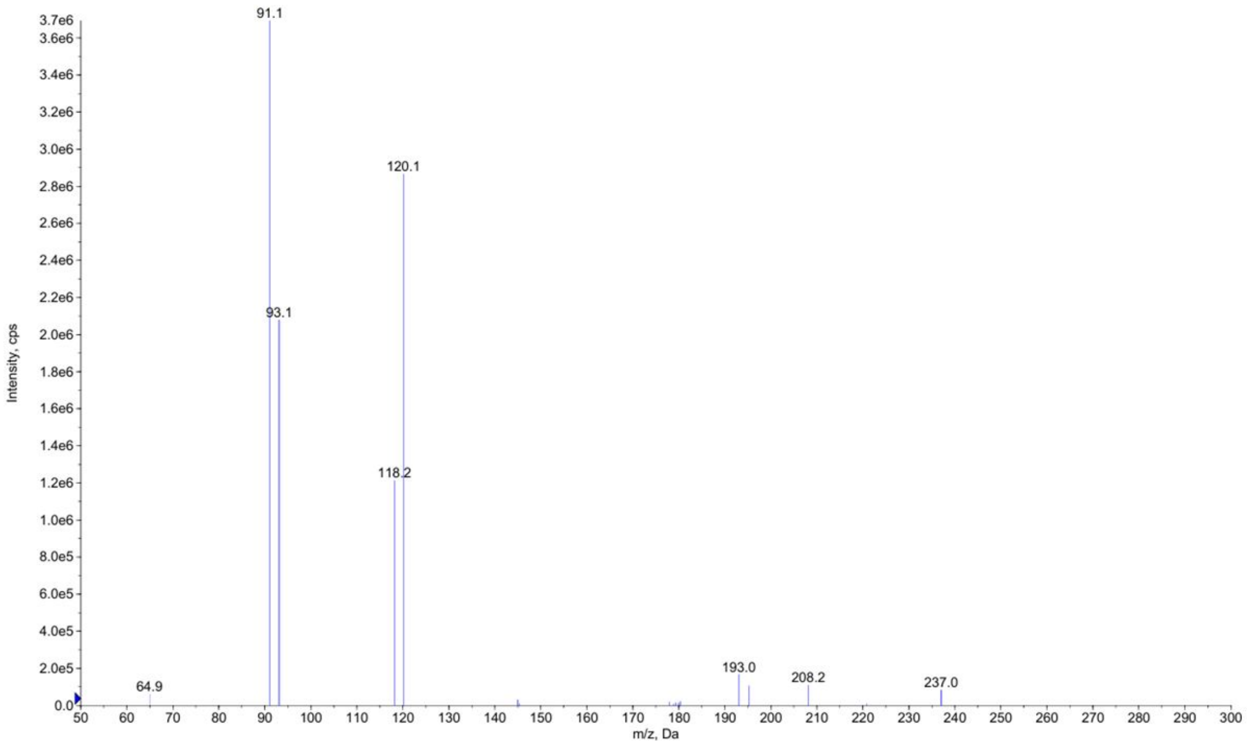

Figure S37. Mass spectra of 1,2-bis((E)-4-methylbenzylidene)hydrazine (Derivatized form of hydrazine using p-tolualdehyde)

LCMS based Bioanalytical Method Validation

All the parameters were assessed in five replicates. Mice plasma samples from five different matrix sources were used for each analysis.

Selectivity, linearity, carry over: All the samples were processed in five replicates from different matrix sources at a concentration level of LLOQ of each analyte. The signal responses at the retention time of four analytes from spiked samples were compared with blank plasma samples, for evaluating the interference of noise peaks with desired analyte peaks. The interferences between analyte signals and signals in blank plasma samples at corresponding analyte retention time were found to be less than 20%, which is in acceptable limits. The linearity of the analytes in this method was determined by calibration analysis by spiking each analyte in mice plasma at different concentrations and resulted in acceptable linear regression coefficient values (R^2), 0.9965, 0.9971, 0.9975 and 0.9873 for INH, AcINH, INA and AcHz, respectively. The carryover of analytes at its LLOQ level in this method reported approximately 3%-14.2%.

Precision and accuracy: Precision and accuracy of the method was evaluated at four different concentration levels (LLOQ, LQC, MQC and HQC; n=5/ for each concentration). Precision is measured as percentage of coefficient of variation (%CV) and accuracy is measured as percentage of relative error (%RE). The % of CV between replicate samples at LQC, MQC and HQC levels ranged across 1.25% - 14.31% whereas percentage of coefficient of variation values at LLOQ level reported approximately 3.64% - 20.48%. The intraday and inter-day accuracy reported across 91.24% - 114.63% and 80.86% - 114.31%, respectively, which were in acceptable limits.

Matrix effect and recovery: Pre-spiked plasma samples (n=4) with four analytes at the concentration level of LQC, MQC and HQC were processed and compared with blank plasma samples post-spiked with each analyte at the corresponding concentration level. All the analytes were found to have a mean extraction recovery from plasma in the average range of 79.2% - 113.2%. Plasma samples (n=5) pre-spiked with four analytes at the concentration level of LQC and HQC were processed and compared with standard aqueous samples at the corresponding concentration level to determine MF values.

%CV of the MF values of all the analytes were observed to be in the acceptable limits, <15%. Although, a limited ion suppression was observed as indicated by the lower MF value, but all the analytes exhibited significant recovery.

Stability: Stability of all four analytes in autosampler, 4°C storage and benchtop/room temperature were determined. All the analytes found to be stable in all the three tested conditions over a period of 24h, which indicates that samples may be processed within 24 hours if they are stored in these conditions. Stability in autosampler and stability in 4°C storage were analyzed together and ranged across -12.4% to 11.1%. The stability analysis in room temperature reported the accuracy of area/ concentration of analytes post 24 hrs in a range of -8.5% to 11.5%.

Table S1. Mass parameters of prodrugs 1a-1j

Compound	m/z	DP	CE	LLOQ (ng/mL)	Cal. Range (ng/mL)	Correlation coefficient
**1a**	196.200/164.200	45.00	20.00	0.024	0.024 – 100	0.9989
**1b**	210.200/164.200	55.00	25.00	0.024	0.024 – 100	0.9995
**1c**	224.300/164.100	45.00	23.00	0.024	0.024 – 25	0.9998
**1d**	224.300/138.200	50.00	23.00	0.024	0.024 – 50	1
**1e**	238.300/164.100	55.00	25.00	0.024	0.024 – 100	0.9986
**1f**	238.300/138.100	70.00	23.00	0.024	0.244 – 125	0.9987
**1g**	222.300/164.200	45.00	22.00	0.024	0.024 – 100	0.9996
**1h**	272.300/137.200	50.00	21.00	0.024	0.024 – 100	0.9939
**1i**	238.200/138.100	40.00	22.00	0.5	0.5 – 200	0.9977
**1j**	258.100/164.100	50.00	24.00	0.5	0.5 – 200	0.9970

Results of parameters evaluated for validation of the developed method for simultaneous quantification of INH, AcINH, INA and AcHz.

1. Linearity

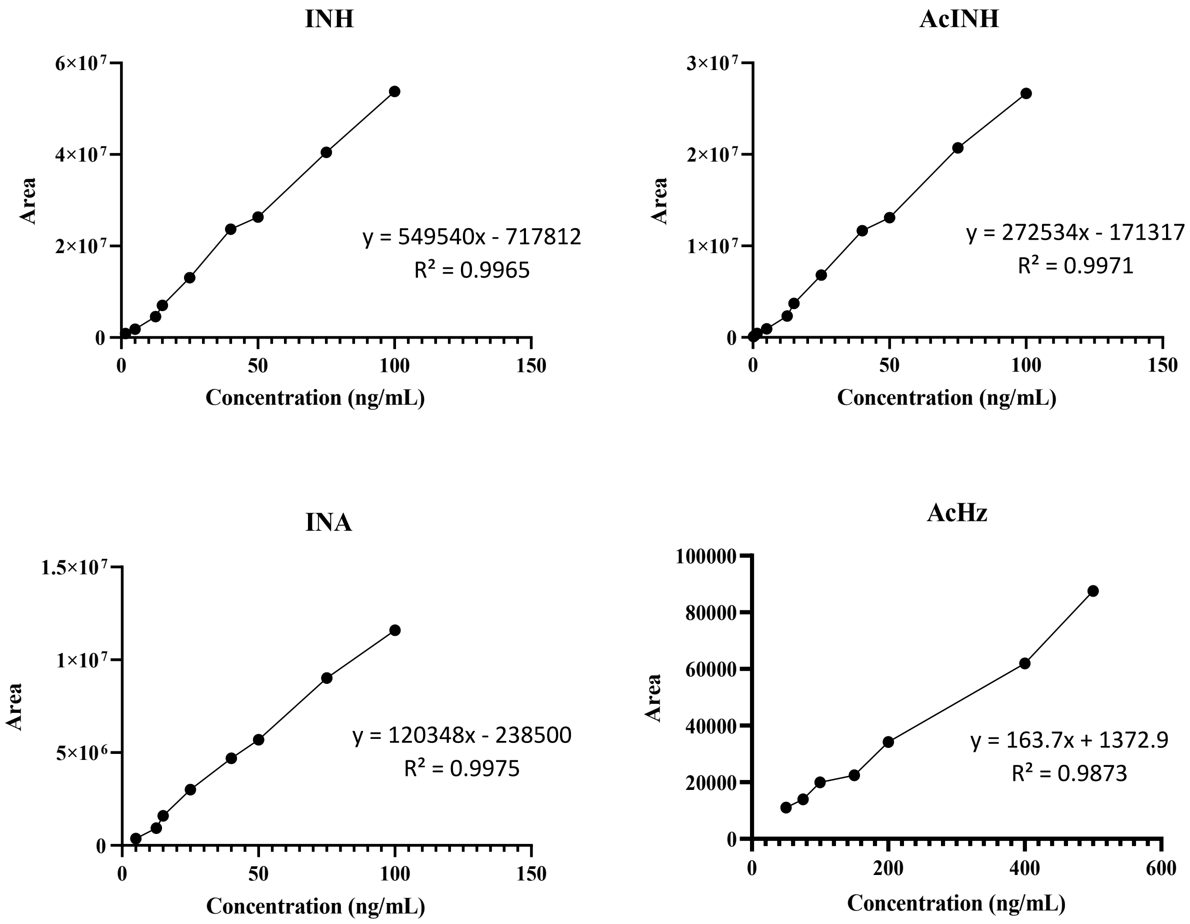

Figure S38. Linearity of trueness of back-calculated results for INH and metabolites in mice plasma

2. Selectivity

Table S2. Summarized results of selectivity evaluation					
Parameter	Concentration level	INH	AcINH	INA	AcHz
Selectivity	LLOQ	3.540± 1.139	4.263± 3.723	5.225± 1.398	9.100± 4.850

Table S3. Detailed results for selectivity evaluation											
INH			AcINH			INA			AcHz		
BL	LLOQ	%	BL	LLOQ	%	BL	LLOQ		BL	LLOQ	
15635.58	685936.1	2.279451	0	54435.76	0	31666.91	604762.7	5.236254	563.8247	10812.37	5.214627
13641.79	539235.3	2.52984	2364.403	60869.98	3.884351	37746.2	490499.6	7.695459	532.4685	9229.718	5.769066
16931.82	493615.5	3.430163	3846.076	42466.57	9.056714	17173.72	407544.4	4.213952	802.5758	7778.864	10.31739
37256.23	693453.7	5.372562	4784.938	87230.91	5.485369	33083.73	603033.7	5.486216	467.3447	12006.51	3.892429
20855.58	614739.1	3.39259	0	61118.67	0	24421.1	673999.9	3.623309	1676.914	11301.9	14.83745
18134.09	428297.7	4.233992	3452.947	48289.62	7.150494	20674.35	405843.1	5.094174	1123.668	7713.573	14.56741

3. Determination of Limit of Detection (LOD) and Limit of Quantification (LOQ)

Table S4. Detailed results of LOD determination

INH - 0.073ng/mL			AcINH - 0.024ng/mL			INA - 0.073ng/mL			AcHz - 40ng/mL		
Blank	LLOQ	LLOQ/Blank	Blank	LLOQ	LLOQ/Blank	Blank	LLOQ	LLOQ/Blank	Blank	LLOQ	LLOQ/Blank
9634	40737	4.228461698	397	9156	23.06297229	1991	9963	5.004018081	572	7200	12.58741259
8918	40737	4.567952456	1157	9156	7.913569576	1547	9963	6.440206852	954	7200	11.60587002
9808	40737	4.153446166	2320	9156	3.946551724	2199	9963	4.530695771	1136	7200	6.338028169
1243	40737	32.77312953	1220	9156	7.504918033	2490	9963	4.001204819	311	7200	35.60128617
7316	40737	5.56820667	2215	9156	4.133634312	1911	9963	5.213500785	1881	7200	3.827751196
3189	40737	12.77422389	1343	9156	6.817572599	1326	9963	7.513574661	2444	7200	2.94599018

Table S5. Detailed results of LOQ determination

INH - 1.5625ng/mL			AcINH - 0.195ng/mL			INA - 5ng/mL			AcHz - 50ng/mL		
Blank	LLOQ	LLOQ/Blank	Blank	LLOQ	LLOQ/Blank	Blank	LLOQ	LLOQ/Blank	Blank	LLOQ	LLOQ/Blank
9634	879632	91.30496159	397	75316	189.7128463	1991	366893	184.2757408	572	11072	19.35664336
8918	879632	98.63556851	1157	75316	65.09593777	1547	366893	237.1641888	954	11072	11.60587002
9808	879632	89.68515498	2320	75316	32.4637931	2199	366893	166.8453843	1136	11072	9.746478873
1243	879632	707.6685438	1220	75316	61.73442623	2490	366893	147.3465863	311	11072	35.60128617
7316	879632	120.2340077	2215	75316	34.0027088	1911	366893	191.9900576	1881	11072	5.886230728
3189	879632	275.8331765	1343	75316	56.08041698	7092	366893	51.73336153	2444	11072	4.530278232

4. Stability in auto sampler conditions (5^0C)

Table S6. Summarized results for stability evaluation under auto-sampler conditions

	INH	AcINH	INA	AcHz
LQC	-1.53766	-1.10054	-1.80925	-8.50053
MQC	-2.72652	11.10426	5.186026	-12.4278
HQC	7.760427	9.243727	1.535597	0.381292

Table S7. Detailed results of stability evaluation under auto sampler conditions

	LQC				MQC				HQC			
	INH	AcINH	INA	AcHz	INH	AcINH	INA	AcHz	INH	AcINH	INA	AcHz
Sample 1	2815788	235481.4	76206.38	18569.55	34656893	15053101	6236962	23615.84	57956613		10490540	33060.48
Sample 2	2643937	218207	80419.55	17256.78	33988393	14727838	5948151	21210.58	51451391	21292496	8701081	31501.74
Sample 3	2698634	222963.2	59590.1	17945.38	35184534	15345502	6335313	18878.91	54214107	22231819	9282250	32582.96
Sample 4	2823737	234339.9	79501.54	16541.61	34524995	14826316	6112063	22387.66	50964786	21775246	8734028	29160.73
Sample 5	2579940	227798.2	62973.98	14607.49	34230382	14794885	6031018	20874.43	41829199	18983237	7190743	25191.18
Mean	2712407	227757.9	71738.31	16984.16	34517040	14949528	6132701	21393.48	51283219	21070699	8879728	30299.42
Nominal value	2754766	230292.4	73060.15	18562.03	35484532	13455405	5830339	24429.53	47590030	19287789	8745434	30184.33
Accuracy	-1.53766	-1.10054	-1.80925	-8.50053	-2.72652	11.10426	5.186026	-12.4278	7.760427	9.243727	1.535597	0.381292

5. Stability on bench top

Table S8. Summarized results of stability analysis on bench top conditions

	INH	AcINH	INA	AcHz
LQC	5.956204	5.564415	-8.57079	8.179407
MQC	-0.91081	11.70655	2.201084	2.804306
HQC	7.12161	16.69468	8.971655	17.51314

Table S9. Detailed results of stability analysis on bench top conditions

	LQC				MQC				HQC			
	INH	AcINH	INA	AcHz	INH	AcINH	INA	AcHz	INH	AcINH	INA	AcHz
Sample 1	2666436	253446.8	79519.67	19327.28	35285918	15282581	6011870	25231.78	55276283	22821158	10147253	40544.8
Sample 2	3299988	279436	67221.66	22337.83	39967903	17170343	6783471	29082.61	47142183	22471412	10481407	31093.94
Sample 3	3175974	221256.9	68211.78	18315.66	34646354	14645632	5764243	25132.28	45745110	20145468	8018644	32039.7
Sample 4	2532985	218287.7	52240.17	20340.42	30745171	13023717	5275092	21011.77	55753247	24593253	9472872	38203.75
Mean	2918846	243106.8	66798.32	20080.3	35161337	15030568	5958669	25114.61	50979206	22507823	9530044	35470.55
Nominal value	2754766	230292.4	73060.15	18562.03	35484532	13455405	5830339	24429.53	47590030	19287789	8745434	30184.33
Accuracy	5.956204	5.564415	-8.57079	8.179407	-0.91081	11.70655	2.201084	2.804306	7.12161	16.69468	8.971655	17.51314

6. Matrix effect

Table S10. Summarized results of matrix effect evaluation

	INH	AcINH	INA	AcHz
LQC	9.650598	11.75292	10.26295	13.47248
HQC	6.044085	6.387001	6.517086	6.310674

Table S11. Detailed results of matrix effect evaluation

	LQC				HQC			
	INH	AcINH	INA	AcHz	INH	AcINH	INA	AcHz
Sample 1	0.482303	0.615372	0.635921	0.616145	0.50998	0.56235	0.644481	0.598829
Sample 2	0.445792	0.530729	0.570678	0.57062	0.557472	0.607981	0.700559	0.630055
Sample 3	0.475676	0.574659	0.618152	0.529238	0.619919	0.673762	0.766306	0.7024
Sample 4	0.555149	0.646785	0.718859	0.742024	0.595199	0.660369	0.74632	0.69406
Sample 5	0.588122	0.738464	0.771131	0.773347	0.590996	0.664481	0.757387	0.656714
Sample 6	0.534732	0.725126	0.717259	0.657398	0.583122	0.66835	0.787343	0.717135
SD	0.049568	0.075045	0.068967	0.087319	0.034821	0.040848	0.047818	0.042063
CV	0.513629	0.638523	0.672	0.648129	0.576115	0.639549	0.733733	0.666532
% of CV	9.650598	11.75292	10.26295	13.47248	6.044085	6.387001	6.517086	6.310674

7. Recovery analysis

Table S12. Summarized results of recovery evaluation

Parameter	Concentration level	INH	AcINH	INA	AcHz
Recovery	LQC	86.178±5.398	81.210± 0.555	85.252±4.404	88.778± 5.976
	MQC	97.020± 7.041	94.499± 8.166	85.901±3.871	100.165± 9.971
	HQC	101.850± 8.538	111.009± 5.993	90.400±5.187	90.542±8.949

Table S13. Detailed results of recovery evaluation

	INH			AcINH			INA			AcHz		
	Extracted	Post spiked	Recovery(%)	Extracted	Post spiked	Recovery(%)	Extracted	Post spiked	Recovery(%)	Extracted	Post spiked	Recovery(%)
LQC	1395643	1529538	91.24602	172774.2	212924.1	81.14355	882796.6	1101362	80.15497	16392.84	20359.71	80.51605
	1246092	1470868	84.71814	157948.8	192650.4	81.98726	879982.2	1033439	85.15083	17014.42	19252.7	88.37424
	1188982	1500835	79.2214	160147.9	198498.2	80.67978	901422.7	1063072	84.79415	17606	19064.27	92.35077
	1634512	1825749	89.5256	186951.3	230724.1	81.02807	1148746	1263625	90.90877	21703.58	23120.78	93.87044
MQC	15251903	15400975	99.03206	7990703	8036245	99.43329	2865283	3224381	88.86303	26325.63	29198.09	90.16218
	13429094	14594578	92.01427	6836401	7790100	87.75754	2568231	3108202	82.62755	25902.42	27219.87	95.15996
	13763747	15136618	90.93014	7038282	8054547	87.38272	2627875	3185590	82.49258	26282.26	25724.22	102.1693
	16936851	15962483	106.1041	8778125	8487701	103.4217	3075244	3431325	89.62266	32836.25	29015.04	113.1697
HQC	29002507	25614932	113.225	14196712	11892986	119.3705	5382076	5614861	95.85412	52093.73	50800.35	102.546
	28100795	27967039	100.4783	14202308	12983822	109.3847	5630906	6004831	93.77292	50474.86	54764.35	92.16736
	28785401	31110773	92.52551	14924233	14194681	105.1396	5692329	6663346	85.42749	49668.94	58923.59	84.29382
	30717446	30361734	101.1716	15466410	14042136	110.1429	5740289	6632610	86.54647	50757.86	61034.98	83.16192

8. Carry over

Table S14. Summarized results of carry over evaluation				
Concentration level	INH	AcINH	INA	AcHz
LQC	3.659± 0.477	5.550± 1.451	5.528±1.855	8.927± 4.080

Table S15. Detailed results of carry over evaluation

INH				AcINH				INA				AcHz			
UL	BL	LL	%	UL	BL	LL	%	UL	BL	LL	%	UL	BL	LL	%
48097688	26278	682757	3.85	24346344	4986	64152	7.77	10554692	45711	628627	7.27	75209	988	12385	7.97
41512998	17077	510867	3.34	21680776	3229	56682	5.70	9257274	41237	541184	7.62	69648	1251	8834	14.16
53378312	20324	488571	4.16	27391617	3110	56120	5.54	12134225	15319	452669	3.38	87509	974	8171	11.92
50513890	24454	816490	3.00	25098153	3199	64600	4.95	11203281	28562	666776	4.28	82852	439	10676	4.11
49459458	18752	474944	3.95	25392890	1840	48565	3.79	10989768	22542	443846	5.08	83132	530	8199	6.47

9. Precision and Accuracy

Table S16. Detailed results of precision analysis – Intraday 1

**Concentration level**	**LLOQ**				**LQC**			
**Replicate Values**	INH	AcINH	INA	AcHz	INH	AcINH	INA	AcHz
	406107.1066	39293.9	348997.4	7391.15	2708792	219462.9	46135.13	17614.45
	384799.6337	34801.53	304601.5	5866.129	3117099	267642.9	48791.23	22250.6
	598522.2179	51407.84	415226.4	8357.265	2969332	254540.3	45766.2	21525.77
	*696207.2754**	34525.06	290880.5	8021.404	2555781	208853.8	40823.62	16261.81
	405209.1171	33316.39	*635452.4**	6593.028	2600473	204010.1	40608.86	19118.68
**SD**	86941.87525	6685.037	48491.55	915.1662	217498.2	25490.45	3203.754	2269.358
**CV**	448659.5188	38668.95	339926.4	7245.795	2790295	230902	44425.01	19354.26
**% of CV**	19.37814124	17.28787	14.26531	12.63031	7.794809	11.03951	7.211599	11.72537
**Concentration level**	**MQC**				**HQC**			
**Replicate Values**	INH	AcINH	INA	AcHz	INH	AcINH	INA	AcHz
	17124078.05	8704032	3491431	22772.15	34950139	17549319	7121436	56243.3
	16508781.04	8110561	2983361	27915.22	29505045	14423242	5654805	46445.62
	16692426.41	8661707	3309293	28807.74	25283633	15460471	5933983	51220.99
	15185756.44	7912390	2966133	24993.5	30750816	15242941	6040189	48576.82
	16730016.57	8360420	3221317	28598.43	30152275	16607950	6480353	59764.73
**SD**	662315.9469	307064.2	199396.7	2359.213	3082125	1097072	511963.7	4908.339
**CV**	16448211.7	8349822	3194307	26617.41	30128382	15856784	6246153	52450.29
**% of CV**	4.026674503	3.677494	6.242253	8.863421	10.22997	6.918628	8.196464	9.358077

*Excluded from calculations

Table S17. Detailed results of precision analysis – Intraday 2								
**Concentration level**	**LLOQ**				**LQC**			
**Replicate Values**	INH	AcINH	INA	AcHz	INH	AcINH	INA	AcHz
	467199.7646	65824.71	407738.3	7531.007	3572854	298178		24684.9
	468881.9011	34230.83	367869.9	7873.37	2816911	238535.2	69615.41	17575.23
	703700.0666	57156.24	494479.8	10386.65	2957293	229341.6	67532.49	19520.27
	481162.4179	54444.49	310896.6	7752.93	2608131	193549.6	54477.8	16561.65
	446642.8402	63033.23	371525	7248.255	2785009	209918.6	57851.45	18315.22
**SD**	95735.2071	11117.59	60527.4	1134.261	331599.1	35713.28	6361.043	2845.485
**CV**	513517.3981	54937.9	390501.9	8158.443	2948040	233904.6	62369.29	19331.45
**% of CV**	18.64303088	20.23665	15.4999	13.90291	11.24812	15.26831	10.199	14.71945
**Concentration level**	**MQC**				**HQC**			
**Replicate Values**	INH	AcINH	INA	AcHz	INH	AcINH	INA	AcHz
	17424740.7	8878564	3498148	30439.9	28858183	17167109	6797936	53507.73
	14252756.72	7688880	3043569	25994.91	25271305	14440650	5522019	44567.79
	17468401.24	8671074	3391725	30757.02	30934837	15661909	5995675	53130.89
	14947910.36	7885949	2951177	23671.39	29677759	14795630	5842361	50647.27
	16822124.84	8330542	3230074	26383.14	32365616	16810878	6516906	57969.88
**SD**	1330709.753	451191	205089.3	2735.297	2390193	1073634	461764.2	4387.551
**CV**	16183186.77	8291002	3222939	27449.27	29421540	15775235	6134979	51964.71
**% of CV**	8.222791791	5.441936	6.363425	9.964915	8.123958	6.805822	7.526745	8.443327

Table S18. Detailed results of precision analysis – Inter day 1								
**Concentration level**	**LLOQ**				**LQC**			
**Replicate Values**	INH	AcINH	INA	AcHz	INH	AcINH	INA	AcHz
	570349.8187	54287.01	481429.2	9526.549	2917502	251537.1	59714.91	18604.86
	557680.0551	60900.78	462086.4	7978.213	2666907	239292.1	59965.07	16251.41
	453701.3054	*25745.28**	356812.6	4950.18	2655412	229390.8	66257.53	16255.91
	302625.0141	48155.71	399392.1	8851.948	2919094	241565.3	71588.07	17001.18
	559510.1017	53369.82	336255.8	7516.715	2891075	230699.9	62034.19	17385.93
**SD**	102262.2057	4531.247	56842.64	1569.384	121987.2	8047.459	4497.633	870.4841
**CV**	488773.259	54178.33	407195.2	7764.721	2809998	238497	63911.95	17099.86
**% of CV**	20.92221778	8.363579	13.95956	20.21172	4.341186	3.374239	7.037233	5.090592
**Concentration level**	**MQC**				**HQC**			
**Replicate Values**	INH	AcINH	INA	AcHz	INH	AcINH	INA	AcHz
	20509720.85	9650244	3453981	31711.08	31982261	16303842	6247330	61845.74
	15730925.53	6535610	3140590	22275.49	25657683	16086517	4862922	44428.59
	14507385.91	8045583	2925482	27238.66	30859400	14899282	6120017	52543.52
	16706662.07	7507235	3344622	26212.64	29222831	16197500	5621800	53970.83
	13911146.09	8531080	2729515	23227.6	31773090	13330116	6287755	42861.75
**SD**	2329268.466	1037317	265605.7	3336.629	2333261	1136019	537844	6901.714
**CV**	16273168.09	8053950	3118838	26133.09	29899053	15363452	5827965	51130.09
**% of CV**	14.31355255	12.8796	8.516175	12.76783	7.803795	7.394299	9.228677	13.49834

*Excluded from calculations

Table S19. Detailed results of precision analysis – Inter day 2								
**Concentration level**	**LLOQ**				**LQC**			
**Replicate Values**	INH	AcINH	INA	AcHz	INH	AcINH	INA	AcHz
	393142.6938	37753.21	276359.5	4822.691	3289477	250627.8	53037.12	19913.23
	437033.9247	37910.14	308494.3	4785.057	3346213	257265.7	60777.97	19802.96
	351918.3352	33888.66	295844.9	3745.045	3096107	206830.7	53167.9	18792.57
	459107.5195	49130.51	246107.6	4303.328	2688256	209161.6	47910.18	16956.11
	352664.8095	*24733.32**	215692.8	3529.343	2777364	230356.1	49360.11	17088.5
**SD**	43490.63761	5694.215	33761.17	527.3705	265274.9	20671.27	4462.509	1277.174
**CV**	398773.4566	39670.63	268499.8	4237.093	3039483	230848.4	52850.66	18510.68
**% of CV**	10.90610142	14.35373	12.574	12.44652	8.727631	8.954481	8.443621	6.89966
**Concentration level**	**MQC**				**HQC**			
**Replicate Values**	INH	AcINH	INA	AcHz	INH	AcINH	INA	AcHz
	13336551.18	6816157	2403101	23404.33	24322908	11570926	4116068	42367
	12034025.08	6103595	2175343	21330.58	23765400	11545440	4352128	43035.63
	12237035.65	7431630	2173529	24301.5	24689540	12282063	4553801	41921.38
	9209309.084	4539201	1643002	15552.23	26263673	12863412	4563408	42262.31
	12234510.06	6143399	2241792	20700.37	22713731	11256041	3767625	40500.67
**SD**	1378891.353	966134.6	256189	3050.944	1165958	586984.9	299816.6	840.0806
**CV**	11810286.21	6206796	2127353	21057.8	24351050	11903576	4270606	42017.4
**% of CV**	11.6753424	15.56575	12.04262	14.48843	4.788123	4.931164	7.020469	1.999364

*Excluded from calculations

**Table S20. Detailed results of precision analysis – Inter day 3**								
**Concentration level**	**LLOQ**				**LQC**			
**Replicate Values**	INH	AcINH	INA	AcHz	INH	AcINH	INA	AcHz
	869025.7967	63913.37	33277.48	8776.621	1720592	197640.8	990666.3	18543.72
	979921.3125	64957.37	41998.65	9315.663	1675428	210015.9	1038628	18709.12
	945572.0987	59398.27	*13488.73**	7319.526	1663584	206638.8	1031591	17104.33
	774343.0304	50270.84	24544.49	9934.372	1687377	216213.7	971231	19677.73
	1112953.011	54258.72	38521.99	8274.946	1697909	189155.5	1064702	18481.04
**SD**	113093.7094	5612.467	6576.159	893.5357	19545.95	9519.518	33812.76	822.3007
**CV**	936363.0499	58559.71	34585.65	8724.226	1688978	203933	1019364	18503.19
**% of CV**	12.07797653	9.584178	19.01413	10.24201	1.157265	4.667965	3.317046	4.444103
**Concentration level**	**MQC**				**HQC**			
**Replicate Values**	INH	AcINH	INA	AcHz	INH	AcINH	INA	AcHz
	45645448.32	19674423	7861191	32396.77	56838215	26419291	10238054	34062.97
	34944041.71	13573186	5825749	25650.03	50363113	20077466	8670069	27807.89
	35969042.28	14101632	6230126	24992.4	50599541	20802827	8535585	32275.12
	35209011.45	13437337	6095462	25399.27	48898659	20668021	8344451	29318.65
	36022067.93	14760944	5985840	20967.27	38787560	17625594	6751575	23645.2
**SD**	4065494.296	2329472	742694.2	3678.996	5832855	2887390	1107420	3623.983
**CV**	37557922.34	15109504	6399674	25881.15	49097418	21118640	8507947	29421.96
**% of CV**	10.82459849	15.41726	11.60519	14.21496	11.88017	13.67223	13.0163	12.31727

*Excluded from calculations

Table S21. Detailed results of accuracy analysis – Intraday 1								
**Concentration level**	**LLOQ**				**LQC**			
**Replicate Values**	INH	AcINH	INA	AcHz	INH	AcINH	INA	AcHz
	406107.1	39293.9	348997.4	7391.15	2708792	219462.9	46135.13	17614.45
	384799.6	34801.53	304601.5	5866.129	3117099	267642.9	48791.23	22250.6
	598522.2	51407.84	415226.4	8357.265	2969332	254540.3	45766.2	21525.77
	696207.3	34525.06	290880.5	8021.404	2555781	208853.8	40823.62	16261.81
	405209.1	33316.39	635452.4	6593.028	2600473	204010.1	40608.86	19118.68
**Mean**	498169.1	38668.95	399031.6	7245.795	2790295	230902	44425.01	19354.26
**Nominal value**	578116	41211	366893	8390	2754766	230292.4	73060.15	18562.03
**Accuracy**	-13.8289	-6.16839	8.759676	-13.6377	1.289734	0.264702	**-39.1939**	4.267986
**Concentration level**	**MQC**				**HQC**			
**Replicate Values**	INH	AcINH	INA	AcHz	INH	AcINH	INA	AcHz
	17124078	8704032	3491431	22772.15	34950139	17549319	7121436	56243.3
	16508781	8110561	2983361	27915.22	29505045	14423242	5654805	46445.62
	16692426	8661707	3309293	28807.74	25283633	15460471	5933983	51220.99
	15185756	7912390	2966133	24993.5	30750816	15242941	6040189	48576.82
	16730017	8360420	3221317	28598.43	30152275	16607950	6480353	59764.73
**Mean**	16448212	8349822	3194307	26617.41	30128382	15856784	6246153	52450.29
**Nominal value**	17880951	9072709	3650869	25473	30268293	15848723	6569018	50834
**Accuracy**	-8.01266	-7.96771	-12.5056	4.49263	-0.46224	0.050864	-4.91497	3.179548

Table S22. Detailed results of accuracy analysis – Intraday 2								
**Concentration level**	**LLOQ**				**LQC**			
**Replicate Values**	INH	AcINH	INA	AcHz	INH	AcINH	INA	AcHz
	467199.8	65824.71	407738.3	7531.007	3572854	298178		24684.9
	468881.9	34230.83	367869.9	7873.37	2816911	238535.2	69615.41	17575.23
	703700.1	57156.24	494479.8	10386.65	2957293	229341.6	67532.49	19520.27
	481162.4	54444.49	310896.6	7752.93	2608131	193549.6	54477.8	16561.65
	446642.8	63033.23	371525	7248.255	2785009	209918.6	57851.45	18315.22
**Mean**	513517.4	54937.9	390501.9	8158.443	2948040	233904.6	62369.29	19331.45
**Nominal value**	578116	41211	366893	8390	2754766	230292.4	73060.15	18562.03
**Accuracy**	-11.174	33.30883	6.434818	-2.75992	7.01596	1.568527	-14.633	4.145124
**Concentration level**	**MQC**				**HQC**			
**Replicate Values**	INH	AcINH	INA	AcHz	INH	AcINH	INA	AcHz
	17424741	8878564	3498148	30439.9	28858183	17167109	6797936	53507.73
	14252757	7688880	3043569	25994.91	25271305	14440650	5522019	44567.79
	17468401	8671074	3391725	30757.02	30934837	15661909	5995675	53130.89
	14947910	7885949	2951177	23671.39	29677759	14795630	5842361	50647.27
	16822125	8330542	3230074	26383.14	32365616	16810878	6516906	57969.88
**Mean**	16183187	8291002	3222939	27449.27	29421540	15775235	6134979	51964.71
**Nominal value**	17880951	9072709	3650869	25473	30268293	15848723	6569018	50834
**Accuracy**	-9.49482	-8.61603	-11.7213	7.758304	-2.79749	-0.46368	-6.60736	2.224322

Table S23. Detailed results of accuracy analysis – Inter Day 1

Concentration level	LLOQ				LQC			
Replicate Values	INH	AcINH	INA	AcHz	INH	AcINH	INA	AcHz
	570349.8	54287.01	481429.2	9526.549	2917502	251537.1	59714.91	18604.86
	557680.1	60900.78	462086.4	7978.213	2666907	239292.1	59965.07	16251.41
	453701.3	25745.28	356812.6	4950.18	2655412	229390.8	66257.53	16255.91
	302625	48155.71	399392.1	8851.948	2919094	241565.3	71588.07	17001.18
	559510.1	53369.82	336255.8	7516.715	2891075	230699.9	62034.19	17385.93
**Mean**	488773.3	48491.72	407195.2	7764.721	2809998	238497	63911.95	17099.86
**Nominal value**	578116	41211	366893	8390	2754766	230292.4	73060.15	18562.03
**Accuracy**	-15.4541	17.66694	10.98473	-7.45267	2.004957	3.562697	-12.5215	-7.87724
**Concentration level**	**MQC**				**HQC**			
**Replicate Values**	INH	AcINH	INA	AcHz	INH	AcINH	INA	AcHz
	20509721	9650244	3453981	31711.08	31982261	16303842	6247330	61845.74
	15730926	6535610	3140590	22275.49	25657683	16086517	4862922	44428.59
	14507386	8045583	2925482	27238.66	30859400	14899282	6120017	52543.52
	16706662	7507235	3344622	26212.64	29222831	16197500	5621800	53970.83
	13911146	8531080	2729515	23227.6	31773090	13330116	6287755	42861.75
**Mean**	16273168	8053950	3118838	26133.09	29899053	15363452	5827965	51130.09
**Nominal value**	17880951	9072709	3650869	25473	30268293	15848723	6569018	50834
**Accuracy**	-8.9916	-11.2288	-14.5727	2.591344	-1.21989	-3.0619	-11.281	0.582458

Table S24. Detailed results of accuracy analysis – Inter Day 2								
**Concentration level**	**LLOQ**				**LQC**			
**Replicate Values**	INH	AcINH	INA	AcHz	INH	AcINH	INA	AcHz
	393142.7	37753.21	276359.5	4822.691	3289477	250627.8	53037.12	19913.23
	437033.9	37910.14	308494.3	4785.057	3346213	257265.7	60777.97	19802.96
	351918.3	33888.66	295844.9	3745.045	3096107	206830.7	53167.9	18792.57
	459107.5	49130.51	246107.6	4303.328	2688256	209161.6	47910.18	16956.11
	352664.8	24733.32	215692.8	3529.343	2777364	230356.1	49360.11	17088.5
**Mean**	398773.5	36683.17	268499.8	4237.093	3039483	230848.4	52850.66	18510.68
**Nominal value**	418338	31431	244998	4478	2754766	230292.4	73060.15	18562.03
**Accuracy**	-4.67673	16.71015	9.59266	-5.37979	10.33544	0.241421	**-27.6615**	-0.27669
**Concentration level**	**MQC**				**HQC**			
**Replicate Values**	INH	AcINH	INA	AcHz	INH	AcINH	INA	AcHz
	13336551	6816157	2403101	23404.33	24322908	11570926	4116068	42367
	12034025	6103595	2175343	21330.58	23765400	11545440	4352128	43035.63
	12237036	7431630	2173529	24301.5	24689540	12282063	4553801	41921.38
	9209309	4539201	1643002	15552.23	26263673	12863412	4563408	42262.31
	12234510	6143399	2241792	20700.37	22713731	11256041	3767625	40500.67
**Mean**	11810286	6206796	2127353	21057.8	24351050	11903576	4270606	42017.4
**Nominal value**	12140873	6269092	2330790	19069	21725605	11244260	4164806	37258
**Accuracy**	-2.72292	-0.9937	-8.72823	10.4295	12.08457	5.863582	2.540333	12.77416

Table S25. Detailed results of accuracy analysis – Inter Day 3								
**Concentration level**	**LLOQ**				**LQC**			
**Replicate Values**	INH	AcINH	INA	AcHz	INH	AcINH	INA	AcHz
	869025.8	63913.37	33277.48	8776.621	1720592	197640.8	990666.3	18543.72
	979921.3	64957.37	41998.65	9315.663	1675428	210015.9	1038628	18709.12
	945572.1	59398.27	13488.73	7319.526	1663584	206638.8	1031591	17104.33
	774343	50270.84	24544.49	9934.372	1687377	216213.7	971231	19677.73
	1112953	54258.72	38521.99	8274.946	1697909	189155.5	1064702	18481.04
**Mean**	936363	58559.71	30366.27	8724.226	1686745	207627.3	1019364	18508.73
**Nominal value**	984781.1	64886.28	30131.3	7322.505	1794983	242314	1188201	16641
**Accuracy**	-4.91663	-9.75024	0.779801	19.14263	-6.03001	-14.3148	-14.2095	11.22364
**Concentration level**	**MQC**				**HQC**			
**Replicate Values**	INH	AcINH	INA	AcHz	INH	AcINH	INA	AcHz
	45645448	19674423	7861191	32396.77	56838215	26419291	10238054	34062.97
	34944042	13573186	5825749	25650.03	50363113	20077466	8670069	27807.89
	35969042	14101632	6230126	24992.4	50599541	20802827	8535585	32275.12
	35209011	13437337	6095462	25399.27	48898659	20668021	8344451	29318.65
	36022068	14760944	5985840	20967.27	38787560	17625594	6751575	23645.2
**Mean**	37557922	15109504	6399674	25881.15	49097418	21118640	8507947	29421.96
**Nominal value**	35484532	13455405	5830339	24429.53	47590030	19287789	8745434	30184.33
**Accuracy**	5.843082	12.2932	9.765047	5.942057	3.167445	9.49228	-2.71556	-2.52569

<u>Results of parameters evaluated during method development of Hydrazine derivatized using p-tolualdehyde (p-THz).</u>

1. **Linearity**

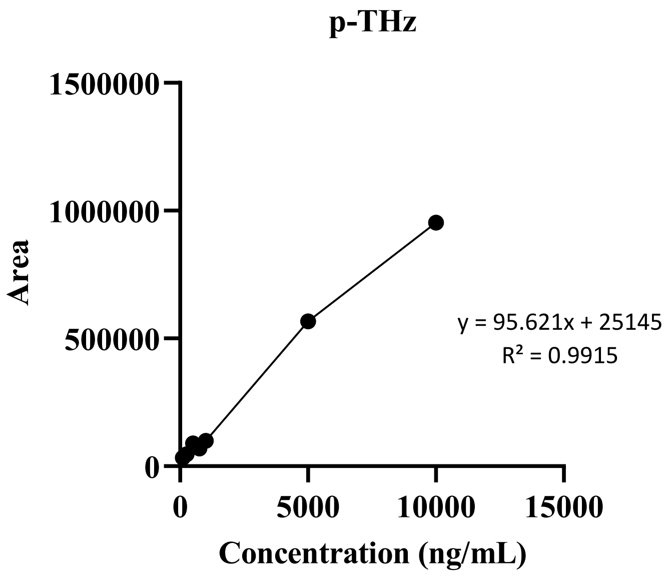

Figure S39. Linearity of trueness of back-calculated result for 1,2-bis((E)-4-methylbenzylidene)hydrazine (Derivatized form of hydrazine using p-tolualdehyde – p-THz)

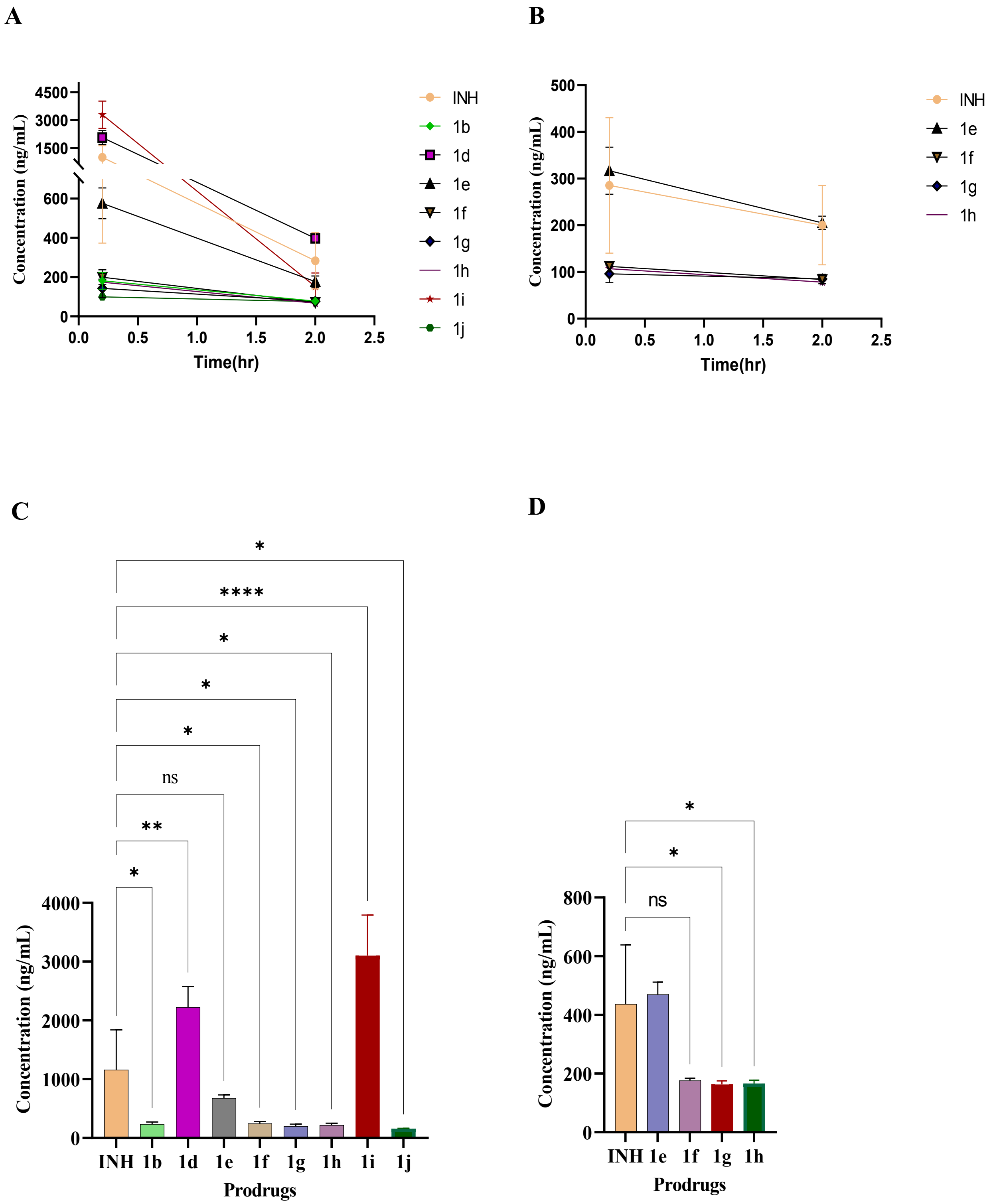

Figure S40. Time versus plasma concentration profile of *in* vivo released INH**(A)** and AcINH**(B)** and eAUC of *in-vivo* released INH**(C)** and AcINH**(D)** post oral administration of INH and prodrugs **1a-1j** in a single oral dose (10mg/kg) administration to Balb/C mice (n=3). Data is expressed as the mean ± SD, with statistics quantified using one-way ANOVA with Dunnett's multiple comparison test. *p≤0.05, **p≤0.01, ***p≤0.001, ****p≤0.001 [a]*In vivo* released INH released from prodrugs **1a, 1c** reported as BLQ. [b]*In vivo* released AcINH from prodrugs **1a,1b,1c,1d**, **1i** and **1j** were reported as BLQ.

Table S26. Body weight and feed intake of animals used in repetitive single oral dosing of INH and prodrug 1d.								
Day	Body Weight(g)						Feed intake(g)	
	INH group (n=3)			1d group (n=3)			Cage 1	Cage 2
	Animal no.1	Animal no.2	Animal no.3	Animal no.1	Animal no.2	Animal no.3	INH group	1d group
0	26	26	27	26	25.5	27	9	11
1	26.5	26.5	27	26	25.5	28	13	11.5
2	27	26.5	27	25.5	24	27	11	10.5
3	27	26	27	26	25	27	9	10
4	26	26.5	27	25.5	25	27	11.5	11
5	26	26	27	25	24.5	27	15.5	14.5
6	26	27	27.5	26.5	25.5	27.5	13.5	12
7	25.5	26	27	25.5	25	28.5	14	12
8	26	26.5	27	26	25.5	27	14	13.5
9	26	26	27	25.5	25.5	27	15	12.5
10	26.5	26	28	25.5	25	27	15	12.5

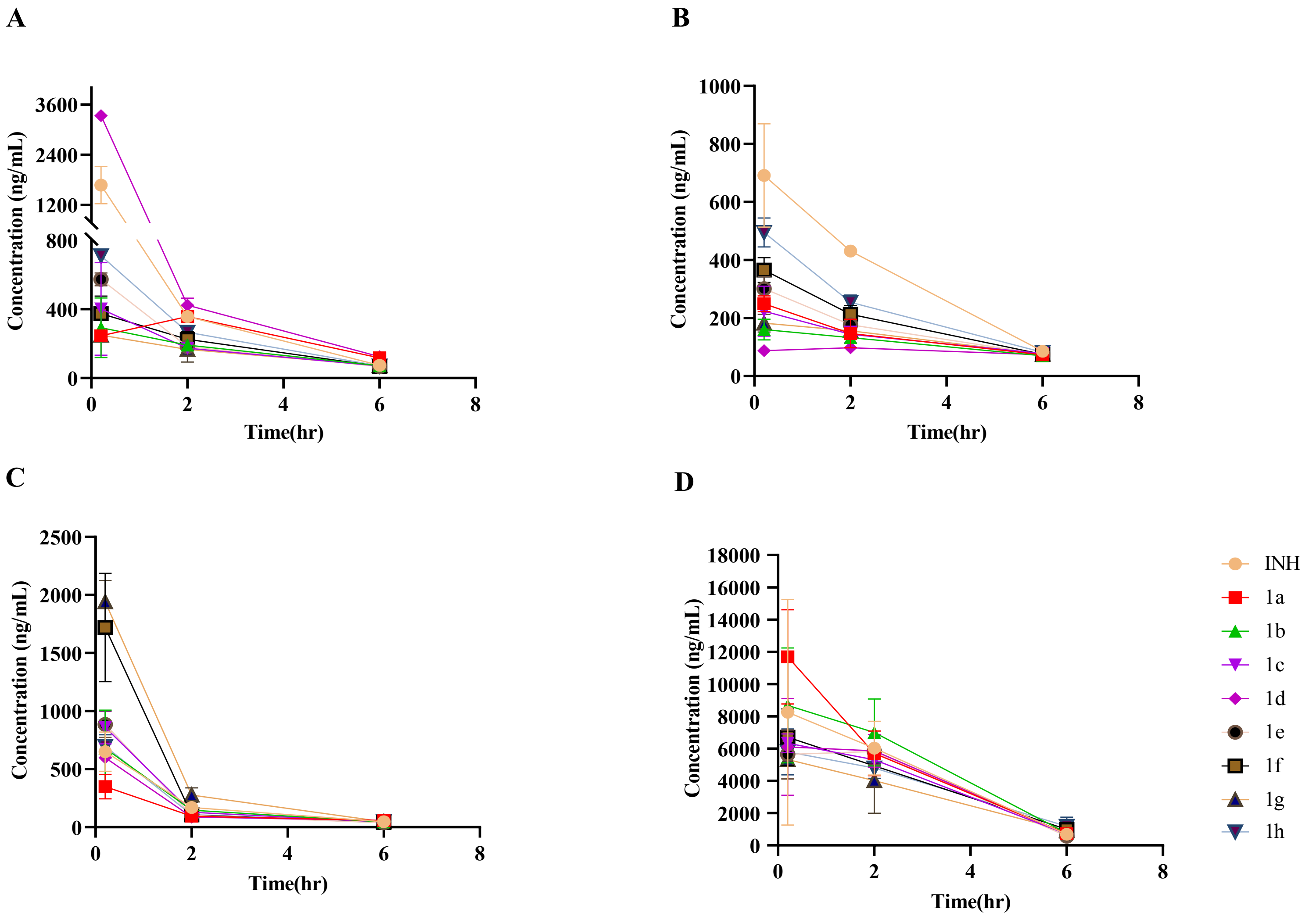

Figure S41. Time versus plasma concentration profile of *in* vivo released INH**(A)**, AcINH**(B)** and INA**(C)** post oral administration of 1st dose of INH at 10mg/kg and prodrugs **1a-1h** at molar equivalent dose of 10mg/kg of INH to Balb/C mice (n=3) via oral gavage in 10-days repetitive single oral dose plasma exposure analysis. Data are expressed as the mean ± SD.

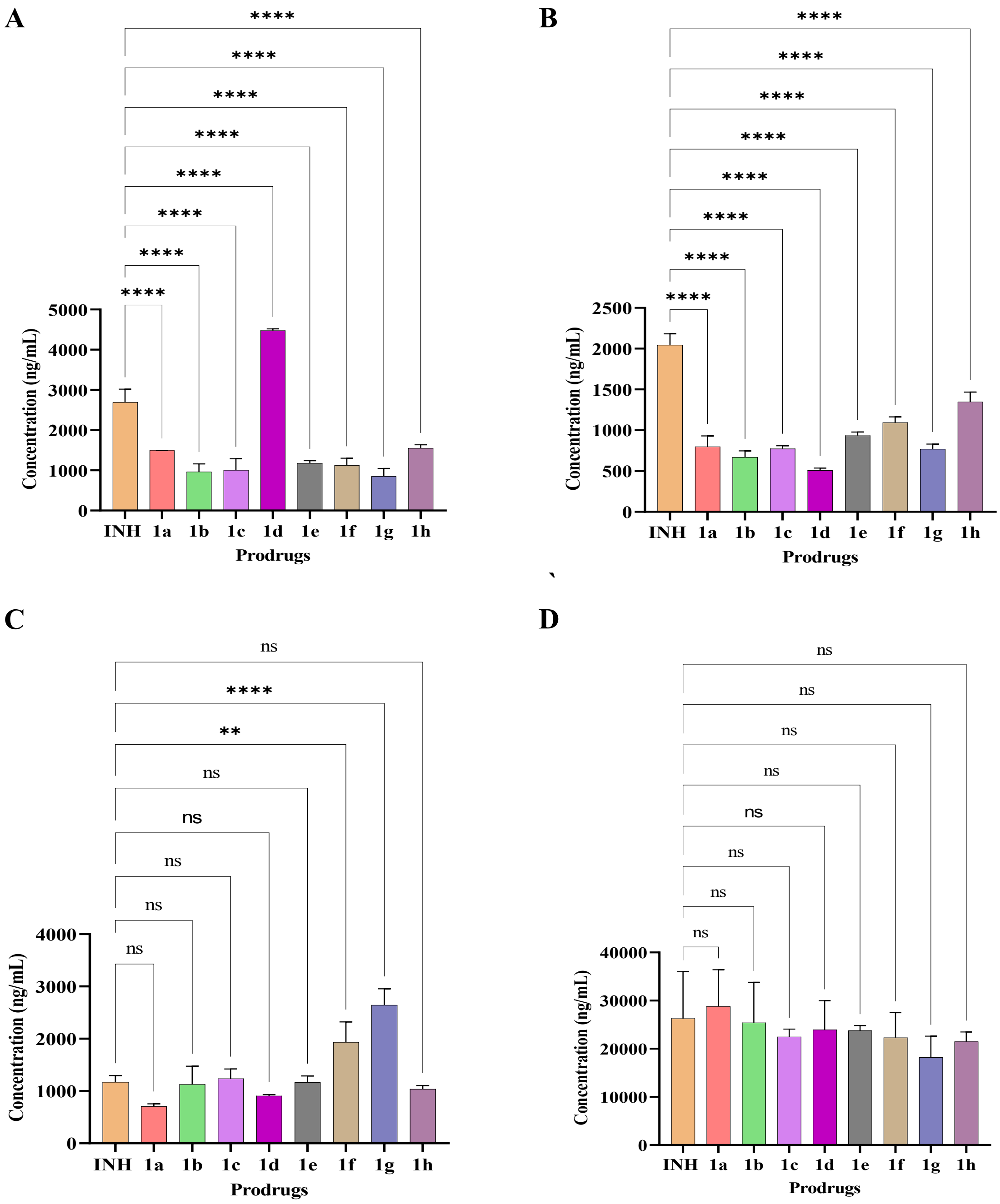

Figure S42. (Graphical representation of data discussed in Table 6A). eAUC (20m-6hr) of *in vivo* released INH**(A)**, AcINH**(B)**, INA**(C) &** AcHz**(D)** post oral administration of 1st dose of INH at 10mg/kg and prodrugs **1a-1h** at molar equivalent dose of 10mg/kg of INH to Balb/C mice (n=3) via oral gavage in 10-days repetitive single oral dose plasma exposure analysis. Data are expressed as the mean ± SD, with statistics quantified using one-way ANOVA with Dunnett's multiple comparison test. Note: *p≤0.05, **p≤0.01, ***p≤0.001, ****p≤0.001, ^{ns}p is non-significant.

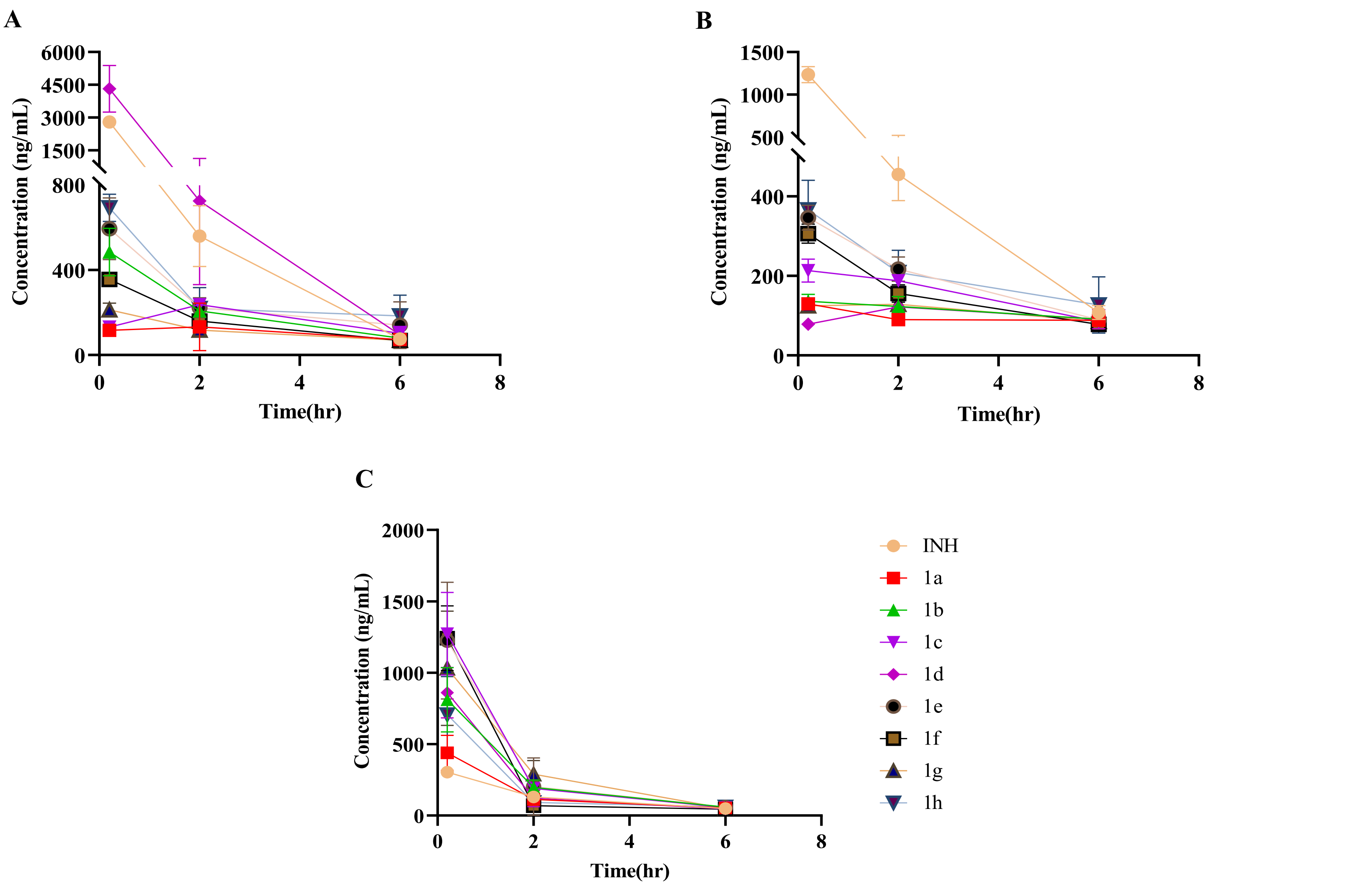

Figure S43. Time versus plasma concentration profile of *in* vivo released INH**(A)**, AcINH**(B)** and INA**(C)** post oral administration of 10th dose of INH at 10mg/kg and prodrugs **1a-1h** at molar equivalent dose of 10mg/kg of INH to Balb/C mice (n=3) via oral gavage in 10-days repetitive single oral dose plasma exposure analysis. Data are expressed as the mean ± SD.

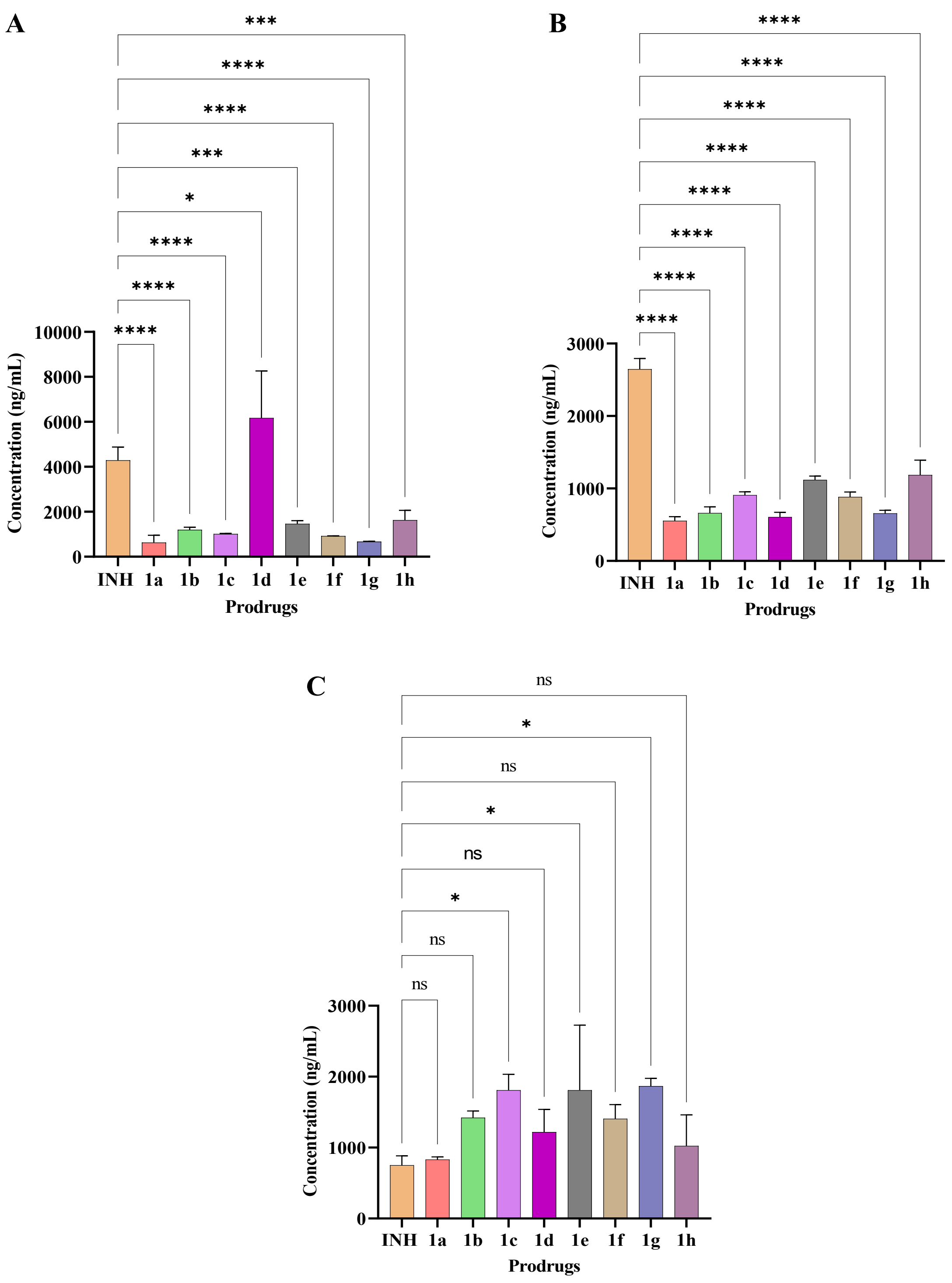

Figure S44. (Graphical representation of data discussed in Table 6B) eAUC (20m-6hr) of *in vivo* released INH**(A)**, AcINH**(B)**, INA**(C) &** AcHz**(D)** post oral administration of 10th dose of INH at 10mg/kg and prodrugs **1a-1h** at molar equivalent dose of 10mg/kg of INH to Balb/C mice (n=3) via oral gavage in 10-days repetitive single oral dose plasma exposure analysis. Data are expressed as the mean ± SD, with statistics quantified using one-way ANOVA with Dunnett's multiple comparison test. Note: *p≤0.05, **p≤0.01, ***p≤0.001, ****p≤0.001, ^{ns}p is non-significant. AcHz levels were BLQ at 10th dose analysis

Table S27. Time versus plasma concentration profile of prodrug 1d and *in vivo* released INH from 1d post oral administration of single dose of prodrug 1d at three incremental doses, 1mg/Kg, 3mg/Kg and 10mg/Kg.

**Dose = 1mg/Kg (n=3)**					**Dose = 3mg/Kg (n=3)**					**Dose = 10mg/Kg (n=3)**				
**1d**					**1d**					**1d**				
Time(hr)	Conc.(ng/ml)			Mean	Time(hr)	Conc.(ng/ml)			Mean	Time(hr)	Conc.(ng/ml)			Mean
	1	2	3			1	2	3			1	2	3	
0.083	2678.90	1450.78	1912.08	2013.92	0.083	4448.87	5417.80	3994.39	4620.35	0.083	14572.33	17224.56	18367.29	16721.39
2	538.88	230.49	226.62	332.00	2	540.20	685.31	509.34	578.28	2	1701.64	1878.09	2496.68	2025.47
6	145.10	144.63	149.43	146.38	6	155.82	142.78	154.05	150.88	6	183.82	173.56	292.98	216.79
**1d-INH**					**1d-INH**					**1d-INH**				
Time(hr)	Conc.(ng/ml)			Mean	Time(hr)	Conc.(ng/ml)			Mean	Time(hr)	Conc.(ng/ml)			Mean
	1	2	3			1	2	3			1	2	3	
0.083	362.82	211.08	273.14	282.35	0.083	563.66	669.70	535.60	589.65	0.083	1736.03	1980.12	2170.69	1962.28
2	106.43	69.98	70.08	82.16	2	108.80	124.46	107.58	113.61	2	237.59	268.31	343.43	283.11
6	56.83	119.46	56.11	77.47	6	57.06	54.58	57.47	56.37	6	67.86	55.50	84.63	69.33

Table S28. Time versus plasma concentration profile of naïve INH post oral administration of single dose of naïve INH at three incremental doses, 1mg/Kg, 3mg/Kg and 10mg/Kg.

**Dose = 1mg/Kg (n=3)**					**Dose = 3mg/Kg (n=3)**					**Dose = 10mg/Kg (n=3)**				
**INH**					**INH**					**INH**				
Time(hr)	Conc.(ng/ml)			Mean	Time(hr)	Conc.(ng/ml)			Mean	Time(hr)	Conc.(ng/ml)			Mean
	1	2	3			1	2	3			1	2	3	
0.083	129.32	149.40	108.32	129.01	0.083	455.36	374.24	353.31	394.30	0.083	737.12	378.52	808.31	641.32
2	BLQ	BLQ	BLQ	BLQ	2	105.66	77.94	78.02	87.20	2	153.74	155.29	165.65	158.23
6	BLQ	BLQ	BLQ	BLQ	6	55.76	54.35	60.62	56.91	6	68.66	65.53	62.23	65.47

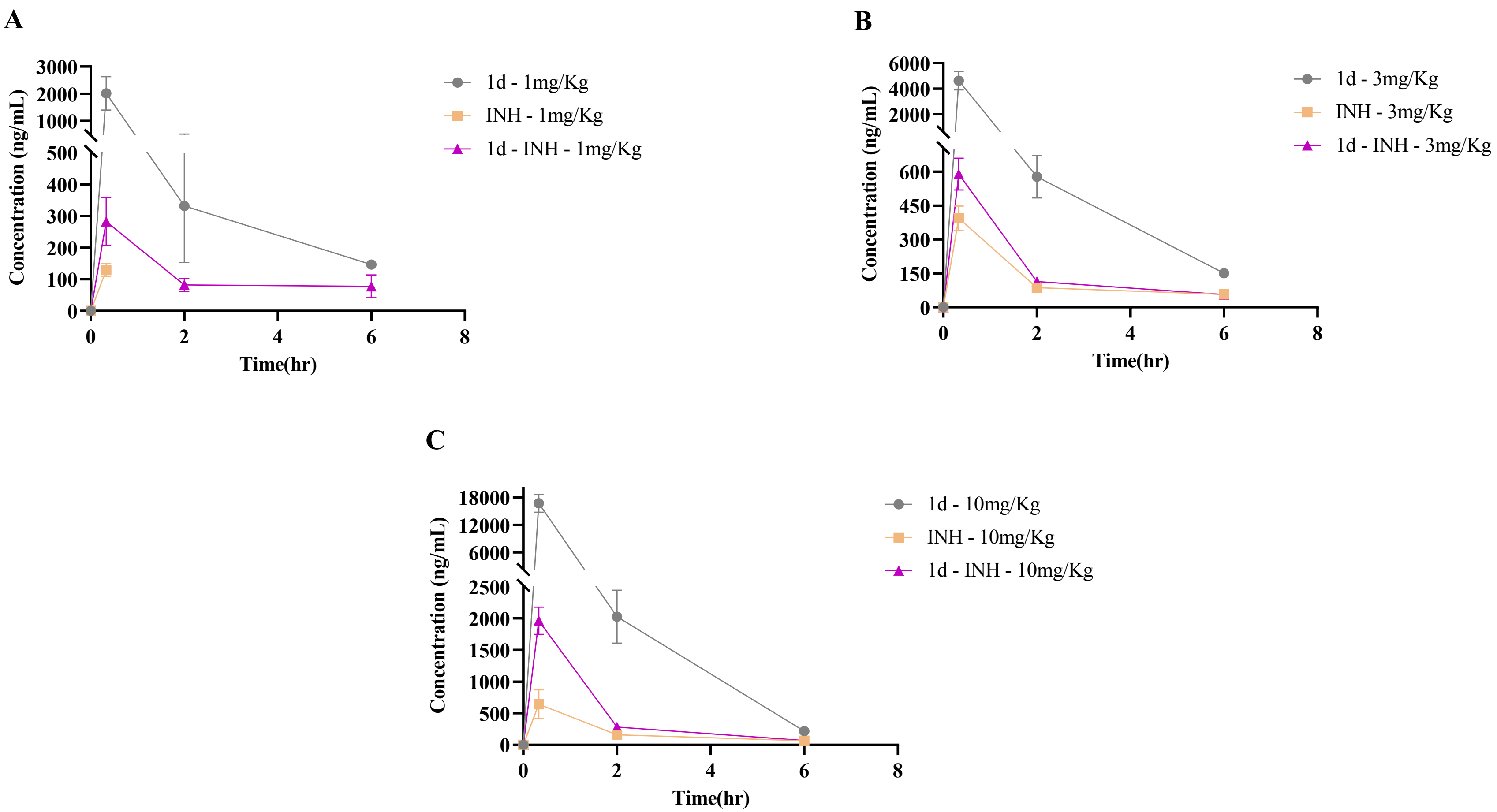

Figure S45. Time versus plasma concentration profile of unchanged **1d** and free INH after oral administration of single dose of INH and **1d** at three incremental doses, 1mg/Kg **(A)**, 3mg/Kg**(B)**, 10mg/Kg**(C)** to Balb/C mice (n=3) via oral gavage. Data are expressed as the mean ± SD.

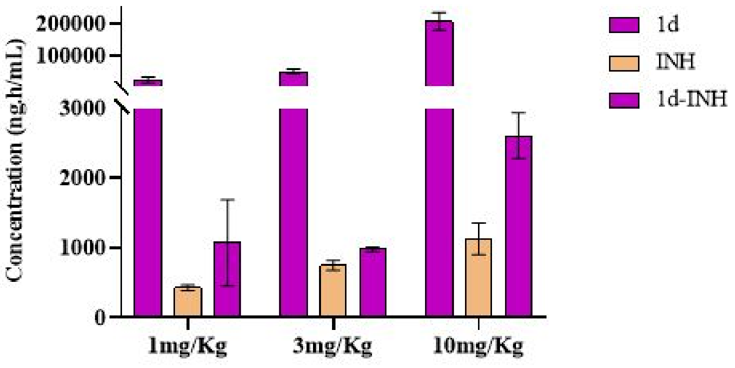

Figure S46. eAUC (20m-6hr) of prodrug **1d** and free INH min after oral administration of single dose of INH and **1d** at three incremental doses (1 mg/kg, 3 mg/kg and 10 mg/kg) to Balb/C mice (n=3) via oral gavage. Data are expressed as the mean ± SD, with statistics quantified using one-way ANOVA with Dunnett's multiple comparison test.

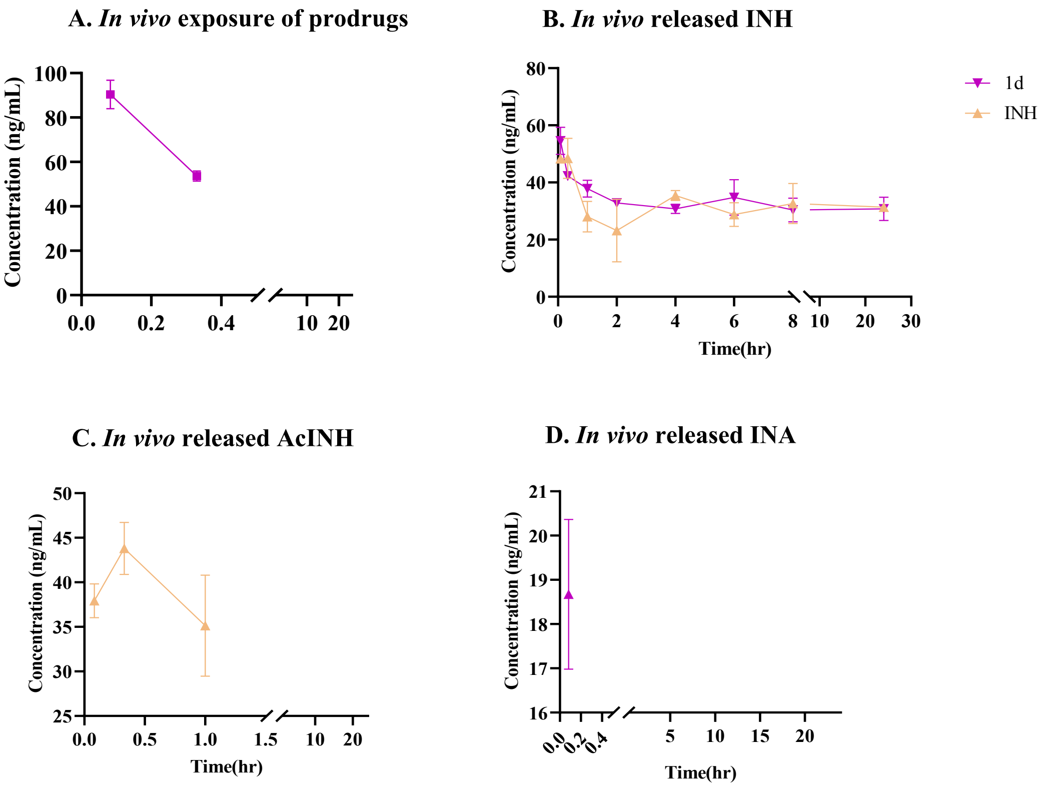

Figure S47. Pharmacokinetic profile of prodrugs **1d** (A), *in vivo* released INH (B), AcINH (C) and INA (D) after intravenous administration of **INH** at 1mg/kg and prodrug **1d** at molar equivalent dose of 1mg/kg of INH to Balb/C mice (n=12) via oral gavage. Data are expressed as the mean ± SD.

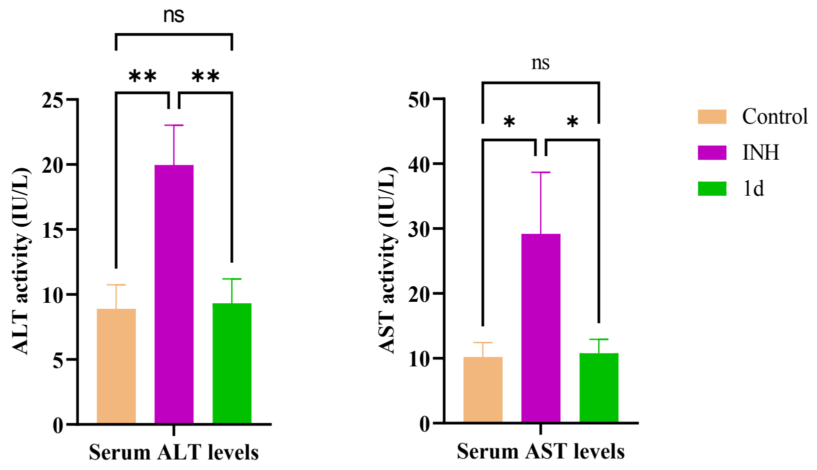

Figure S48. Serum ALT and AST levels after repeat single oral dose daily for 6 consecutive days (blood was drawn post 24hours of 6th dose) in Balb/C mice (n=3/group). Data are expressed as the mean ± SD.

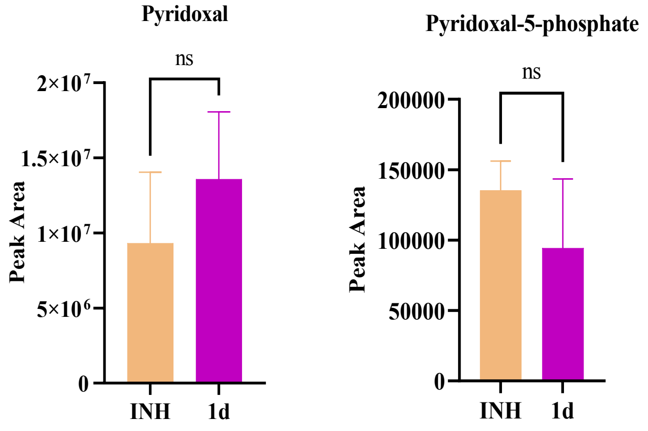

Figure S49. Tissue levels of pyridoxal and pyridoxal-5-phosphate in mice liver using LC-MS analysis. Data are expressed as the mean ± SD.

Table S29. Time versus plasma concentration profile of unchanged 1a, 1d and 1h post oral administration of single dose (n=12) of prodrug 1a, 1d and 1h.

1a					1d				
Time(hr)	Conc.(ng/ml)			Mean	Time(hr)	Conc.(ng/ml)			Mean
	1	2	3			1	2	3	
0.083	204865.9	227622.6	232634	221707.50	0.083	11961.49	18487.69	25064.14	18504.44
0.33	140123.6	97160.41	206550.6	147944.87	0.33	16075.57	20423.13	19573.42	18690.71
1	60672.01	60892.86	67980.6	63181.82	1	11417.74	10973.11	8868.864	10419.91
2	39920.11	31330.51	23089.62	31446.75	2	3743.369	4565.668	5554.661	4621.23
4	866.9608	2690.868	637.768	1398.53	4	1132.01	804.2136	888.7332	941.65
6	1211.012	312.3524	189.113	570.83	6	439.0105	224.9614	378.3156	347.43
24	165.8902	230.7549	227.6799	208.11	24	154.4649	152.0391	150.3251	152.28

1h				
Time(hr)	Conc.(ng/ml)			Mean
	1	2	3	
0.083	8309.855	8394.421	6752.782	7819.02
0.33	8404.671	5357.2	4622.26	6128.04
1	565.4766	484.0636	250.067	433.20
2	310.6722	690.4203	227.243	409.45
4	2412.197	129.4626	109.204	883.62
6	118.4985	139.1431	126.6184	128.09
24	106.1236	145.8704	107.9619	119.99

Table S30. Time versus plasma concentration profile of *in* vivo free INH post oral administration of single dose (n=12) of naïve INH, prodrug 1a, 1d and 1h.

INH					1a-INH				
Time(hr)	Conc.(ng/ml)			Mean	Time(hr)	Conc.(ng/ml)			Mean
	1	2	3			1	2	3	
0.083	2842.817	2176.361	2658.456	2559.21	0.083	96.98985	96.68781	111.1542	101.61
0.33	2276.726	1716.638	1528.251	1840.54	0.33	72.51123	66.57322	103.1242	80.74
1	918.1586	866.3449	1042.327	942.28	1	BLQ	BLQ	BLQ	BLQ
2	467.2928	409.4642	496.7089	457.82	2	BLQ	BLQ	BLQ	BLQ
4	104.5813	89.98132	54.35761	82.97	4	BLQ	BLQ	BLQ	BLQ
6	BLQ	BLQ	BLQ	BLQ	6	BLQ	BLQ	BLQ	BLQ
24	BLQ	BLQ	BLQ	BLQ	24	BLQ	BLQ	BLQ	BLQ
**1d-INH**					**1h-INH**				
Time(hr)	Conc.(ng/ml)			Mean	Time(hr)	Conc.(ng/ml)			Mean
	1	2	3			1	2	3	
0.083	695.487	2791.179	3919.954	2468.87	0.083	394.4848	348.7003	545.8466	429.68
0.33	1947.61	3011.908	2924.214	2627.91	0.33	622.3192	408.2692	329.7863	453.46
1	1618.725	1626.62	1391.053	1545.47	1	229.4225	159.3997	242.0781	210.30
2	540.1717	654.2349	801.6281	665.34	2	145.8009	107.9857	99.53856	117.78
4	171.1293	137.318	140.7702	149.74	4	BLQ	BLQ	BLQ	BLQ
6	BLQ	BLQ	BLQ	BLQ	6	BLQ	BLQ	BLQ	BLQ
24	BLQ	BLQ	BLQ	BLQ	24	BLQ	BLQ	BLQ	BLQ

Table S31. Time versus plasma concentration profile of *in* vivo released AcINH post oral administration of a single dose (n=12) of naïve INH, prodrug 1a, 1d and 1h.

INH-AcINH					1a-AcINH				
Time(hr)	Conc.(ng/ml)			Mean	Time(hr)	Conc.(ng/ml)			Mean
	1	2	3			1	2	3	
0.083	818.9166	692.1579	893.4634	801.51	0.083	109.1195	105.4118	106.9276	107.15
0.33	1007.012	846.2219	708.4128	853.88	0.33	90.59214	91.07944	115.5583	99.08
1	608.7053	545.6514	629.4317	594.60	1	BLQ	BLQ	BLQ	BLQ
2	421.6951	364.3592	410.1646	398.74	2	BLQ	BLQ	BLQ	BLQ
4	135.069	132.8243	69.97767	112.62	4	BLQ	BLQ	BLQ	BLQ
6	BLQ	BLQ	BLQ	BLQ	6	BLQ	BLQ	BLQ	BLQ
24	BLQ	BLQ	BLQ	BLQ	24	BLQ	BLQ	BLQ	BLQ
**1d-AcINH**					**1h-AcINH**				
Time(hr)	Conc.(ng/ml)			Mean	Time(hr)	Conc.(ng/ml)			Mean
	1	2	3			1	2	3	
0.083	BLQ	BLQ	BLQ	BLQ	0.083	220.947	203.4952	272.7854	232.41
0.33	BLQ	BLQ	BLQ	BLQ	0.33	334.5211	269.5359	220.6781	274.91
1	BLQ	BLQ	BLQ	BLQ	1	185.9086	141.7181	208.734	178.79
2	BLQ	BLQ	BLQ	BLQ	2	149.1862	126.1558	116.3771	130.57
4	BLQ	BLQ	BLQ	BLQ	4	BLQ	BLQ	BLQ	BLQ
6	BLQ	BLQ	BLQ	BLQ	6	BLQ	BLQ	BLQ	BLQ
24	BLQ	BLQ	BLQ	BLQ	24	BLQ	BLQ	BLQ	BLQ

Table S32. Time versus plasma concentration profile of *in* vivo released INA post oral administration of a single dose (n=12) of naïve INH, prodrug 1a, 1d and 1h.

INH-INA					1a-INA				
Time(hr)	Conc.(ng/ml)			Mean	Time(hr)	Conc.(ng/ml)			Mean
	1	2	3			1	2	3	
0.083	209.0293	180.358	210.8863	200.09	0.083	265.1322	304.0864	354.3666	307.86
0.33	199.8042	158.3675	131.6976	163.29	0.33	182.1337	216.3549	339.3433	245.94
1	108.2093	98.18351	112.6267	106.34	1	90.19316	87.84328	125.93	101.32
2	82.07842	72.40684	77.50687	77.33	2	68.27472	79.24001	71.34977	72.95
4	BLQ	BLQ	BLQ	BLQ	4	BLQ	BLQ	BLQ	BLQ
6	BLQ	BLQ	BLQ	BLQ	6	BLQ	BLQ	BLQ	BLQ
24	BLQ	BLQ	BLQ	BLQ	24	BLQ	BLQ	BLQ	BLQ
**1d-INA**					**1h-INA**				
Time(hr)	Conc.(ng/ml)			Mean	Time(hr)	Conc.(ng/ml)			Mean
	1	2	3			1	2	3	
0.083	395.29	542.6778	780.0805	572.68	0.083	709.8128	445.8232	822.3592	659.33
0.33	447.2756	632.0048	574.5884	551.29	0.33	496.0741	220.1536	172.4939	296.24
1	245.1865	271.4983	223.2028	246.63	1	59.30926	49.47526	56.43108	55.07
2	96.19191	127.5081	134.5893	119.43	2	49.94755	68.97178	38.15328	52.36
4	BLQ	BLQ	BLQ	BLQ	4	BLQ	BLQ	BLQ	BLQ
6	BLQ	BLQ	BLQ	BLQ	6	BLQ	BLQ	BLQ	BLQ
24	BLQ	BLQ	BLQ	BLQ	24	BLQ	BLQ	BLQ	BLQ

Table S33. Time versus plasma concentration profile of *in* vivo released AcHz post oral administration of a single dose (n=12) of naïve INH, prodrug 1a, 1d and 1h.

**INH-AcHz**					**1a- AcHz**				
Time(hr)	Conc.(ng/ml)			Mean	Time(hr)	Conc.(ng/ml)			Mean
	1	2	3			1	2	3	
0.083	BLQ	BLQ	BLQ	BLQ	0.083	BLQ	BLQ	BLQ	BLQ
0.33	BLQ	BLQ	BLQ	BLQ	0.33	BLQ	BLQ	BLQ	BLQ
1	BLQ	BLQ	BLQ	BLQ	1	BLQ	BLQ	BLQ	BLQ
2	BLQ	BLQ	BLQ	BLQ	2	BLQ	BLQ	BLQ	BLQ
4	BLQ	BLQ	BLQ	BLQ	4	BLQ	BLQ	BLQ	BLQ
6	BLQ	BLQ	BLQ	BLQ	6	BLQ	BLQ	BLQ	BLQ
24	BLQ	BLQ	BLQ	BLQ	24	BLQ	BLQ	BLQ	BLQ
**1d- AcHz**					**1h- AcHz**				
Time(hr)	Conc.(ng/ml)			Mean	Time(hr)	Conc.(ng/ml)			Mean
	1	2	3			1	2	3	
0.083	BLQ	BLQ	BLQ	BLQ	0.083	BLQ	BLQ	BLQ	BLQ
0.33	BLQ	BLQ	BLQ	BLQ	0.33	BLQ	BLQ	BLQ	BLQ
1	BLQ	BLQ	BLQ	BLQ	1	BLQ	BLQ	BLQ	BLQ
2	BLQ	BLQ	BLQ	BLQ	2	BLQ	BLQ	BLQ	BLQ
4	BLQ	BLQ	BLQ	BLQ	4	BLQ	BLQ	BLQ	BLQ
6	BLQ	BLQ	BLQ	BLQ	6	BLQ	BLQ	BLQ	BLQ
24	BLQ	BLQ	BLQ	BLQ	24	BLQ	BLQ	BLQ	BLQ

Table S34. Time versus plasma concentration profile of *in* vivo released Hz post oral administration of a single dose (n=12) of naïve INH and prodrug 1d.

INH-Hz					1d- Hz				
Time(hr)	Conc.(ng/ml)			Mean	Time(hr)	Conc.(ng/ml)			Mean
	1	2	3			1	2	3	
0.083	BLQ	BLQ	BLQ	BLQ	0.083	BLQ	BLQ	BLQ	BLQ
0.33	BLQ	BLQ	BLQ	BLQ	0.33	BLQ	BLQ	BLQ	BLQ
1	BLQ	BLQ	BLQ	BLQ	1	BLQ	BLQ	BLQ	BLQ
2	BLQ	BLQ	BLQ	BLQ	2	BLQ	BLQ	BLQ	BLQ
4	BLQ	BLQ	BLQ	BLQ	4	BLQ	BLQ	BLQ	BLQ
6	BLQ	BLQ	BLQ	BLQ	6	BLQ	BLQ	BLQ	BLQ
24	BLQ	BLQ	BLQ	BLQ	24	BLQ	BLQ	BLQ	BLQ

**Table S35. Time versus plasma concentration profile of unchanged 1d post intravenous administration of single dose (n=12) of prodrug 1d.**				
**1d**				
Time(hr)	Conc.(ng/ml)			Mean
	1	2	3	
0.083	97.55454	85.11418	88.47365	90.38
0.33	52.31519	52.2384	56.26303	53.61
1	BLQ	BLQ	BLQ	BLQ
2	BLQ	BLQ	BLQ	BLQ
4	BLQ	BLQ	BLQ	BLQ
6	BLQ	BLQ	BLQ	BLQ
8	BLQ	BLQ	BLQ	BLQ
24	BLQ	BLQ	BLQ	BLQ

**Table S36. Time versus plasma concentration profile of *in* vivo released INH post intravenous administration of a single dose (n=12) of naïve INH and prodrug 1d.**									
**INH**					**1d-INH**				
Time(hr)	Conc.(ng/ml)			Mean	Time(hr)	Conc.(ng/ml)			Mean
	1	2	3			1	2	3	
0.083	49.06352	48.12599	47.63943	48.28	0.083	58.1841	49.1854	56.35382	54.57
0.33	41.80972	47.72658	55.83524	48.46	0.33	41.44315	42.59885	42.93385	42.33
1	32.34414	29.69259	22.088	28.04	1	39.95916	35.75093	*1270.534**	37.86
2	35.79595	16.5069	17.19607	23.17	2	31.39614	32.83136	34.22216	32.82
4	37.23224	35.2465	33.76424	35.41	4	29.01959	30.98255	32.24747	30.75
6	24.17977	29.7174	32.35144	28.75	6	28.55747	34.71131	40.99575	34.75
8	40.58015	27.38834	29.92598	32.63	8	25.73637	31.73298	33.68841	30.39
24	31.71884	30.97255	31.44734	31.38	24	31.09881	34.74003	26.55577	30.80

Table S37. Time versus plasma concentration profile of *in* vivo released AcINH post intravenous administration of a single dose (n=12) of naïve INH and prodrug 1d.

INH-AcINH					1d-AcINH				
Time(hr)	Conc.(ng/ml)			Mean	Time(hr)	Conc.(ng/ml)			Mean
	1	2	3			1	2	3	
0.083	36.3296	40.02652	37.44615	37.93	0.083	BLQ	BLQ	BLQ	BLQ
0.33	41.36885	47.04623	42.97863	43.80	0.33	BLQ	BLQ	BLQ	BLQ
1	40.74945	35.24253	29.40199	35.13	1	BLQ	BLQ	BLQ	BLQ
2	BLQ	BLQ	BLQ	BLQ	2	BLQ	BLQ	BLQ	BLQ
4	BLQ	BLQ	BLQ	BLQ	4	BLQ	BLQ	BLQ	BLQ
6	BLQ	BLQ	BLQ	BLQ	6	BLQ	BLQ	BLQ	BLQ
8	BLQ	BLQ	BLQ	BLQ	8	BLQ	BLQ	BLQ	BLQ
24	BLQ	BLQ	BLQ	BLQ	24	BLQ	BLQ	BLQ	BLQ

Table S38. Time versus plasma concentration profile of *in* vivo released INA post intravenous administration of a single dose (n=12) of naïve INH and prodrug 1d.

INH-INA					1d-INA				
Time(hr)	Conc.(ng/ml)			Mean	Time(hr)	Conc.(ng/ml)			Mean
	1	2	3			1	2	3	
0.083	BLQ	BLQ	BLQ	BLQ	0.083	18.66738	16.98271	20.36865	18.67292
0.33	BLQ	BLQ	BLQ	BLQ	0.33	BLQ	BLQ	BLQ	BLQ
1	BLQ	BLQ	BLQ	BLQ	1	BLQ	BLQ	BLQ	BLQ
2	BLQ	BLQ	BLQ	BLQ	2	BLQ	BLQ	BLQ	BLQ
4	BLQ	BLQ	BLQ	BLQ	4	BLQ	BLQ	BLQ	BLQ
6	BLQ	BLQ	BLQ	BLQ	6	BLQ	BLQ	BLQ	BLQ
8	BLQ	BLQ	BLQ	BLQ	8	BLQ	BLQ	BLQ	BLQ
24	BLQ	BLQ	BLQ	BLQ	24	BLQ	BLQ	BLQ	BLQ

Table S39. Time versus plasma concentration profile of *in* vivo released Hz post oral administration of repetitive single dose of naïve INH and prodrug 1d to Balb/C mice (n=3) for six consecutive days.

INH-Hz					1d-Hz				
Time of bleeding	Conc.(ng/ml)			Mean	Time(hr)	Conc.(ng/ml)			Mean
	1	2	3			1	2	3	
Post 24hrs of 6th dose	BLQ	BLQ	BLQ	BLQ	Post 24hrs of 6th dose	BLQ	BLQ	BLQ	BLQ

1. OECD., *OECD Guidelines for the Testing of Chemicals*. 1994: Oecd.
2. Zhou, L., et al., *Development of a high throughput equilibrium solubility assay using miniaturized shake-flask method in early drug discovery.* Journal of pharmaceutical sciences, 2007. **96**(11): p. 3052-3071.
3. Moussa, L.A.t., et al., *Therapeutic isoniazid monitoring using a simple high-performance liquid chromatographic method with ultraviolet detection.* Journal of Chromatography B, 2002. **766**(1): p. 181-187.
4. McAnally, D., et al., *In vivo rapid assessment of compound exposure (RACE) for profiling the pharmacokinetics of novel chemical probes.* Current protocols in chemical biology, 2012. **4**(4): p. 299-309.

5. Fang, P.-F., et al., *Simultaneous determination of isoniazid, rifampicin, levofloxacin in mouse tissues and plasma by high performance liquid chromatography–tandem mass spectrometry.* Journal of Chromatography B, 2010. **878**(24): p. 2286-2291.
6. Li, F., et al., *A high dose of isoniazid disturbs endobiotic homeostasis in mouse liver.* Drug Metabolism and Disposition, 2016. **44**(11): p. 1742-1751.
7. Shen, X., et al., *TidyMass an object-oriented reproducible analysis framework for LC–MS data.* Nature Communications, 2022. **13**(1): p. 4365.
