# Supplementary material for: Carbamate Prodrugs Restrict In Vivo Metabolism and Improve the Pharmacokinetics of Isoniazid
Source: ACS Cent Sci. 2025 Jul 9;11(8):1467–80. doi: 10.1021/acscentsci.5c00576 (PMC12395308; doi:10.1021/acscentsci.5c00576)
Supplement: Supplementary file 2 [file oc5c00576_si_002.pdf]

oc-2025-005769.R1

Name: Peer Review Information for "Labile masking of the free -NH2 group of isoniazid restricts in vivo metabolism and improves pharmacokinetic profile in mice; Design, synthesis and evaluation of carbamate-based prodrugs"

First Round of Reviewer Comments

Reviewer: 1

Comments to the Author

The manuscript presents a well-structured and compelling approach to improve the pharmacokinetic and metabolic profile of isoniazid via carbamate-based prodrug derivatization. The comprehensive chemical, pharmacokinetic, and bioanalytical evaluations are commendable. However, the manuscript would benefit significantly from further toxicological and translational assessments to strengthen the central claim of reduced toxic metabolite formation. The following specific experiments are recommended to address this gap:

Quantification of hydrazine (Hz): Include LC-MS/MS detection of hydrazine, a key hepatotoxic metabolite of INH, in both ex vivo and in vivo samples.

Human liver metabolism assays: Evaluate the metabolic stability and conversion of selected prodrugs using human liver microsomes or primary hepatocytes to assess human relevance.

Toxicity assessment in vivo: Conduct basic liver toxicity assays (e.g., serum ALT/AST levels, liver histopathology) in mice following repeat dosing of INH and lead prodrug(s) to confirm reduced hepatotoxicity.

Assessment of pyridoxine (vitamin B6) depletion: Since INH is known to deplete B6 and cause peripheral neuropathy, evaluating plasma B6 levels post-dosing may help confirm reduced off-target reactivity.

Tissue distribution analysis of intact prodrug: Determine whether significant levels of unmetabolized prodrug (e.g., 1d) accumulate in tissues, which may pose long-term safety concerns.

Reviewer: 2

Comments to the Author

The manuscript presents a novel approach to mitigate the adverse effects of isoniazid by designing carbamate-based prodrugs. This innovative strategy addresses a significant challenge in tuberculosis treatment. The study is methodologically sound and provides comprehensive data on the synthesis, stability, bioconversion, and pharmacokinetics of the prodrugs. The findings suggest that the lead compound (1d) offers improved systemic exposure and reduced formation of undesirable metabolites, potentially enhancing patient compliance and safety.

However, further studies in additional animal models are needed to confirm these results and fully demonstrate the potential of this strategy.

Overall, I have only a few minor remarks:

1) The study focuses on bioconversion by plasma enzymes. Why were hepatic enzymes not considered? For example, see lines 44–49 in the Introduction.

2) Instead of logP, the authors should use logD, as the compounds likely exist in both ionized and unionized forms. Please revise the discussion accordingly (the experimental values are logD, while the predicted values are likely logP).

3) In the Chemistry section, please specify the method used to predict lipophilicity (ie. ChemDraw).

4) The title “Intestinal and stomach...” on line 15 appears to be poorly aligned in the text.

5) The meaning of the values under eAUCs in Table 10 is unclear. Please clarify.

Reviewer: 3

Comments to the Author

This manuscript presents a well-conceived and executed study on the design, synthesis, and pharmacokinetic evaluation of carbamate-based prodrugs of isoniazid (INH), aimed at mitigating its metabolism-mediated toxicity. The rationale—masking the terminal $–NH_2$ group to reduce the formation of hepatotoxic metabolites—is both scientifically sound and of high translational relevance, particularly in the context of improving tuberculosis treatment outcomes. The authors demonstrated that selected prodrugs, especially compound 1d, achieve superior systemic exposure to free INH with concomitant reduction in AcINH levels, one of the key metabolites. The development and application of a derivatization-free LC-MS method for simultaneous quantification of INH and its metabolites adds significant technical value to the study. The pharmacokinetic data, from both single and repeat-dose experiments, convincingly support the potential of this prodrug strategy to enhance INH's therapeutic index.

However, one aspect that could further strengthen this work is the inclusion of preliminary efficacy and safety data—particularly antibacterial activity and hepatotoxicity assessments. Although the pharmacokinetic improvements are promising, confirming that these translate into therapeutic benefits without compromising efficacy or introducing new toxicity would be crucial for advancing toward clinical relevance.

Overall, this is a high-quality contribution that addresses a longstanding challenge in TB chemotherapy. I recommend publication after consideration of the above points.

Author's Response to Peer Review Comments:

Dear Editor,

Thank you very much for the opportunity to revise our manuscript. We sincerely appreciate the reviewers' thoughtful and constructive feedback. In response, we have conducted additional experiments and made revisions to address the comments in detail.

Please find below our point-by-point responses for your kind consideration. As suggested, we are submitting a marked version of the revised manuscript (with new changes highlighted in green for review purposes), along with a clean copy and the updated Supporting Information.

Kindly note that we have added a new co-author, Dr. Aditya Sharma, who contributed significantly to the newly performed repeat-dose toxicity studies for liver injury markers. His name has been included in the revised author list.

We truly hope that the revisions meet the expectations and are acceptable for the next stage of the review process.

On behalf of all co-authors, I would like to express our sincere gratitude for your time, support, and consideration.

Best Regards,

Dinesh

A point-by-point response from the authors

Formatting Needs:

Author List: Please include the email address(es) of the corresponding author(s) on the first page of the manuscript.

Author's Response: The necessary change has been made as suggested on the first page of the revised manuscript.

Supporting Information: Please number all pages in the following format: S1, S2, S3, etc.
Author's Response: The necessary change has been made as suggested.

Synopsis: ACS Central Science requires a brief synopsis. The synopsis should be no more than 200 characters (including spaces) and should reasonably correlate with the Table of Contents (TOC) graphic. The synopsis is intended to explain the importance of the article to a broader readership across the sciences. Please place your synopsis in the manuscript file after the TOC graphic and label as "Synopsis."

Author’s Response: The necessary change has been made as suggested. Please see the last page of the revised manuscript file.

TOC Graphic: Include a TOC graphic illustrating the significance of the paper. The TOC graphic should be something that is representative of your entire work. Color schemes or illustrations typically make good choices. The TOC graphic must be original and free from any copyright issues. Confirm that all text is legible. Present the TOC graphic on the last page of the manuscript by itself. Please label the TOC as “TOC Graphic”. A caption describing the TOC is not needed. Please see more information/guidelines for TOC Graphics at the following link:
http://pubsapp.acs.org/paragonplus/submission/toc_abstract_graphics_guidelines.pdf?

Author’s Response: The necessary change has been made as suggested. Please see the last page of the revised manuscript file.

Reviewer(s)' Comments to Author: Reviewer:
1

Recommendation: Major revisions required.

Comments:
The manuscript presents a well-structured and compelling approach to improve the pharmacokinetic and metabolic profile of isoniazid via carbamate-based prodrug derivatization. The comprehensive chemical, pharmacokinetic, and bioanalytical evaluations are commendable. However, the manuscript would benefit significantly from further toxicological and translational assessments to strengthen the central claim of reduced toxic metabolite formation. The following specific experiments are recommended to address this gap:
Author’s Response: The authors express their gratitude for the appreciation of their work by the reviewer.

Quantification of hydrazine (Hz): Include LC-MS/MS detection of hydrazine, a key hepatotoxic metabolite of INH, in both ex vivo and in vivo samples.

Author's response: We thank the reviewer for their insightful suggestion. The novel derivatization-free method developed and presented in this manuscript is not suitable for the quantification of hydrazine (Hz) due to its low molecular weight.

In response to the reviewer's comment, we established a known LC-MS method in our laboratory that involves a derivatization step, as reported in the literature (Molecules, 2022, p. 8607; Journal of Chromatography B, 2017, p. 189). Using this method, we attempted absolute quantification of Hz in leftover plasma samples from a 10 mg/kg single oral dose pharmacokinetic (PK) study of INH and lead compound 1d. In these samples, free hydrazine levels were found to be below the limit of detection.

We further applied the same method to quantify Hz in plasma samples from a newly conducted 90 mg/kg repeat-dose study (originally performed for ALT/AST analysis; see below). Again, hydrazine levels in both study groups were below the limit of quantification (BLQ).

These findings have been incorporated into the Results section of the main manuscript, and the detailed analytical methodology is provided in the Supporting Information file.

Human liver metabolism assays: Evaluate the metabolic stability and conversion of selected prodrugs using human liver microsomes or primary hepatocytes to assess human relevance.

Author's Response: We thank the reviewer for their valuable suggestion. In response, we conducted the recommended study using isolated human liver microsomes with both INH and the lead compound 1d. The resulting data have been included in "Table 11" on page 17 of the revised manuscript. Corresponding revisions have also been made in the Results and Discussion sections of the main text.

Toxicity assessment in vivo: Conduct basic liver toxicity assays (e.g., serum ALT/AST levels, liver histopathology) in mice following repeat dosing of INH and lead prodrug(s) to confirm reduced hepatotoxicity.

Author's Response: The authors have conducted the recommended study using INH and the lead prodrug 1d. To assess the comparative impact of INH and 1d on liver injury markers (ALT/AST), a repeat-dose oral study was performed using higher doses of INH, 1d, and a vehicle control group. While INH treatment resulted in a significant increase in ALT and AST levels, no such elevation was observed in the 1d treatment group compared to the control. The results are provided in "Figure S48" on page S69 in the supporting information. Corresponding updates have been made to the results section on page 15 in the main text and the detailed methodology is provided in the Supporting Information. Unfortunately, liver histology could not be performed due to the lack of resources and expertise.

Assessment of pyridoxine (vitamin B6) depletion: Since INH is known to deplete B6 and cause peripheral neuropathy, evaluating plasma B6 levels post-dosing may help confirm reduced off-target reactivity.

Author's Response: The authors conducted this recommended study using liver tissue samples from the repeat-dose study. We attempted to quantify the levels of pyridoxal and pyridoxal-5′-phosphate; however, the differences in these metabolite levels between the two groups were not statistically significant. This information has been incorporated into the main text on page 15, results are provided as "Figure S49" in the supporting information along with the corresponding methodology.

Tissue distribution analysis of intact prodrug: Determine whether significant levels of unmetabolized prodrug (e.g., 1d) accumulate in tissues, which may pose long-term safety concerns.

Author's Response: The authors sincerely acknowledge the reviewer's insightful suggestion. It is indeed suspected that some accumulation of compound 1d in tissues may occur, given
its relatively high circulatory levels and reasonable plasma half-life following oral administration. However, we regret that we were unable to perform this analysis in the current study.

We recognize that 1d does not exhibit an ideal prodrug profile, and this limitation is acknowledged in the manuscript. Specifically, the final line of the Discussion section states: "As a next step, it will be important to determine the superiority of 1d or one of its analogs with a similar or better PK profile using well-designed animal efficacy and detailed safety studies."

The authors would also like to clarify that the primary aim of this manuscript is to explore whether appropriate labile masking of the terminal $-NH_2$ group can beneficially modulate the in vivo metabolic profile of INH. We believe the data presented in this study sufficiently supports conclusions related to this research question.

Additional Questions:

Quality of experimental data, technical rigor: Top 10%

Significance to chemistry researchers in this and related fields: Moderate

Broad interest to other researchers: Top 10%

Novelty: Moderate

Is this research study suitable for media coverage or a First Reactions (a News & Views piece in the journal)?: Yes

Reviewer: 2

Recommendation: Publish in ACS Central Science after minor revisions noted.

Comments:
The manuscript presents a novel approach to mitigate the adverse effects of isoniazid by designing carbamate-based prodrugs. This innovative strategy addresses a significant challenge in tuberculosis treatment. The study is methodologically sound and provides comprehensive data on the synthesis, stability, bioconversion, and pharmacokinetics of the prodrugs. The findings suggest that the lead compound (1d) offers improved systemic exposure and reduced formation of undesirable metabolites, potentially enhancing patient compliance and safety.

However, further studies in additional animal models are needed to confirm these results and fully demonstrate the potential of this strategy.

Overall, I have only a few minor remarks:
Author's Response: The authors express their gratitude for the appreciation of their work by the reviewer.

1) The study focuses on bioconversion by plasma enzymes. Why were hepatic enzymes not considered? For example, see lines 44–49 in the Introduction.

Author's Response: The authors acknowledge the reviewer's suggestion and have performed this study with INH and the lead prodrug 1d. The data is reported in "Table 11" on page 17 of the revised manuscript. Appropriate updates have also been made in the main text.

2) Instead of logP, the authors should use logD, as the compounds likely exist in both ionized and unionized forms. Please revise the discussion accordingly (the experimental values are logD, while the predicted values are likely logP).

Author's Response: As suggested by the reviewer, the authors have made necessary changes in the revised manuscript.

3) In the Chemistry section, please specify the method used to predict lipophilicity (ie. ChemDraw).

Author's Response: As suggested by the reviewer, the authors have made necessary changes in the revised manuscript.

4) The title “Intestinal and stomach...” on line 15 appears to be poorly aligned in the text.Author’s Response: As suggested by the reviewer, the authors have made the necessary changes in the revised manuscript.

5) The meaning of the values under eAUCs in Table 10 is unclear. Please clarify.

Author’s Response: In this study, the compounds were incubated with tissue homogenates to evaluate their metabolic conversion over a 20-hour period. Aliquots were collected at predefined time points between 0 and 20 hours. The extrapolated Area Under the Curve (eAUC) was calculated from the concentration-time profile of each analyte over the entire incubation period. This eAUC reflects the total analyte exposure and serves as an integrated measure of the cumulative release or formation of the analyte throughout the study duration.

Additional Questions:
Quality of experimental data, technical rigor: Top 10%

Significance to chemistry researchers in this and related fields: Top 10%

Broad interest to other researchers: Top 10%

Novelty: Top 10%

Is this research study suitable for media coverage or a First Reactions (a News & Views piece in the journal)?: No

Reviewer: 3

Recommendation: Publish in ACS Central Science after minor revisions noted.

Comments:
This manuscript presents a well-conceived and executed study on the design, synthesis, and pharmacokinetic evaluation of carbamate-based prodrugs of isoniazid (INH), aimed at mitigating its metabolism-mediated toxicity. The rationale—masking the terminal $-NH_2$ group to reduce the formation of hepatotoxic metabolites—is both scientifically sound and of high translational relevance, particularly in the context of improving tuberculosis treatment outcomes. The authors demonstrated that selected prodrugs, especially compound 1d, achieve superior systemic exposure to free INH with concomitant reduction in AcINH levels, one of the key metabolites. The development and application of a derivatization-free LC-MS method for simultaneous quantification of INH and its metabolites adds significant technical value to the study. The pharmacokinetic data, from both single and repeat-dose experiments, convincingly support the potential of this prodrug strategy to enhance INH's therapeutic index.

However, one aspect that could further strengthen this work is the inclusion of preliminary efficacy and safety data—particularly antibacterial activity and hepatotoxicity assessments. Although the pharmacokinetic improvements are promising, confirming that these translate into therapeutic benefits without compromising efficacy or introducing new toxicity would be crucial for advancing toward clinical relevance.
Overall, this is a high-quality contribution that addresses a longstanding challenge in TB chemotherapy. I recommend publication after consideration of the above points.

Author’s Response: The authors sincerely thank the reviewer for their kind appreciation of this work.

As suggested, a repeat-dose toxicity study was conducted using higher doses of INH and the lead compound 1d, with serum ALT and AST levels evaluated as markers of liver injury. INH treatment at higher doses resulted in a significant elevation of these markers, whereas no such increase was observed in the 1d-treated group. These findings suggest that 1d may possess a more favorable hepatic safety profile compared to INH.

In addition, the authors attempted to assess the depletion of pyridoxal and pyridoxal-5′phosphate in liver tissues following repeat dosing. However, the differences observed between the treatment groups were not statistically significant and thus did not yield a conclusive outcome. As noted above, the newly generated data and corresponding results have been incorporated into both the revised main text and the supporting information.

The authors also acknowledge the reviewer’s valuable suggestion regarding efficacy studies. However, conducting an animal efficacy study using an infected mouse model would require considerable time, ethical approvals, and dedicated resources, which are beyond the current scope of this work.

Additional Questions:
Quality of experimental data, technical rigor: Top 10%

Significance to chemistry researchers in this and related fields: Top 10%

Broad interest to other researchers: Top 10%

Novelty: Top 1%

Is this research study suitable for media coverage or a First Reactions (a News & Views piece in the journal)?: No

oc-2025-005769.R2

Name: Peer Review Information for "Labile masking of the free -NH2 group of isoniazid restricts in vivo metabolism and improves pharmacokinetic profile in mice; Design, synthesis and evaluation of carbamate-based prodrugs"

Second Round of Reviewer Comments

Reviewer: 3

Comments to the Author

In this reviewer's opinion, following the first round of revisions, the manuscript is suitable for acceptance and publication in ACS Central Science.

Reviewer: 2

Comments to the Author

The revised version addresses all the remarks and is good as it is.

Reviewer: 1

Comments to the Author

Publish as is.

Author's Response to Peer Review Comments:

Dear Editor,

Thank you very much for the opportunity. We sincerely appreciate the thoughtful and constructive feedback. As suggested, the authors have made the necessary revisions in the updated manuscript and supporting information.

Please find below our point-by-point responses for your consideration. As suggested, we are submitting a marked version of the revised manuscript and supporting information (with new changes highlighted in green for review purposes), along with the clean copy for each.

We truly hope that the revisions meet the expectations and are acceptable to progress for the next steps.

On behalf of all co-authors, I would like to express our sincere gratitude for your time, support, and consideration.

Best Regards,

Dinesh

A point-by-point response from the authors

1) The Title is too long and should be updated (also in the Supplementary Information file).

Please consider using one of these titles:

a) Labile Carbamate Prodrugs of Isoniazid for Improved Pharmacokinetics in Tuberculosis Treatment

b) Improved Pharmacokinetics of Isoniazid through Labile Carbamate-Based Prodrug Design

Author's response: As suggested, we have shortened the title. The new title is, "Carbamate Prodrugs Restrict In Vivo Metabolism and Improve the Pharmacokinetics of Isoniazid".

The necessary changes have been incorporated in the main manuscript as well as the supporting information in the revised drafts.

2) In Figure 1:

correct: "THF:Water" to "THF/H_2O" correct:
"rt, 1h -16h" to "rt, 1-16 h"

Author's response: As suggested, the necessary changes have been made in the revised draft for Figure 1.

The pyridinium salts should be presented correctly, as shown e.g., in this paper: https://pubs.rsc.org/en/content/articlepdf/2018/qo/c7qo00836h. Please make corrections also in the Supplementary Information file.

Author's response: The chemical structures of all compounds in Figure 1 are modified to represent the pyridinium salt. The necessary changes have also been made in the supporting file for all the structures that are pasted, along with their respective spectroscopic data.

Additionally, a correction was needed in the labelling of structures 1i and 1j. The 1i is an isobutyl carbamate and 1j is a phenolic carbamate. The two structures were somehow misplaced in relation to each other in the old draft. This correction was needed in Figure 1 only and has no bearing on the text of the manuscript or supporting file.

The numbering of compound 1a-1j should be in bold. The compounds should be presented as salts. The blue frame can be removed.

Author's response: The necessary changes have been made.

3) The current TOC graphic needs improvements. The resolution of the graph is low, and the axis labels are not clearly visible. The TOC (should be better in the frame) should contain a visual summary of the paper's key idea, designed to convey what the research is about in a clear, engaging, self-explanatory, and appealing way. We recommend checking out the

TOCs of some recently published articles to improve the TOC: https://pubs.acs.org/toc/acscii/current

Author's response: The authors have improved the TOC graphic. The new improved TOC has been placed on the last page of the manuscript. However, we were not able to upload a separate TIFF file during submission.

Formatting needs: As suggested, we have added a "Supporting Information" heading to the title page of your Supporting Information file.
